# Occurrence and Time of Onset of Intraventricular Hemorrhage in Preterm Neonates: A Systematic Review and Meta-Analysis of Individual Patient Data

**DOI:** 10.1001/jamapediatrics.2024.5998

**Published:** 2024-12-30

**Authors:** Zsuzsanna Nagy, Mahmoud Obeidat, Vanda Máté, Rita Nagy, Emese Szántó, Dániel Sándor Veres, Tamás Kói, Péter Hegyi, Gréta Szilvia Major, Miklós Garami, Ákos Gasparics, Arjan B. te Pas, Miklós Szabó

**Affiliations:** 1 Centre for Translational Medicine, Semmelweis University, Budapest, Hungary; 2 Department of Obstetrics and Gynecology, Semmelweis University, Budapest, Hungary; 3 Department of Neonatology, Pediatric Centre, Semmelweis University, Budapest, Hungary; 4 Pediatric Center, Semmelweis University, Budapest, Hungary; 5 Heim Pál National Pediatric Institute, Budapest, Hungary; 6 Institute for Translational Medicine, Medical School, University of Pécs, Pécs, Hungary; 7 Department of Biophysics and Radiation Biology, Semmelweis University, Budapest, Hungary; 8 Department of Stochastics, Institute of Mathematics, Budapest University of Technology and Economics, Budapest, Hungary; 9 Institute of Pancreatic Diseases, Semmelweis University, Budapest, Hungary; 10 Neonatology, Willem Alexander Children’s Hospital, Leiden University Medical Center Leiden, Leiden, the Netherlands

## Abstract

**Importance:**

Intraventricular hemorrhage (IVH) has been described to typically occur during the early hours of life (HOL); however, the exact time of onset is still unknown.

**Objective:**

To investigate the temporal distribution of IVH reported in very preterm neonates.

**Data Sources:**

PubMed, Embase, Cochrane Library, and Web of Science were searched on May 9, 2024.

**Study Selection:**

Articles were selected in which at least 2 cranial ultrasonographic examinations were performed in the first week of life to diagnose IVH. Studies with only outborn preterm neonates were excluded.

**Data Extraction And Synthesis:**

Data were extracted independently by 3 reviewers. A random-effects model was applied. This study is reported following the Preferred Reporting Items for Systematic Reviews and Meta-analyses (PRISMA) reporting guideline. The Quality in Prognostic Studies 2 tool was used to assess the risk of bias.

**Main Outcomes And Measures:**

The overall occurrence of any grade IVH and severe IVH among preterm infants was calculated along with a 95% CI. The temporal distribution of the onset of IVH was analyzed by pooling the time windows 0 to 6, 0 to 12, 0 to 24, 0 to 48, and 0 to 72 HOL. A subgroup analysis was conducted using studies published before and after 2007 to allow comparison with the results of a previous meta-analysis.

**Results:**

A total of 21 567 records were identified, of which 64 studies and data from 9633 preterm infants were eligible. The overall rate of IVH did not decrease significantly before vs after 2007 (36%; 95% CI, 30%-42% vs 31%; 95% CI, 25%-36%), nor did severe IVH (10%; 95% CI, 7%-13% vs 11%; 95% CI, 8%-14%). The proportion of very early IVH (up to 6 HOL) after 2007 was 9% (95% CI, 3%-23%), which was 4 times lower than before 2007 (35%; 95% CI, 24%-48%). IVH up to 24 HOL before and after 2007 was 44% (95% CI, 31%-58%) and 25% (95% CI, 15%-39%) and up to 48 HOL was 82% (95% CI, 65%-92%) and 50% (95% CI, 34%-66%), respectively.

**Conclusion And Relevance:**

This systematic review and meta-analysis found that the overall prevalence of IVH in preterm infants has not changed significantly since 2007, but studies after 2007 showed a later onset as compared with earlier studies, with only a small proportion of IVHs occurring before 6 HOL.

## Key Points

**Question:**

What are the occurrence and temporal distribution of intraventricular hemorrhage (IVH) in very preterm neonates during the first week of life?

**Findings:**

This systematic review and meta-analysis including 64 studies and 9633 preterm neonates found that the overall prevalence of IVH in preterm neonates has not changed significantly over the past 20 to 40 years. However, IVH earlier than 6 hours of life has been reduced to less than 10% of all IVH events.

**Meaning:**

These data suggest that although preventive measures have been implemented, IVH has occurred later but its prevalence has not been reduced.

## Teaser

This systematic review and meta-analysis assesses the time of onset for intraventricular hemorrhage in preterm neonates and incidence rates through time.

## Introduction

Preterm birth is one of the most challenging health problems, associated with high mortality and long-term disability. Intraventricular hemorrhage (IVH) is a common and the most significant early complication responsible for adverse outcomes in very preterm infants. Extreme alterations in perinatal hemodynamics, cerebral blood flow and oxygenation, as well as infections and proinflammatory states are associated with underlying mechanisms that contribute to the development of IVH.^1,2,3,4,5,6^ A 2022 meta-analysis by Lai et al^7^ found that the incidence of any IVH was 34.3%, whereas the incidence of severe IVH was 15.0% for infants with gestational age (GA) less than 28 weeks. Improvements in neonatal care may reduce the risk of both mortality and IVH. However, a reduction in mortality of extremely preterm infants at the highest risk of IVH may offset the expected decrease in IVH by an increase in the number of survivors of IVH.^2,3^ These 2 trends together may lead to a stable overall rate of IVH.^8^

As IVH is multifactorial, theoretically, the use of measures from a care bundle during different (antepartum, intrapartum, and postpartum) time windows has the greatest potential to reduce IVH. When the effectiveness of these interventions is assessed, it is essential to know when IVH occurs. Most IVH occurs during the first 3 days of life^9^; however, the exact onset is typically unknown in individual events.^10^ A 2014 meta-analysis found that 48% of all IVH events occurred during the first 0 to 6 hours of life (HOL) and 38% occurred after 24 HOL in neonates weighing 1500 g or less.^11^

Neuroimaging, particularly cranial ultrasonography, is the primary diagnostic tool for the detection of IVH. Repeated IVH screening at predetermined times using cranial ultrasonography provides an opportunity to detect the approximate time of onset of bleeding.^12^ Infrequent imaging may fail to capture the precise moment when IVH occurs, making it difficult to establish a cause-and-effect relationship between IVH and known risk factors.^13,14,15^ Furthermore, screening protocols for cranial ultrasonography show considerable heterogeneity among neonatal intensive care units.

The primary aim of our study was to explore the temporal distribution of the onset of IVH in preterm infants by a systematic review and meta-analysis of previous studies reporting IVH. The secondary aim was to test hypotheses that recent improvements in neonatal care were associated with a reduction in the prevalence of IVH and/or delayed onset of IVH. As there was no single therapeutic breakthrough innovation in neonatal medicine in recent years that would have provided a clearly identifiable time boundary, 2007 was used as the cutoff date for the time analysis. This also allowed comparison with a previous meta-analysis.^11^

## Methods

This systematic review and meta-analysis is reported following Meta-analysis of Observational Studies in Epidemiology (MOOSE) reporting guideline,^16^ the Cochrane Handbook,^17^ and the Preferred Reporting Items for Systematic Reviews and Meta-analyses (PRISMA) reporting guideline.^18^ The protocol was registered in PROSPERO (identifier CRD42022370884).^19^

### Eligibility Criteria

Cohort studies, case series, and clinical trial or conference abstracts were eligible; case reports and studies with only outborn preterm neonates were excluded from our systematic review. Our primary aim was to describe the occurrence and temporal distribution of the onset of IVH in preterm neonates (birth weight ≤1500 g and GA ≤32 weeks) during the first week of life. Studies in which cranial ultrasonography was performed at least twice during the first week of life to diagnose IVH were selected.

### Information Sources and Search Strategy

PubMed, Embase, Cochrane Library, and Web of Science were searched on May 9, 2024. Reference lists of eligible studies and review articles were also screened via citation chaser.^20^ No restrictions were applied. The search key included age, the disease in question (IVH), and a screening domain (eTable 1 in Supplement 1).

### Screening and Selection

After removing duplicates using reference management software (EndNote X9; Clarivate Analytics), Zs. N. and G. M. independently screened titles, abstracts, and full texts against predefined eligibility criteria. Cohen κ was used to assess interrater agreements during the selection process. A third author (M. Sz.) resolved any conflicts.

### Data Extraction

Three independent authors (Zs. N., G. M., and E. Sz.) extracted data from eligible studies into an Excel spreadsheet (Office 365; Microsoft). A fourth independent author (M. Sz.) resolved all disagreements. The following data were extracted from each eligible article or conference abstract: study name, first author, countries, publication year, study period, design and methods, total number of patients with any grade and with severe IVH, gestational age, birth weight, the onset of IVH, outborn status, antenatal steroid rate, and early and total mortality.

### Risk of Bias Assessment

The Quality in Prognostic Studies 2 (QUIPS-2) tool^21^ was used by 2 independent authors (Zs. N. and G. M.). Any disagreements were resolved by a third investigator (M. V.).

### Statistical Analysis

Statistical analyses were performed according to Harrer et al.^22^ All analyses were conducted using a random-effects model. The distribution of IVH onset time was analyzed by pooling the time windows 0 to 6, 0 to 12, 0 to 24, 0 to 48, and 0 to 72 HOL. A random intercept logistic regression approach was used to analyze the overall proportions of IVH and severe IVH.^23^ Results and pooled proportions with 95% CIs were visualized in forest plots. Prediction intervals were also calculated. A subgroup analysis was conducted using studies published before and after 2007 to allow comparison with the results of a previous meta-analyses.^11^ Heterogeneity was assessed using Higgins and Thompson *I*^2^ statistics. Statistical analyses were performed using R software version 4.1.2 (R Project for Statistical Computing) except for the interval-censored survival analysis, which was performed using SAS software. A detailed statistical description can be found in the eMethods in Supplement 1.

## Results

### Search and Selection

**Figure 1.**
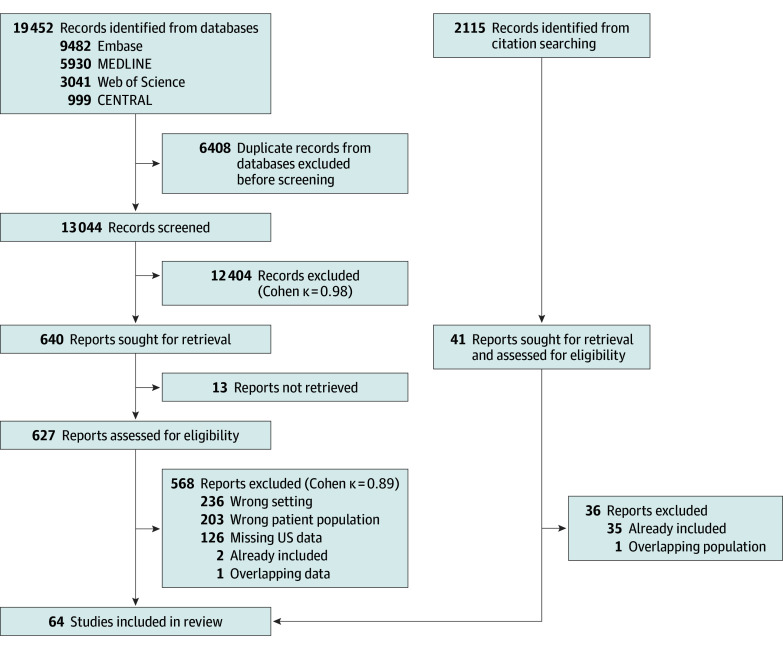
Flowchart of the Screening and Selection Process of the Studies

In total, 19 452 and 2115 records were identified by systematic search and citation chasing, respectively. Sixty-four studies^12,24,25,26,27,28,29,30,31,32,33,34,35,36,37,38,39,40,41,42,43,44,45,46,47,48,49,50,51,52,53,54,55,56,57,58,59,60,61,62,63,64,65,66,67,68,69,70,71,72,73,74,75,76,77,78,79,80,81,82,83^ were included in the quantitative analysis, 2 of which were included only in the calculation of the proportion of severe IVH occurrence among preterm neonates (Figure 1). All reasonable attempts were made to contact the authors for additional data. On the basis of their responses, data for more than 2000 individual patients from 14 articles^12,56,59,61,62,63,65,70,73,74,75,78,79^ were included to provide a more accurate estimate of the temporal distribution of IVH onset.

### Characteristics of Studies Included

In total, 9633 infants were included, mainly from Europe and North America. Most of the studies presented and analyzed were cohort studies (retrospective: 12 studies^25,29,57,58,64,71,76,77,78,81,83,84^ and prospective: 40 studies^12,24,26,27,28,32,33,34,36,37,38,39,40,42,44,45,46,47,48,51,52,54,55,56,59,60,61,62,65,66,67,68,70,72,74,75,79,80,82,85^). Severe IVH events were reported in 51 studies^12,24,25,26,27,28,29,31,33,34,35,36,37,38,39,40,42,44,45,46,47,48,49,52,53,54,55,56,57,59,61,63,64,65,68,69,70,71,72,73,74,75,76,77,78,79,80,81,82,85,86^ Baseline characteristics of the studies included are detailed in eTable 2 in Supplement 1.

### Proportions of Any Grade and Severe IVH

Data from 61 included studies^12,24,25,26,27,28,29,30,31,32,33,34,35,36,37,38,39,40,41,42,43,44,45,46,47,48,49,50,51,52,53,54,55,56,57,58,59,60,61,62,63,64,65,66,67,68,69,70,71,72,73,74,75,76,77,78,79,80,81,82,83,84,85,86^ of 9218 infants were used to calculate the overall occurrence of IVH among preterm neonates. Most of the articles selected for the before 2007 subgroup had been included in the previous meta-analysis.^11^

**Figure 2.**
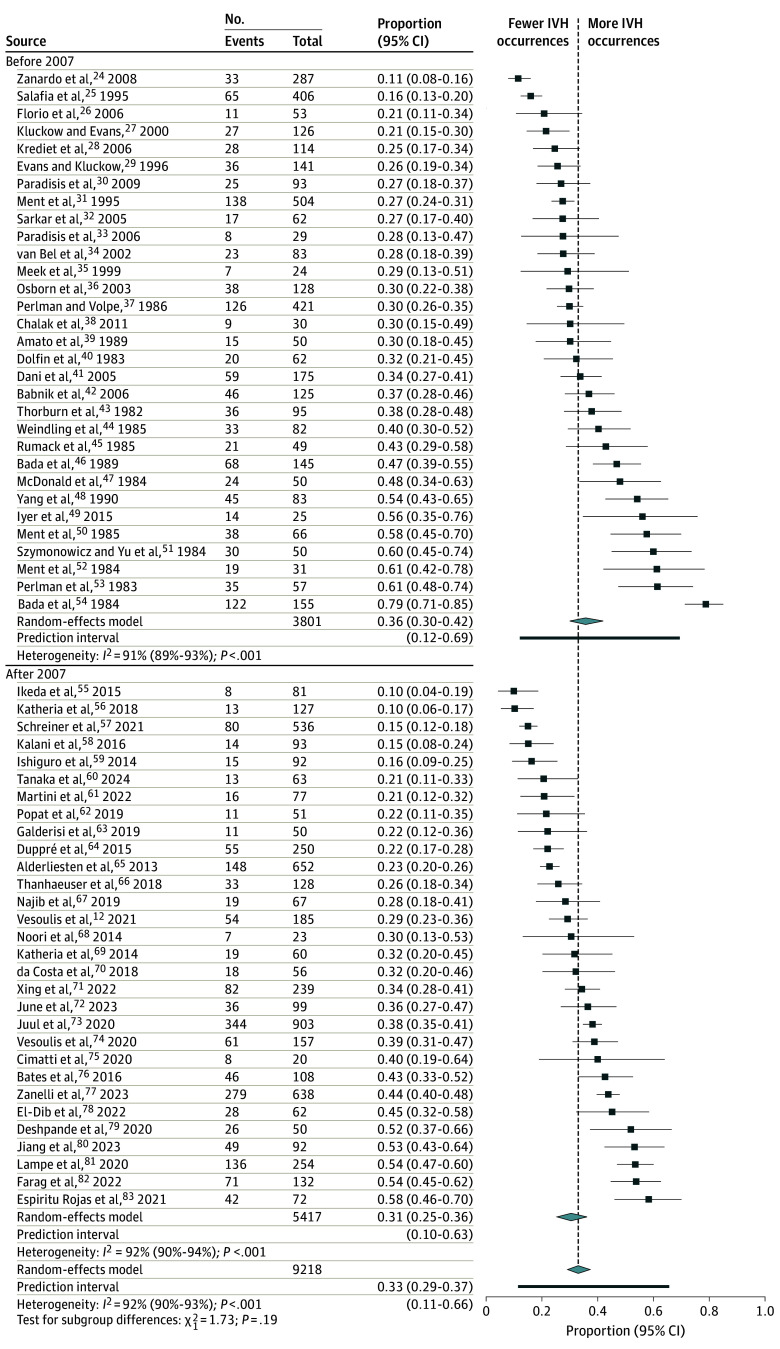
Overall Intraventricular Hemorrhage (IVH) Occurrence in Preterm Infants Before and After 2007 Diamonds indicate overall proportions.

**Figure 3.**
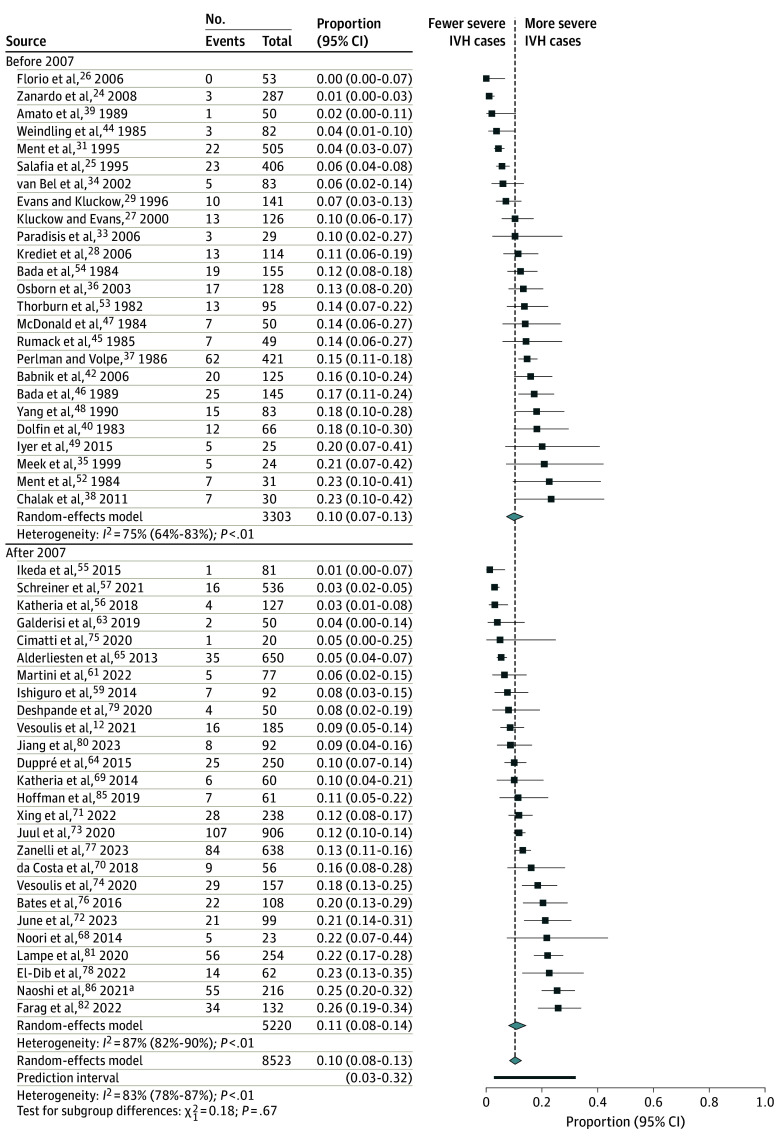
Overall Severe Intraventricular Hemorrhage (IVH) Occurrence in Preterm Infants Before and After 2007 Diamonds indicate overall proportions. ^a^Abstract presented at the 73rd Annual Congress of the Japan Society of Obstetrics and Gynecology.^86^

The overall occurrence of IVH in preterm infants was 33% (95% CI, 29%-37%), whereas for severe IVH, it was 10% (95% CI, 8%-13%). Neither the total occurrence of IVH (36% [95% CI, 30%-42%] vs 31% [95% CI, 25%-36%]; *P* = .19) nor the occurrence of severe IVH (10% [95% CI, 7%-13%] vs 11% [95% CI, 8%-14%]; *P* = .67) was different in subgroups before vs after 2007 (Figure 2 and Figure 3).

In the subgroups of gestational age, the studies before 2007 reported IVH occurrence of 33% (95% CI, 26%-41%) in infants with GA less than 28 weeks, 32% (95% CI, 22%-44%) in infants with GA less than 32 weeks, and 38% (95% CI, 29%-48%) among the noncategorized studies (eFigure 1 in Supplement 1). There was no significant difference in the 3 subgroups.

In the studies after 2007, IVH occurrence was 41% (95% CI, 31%-52%) in infants with GA less than 28 weeks, 27% (95% CI, 20%-36%) in infants with GA less than 32 weeks, and 28% (95% CI, 20%-39%) in noncategorized studies. The difference between subgroups of GA less than 28 and less than 32 was significant. This finding is consistent with the expectations that IVH is inversely associated with the upper limit of GA as an inclusion criterion for studies (eFigure 2 in Supplement 1).

Occurrence of severe IVH in infants with GA less than 28 weeks, GA less than 32 weeks, and among the noncategorized studies in studies before 2007 was 18% (95% CI, 6%-43%), 7% (95% CI, 3%-15%), and 10% (95% CI, 7%-15%) (*P* = .04), respectively, whereas after 2007, it was 17% (95% CI, 11%-26%) (*P* < .001), 8% (95% CI, 5%-12%) (*P* < .001), and 12% (95% CI, 7%-19%) (*P* < .001), respectively (eFigure 3 and eFigure 4 in Supplement 1). This finding is consistent with the expectations that the risk of severe IVH is inversely associated with GA.

### Proportion of Any Grade IVH in All Preterm Neonates by Postnatal Age

**Figure 4.**
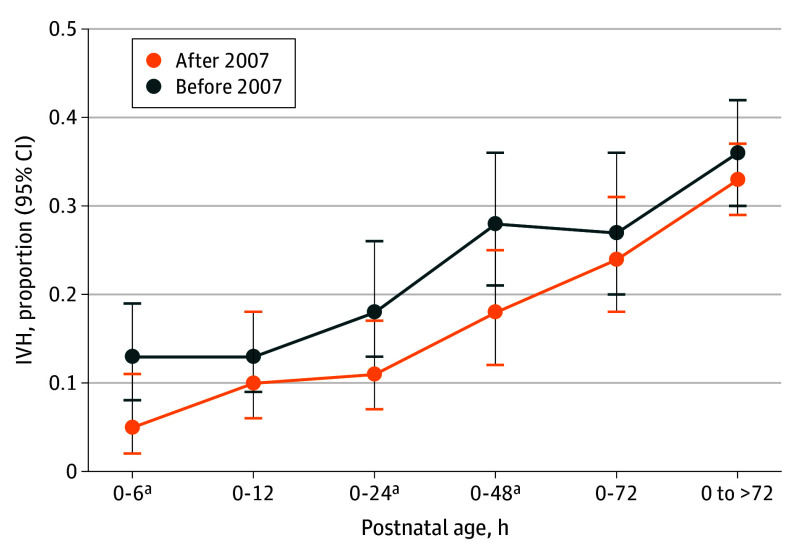
Intraventricular Hemorrhage (IVH) Rate Among All Preterm Infants by Postnatal Age ^a^*P* < .05.

The proportion of very early presenting IVH (up to 6 HOL) after 2007 was significantly lower than before 2007 (5% [95% CI, 2%-11%] vs 13% [95% CI, 8%-19%]). IVH up to 24 HOL before and after 2007 was 18% (95% CI, 13%-26%) and 22% (95% CI, 7%-17%) and up to 48 HOL, 28% (95% CI, 21%-36%) and 18% (95% CI, 12%-25%) among all preterm neonates, respectively. The proportions in the intervals 0 to 12 and 0 to 72, and beyond 72 HOL did not change significantly (Figure 4; eFigures 5-9 in Supplement 1). To highlight the accuracy of our results, 10^56,59,61,62,63,68,70,73,75,78^ of 13 studies contained individual data in the subgroup after 2007.

### Proportion of Any Grade IVH Among All IVH Events by Postnatal Age

**Figure 5.**
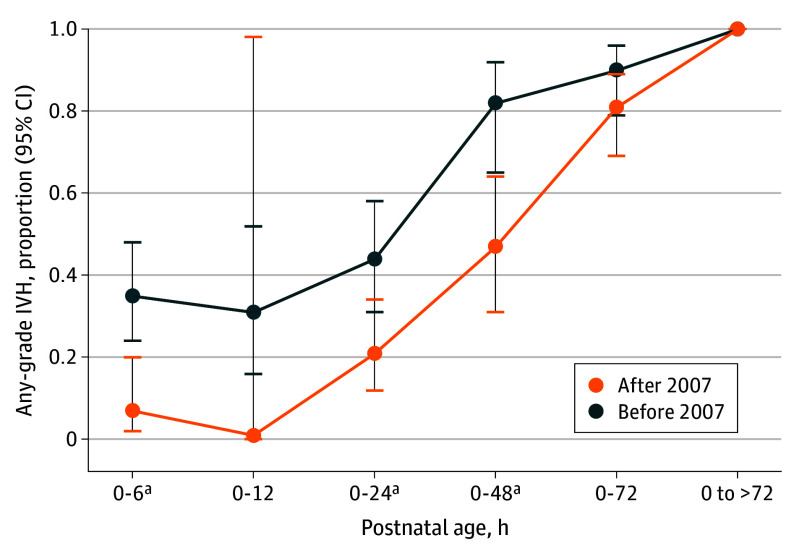
IVH Rate Among All Intraventricular Hemorrhage (IVH) Events by Postnatal Age ^a^*P* < .01.

The studies included reported IVH proportion in all IVH events before and after 2007 at different time intervals. The proportion of very early presenting IVH decreased significantly after 2007 (6 HOL: 35% [95% CI, 24%-48%] before 2007 vs 9% [95% CI, 3%-23%] after 2007; *P* = .002; 24 HOL: 44% [95% CI, 31%-58% before 2007 vs 25% [95% CI, 15%-39%] after 2007; *P* = .03; up to 48 HOL: 82% [95% CI, 65%-92%] before 2007 vs 50% [95% CI, 34%-66%] after 2007; *P* = .002). The remaining events occurred beyond 72 HOL (Figure 5; eFigures 10-14 in Supplement 1). To highlight the accuracy of our results, it should be emphasized that 6^59,61,62,63,68,75^ of 9 studies contained individual data in the subgroup after 2007.

### Differences in the Distribution Pattern of Onset of IVH of Any Grade by GA and Epochs

The distribution pattern showed no significant difference between neonates with GA less than 28 and GA less than 32 in studies after 2007 (eTable 3 and eFigures 15-19 in Supplement 1) nor in neonates with GA 32 weeks or less between the subgroups before and after 2007 (eFigures 20-24 in Supplement 1) nor in neonates with GA less than 28 weeks in the subgroups before and after 2007 (eFigures 25-29 in Supplement 1).

### Risk of Bias Assessment

Twenty-eight studies^24,25,27,28,30,31,33,34,35,36,37,39,45,46,49,50,51,53,54,57,58,60,66,67,71,76,85,86^ (44%) had an overall high risk of bias, whereas 17 studies^12,40,47,52,56,59,61,62,65,68,70,75,78,79,81,82,84^ (27%) had a low risk of bias. The primary reasons for the significant risk of bias were the absence of information on participation and the lack of information on follow-up time (eTable 4 in Supplement 1).

### Heterogeneity and Publication Bias

Variations in study methods, including differences in cranial ultrasonography timing and study populations, may explain the discrepancies in the exact timing of cranial ultrasonography. For this reason, we performed an additional subgroup analyses based on gestational age, early and total mortality, antenatal steroid use, outborn status, and exclusion criteria (eFigures 30-38 in Supplement 1). However, after all listed adjustments, heterogeneity remained unexplained.

Visualization and the corresponding Peter test *P* value suggest no publication bias in studies after 2007 (eFigure 40 and eFigure 42 in Supplement 1). However, for earlier studies, Peter test *P* value indicates potential publication bias (*P* = .04) (eFigure 39 and eFigure 41 in Supplement 1).

## Discussion

Our systematic review and meta-analysis found that although the occurrence of IVH in preterm infants did not change over time, the time of onset of IVH shifted toward later in life. The very early presentation of IVH now accounts for less than 10% of all IVH events, decreasing 4-fold (35% vs 9%) in the years 2007 to 2023 compared with 1981 to 2006. However, the overall occurrence of any grade IVH and severe IVH has remained unchanged.

Several factors play a role in the development of IVH, including antenatal, perinatal, and postnatal events. Although IVH is an acute event, the exact time of onset is unknown, as IVH is clinically silent^87^ in most events, and in many places, the first cranial ultrasonography is performed beyond the third postnatal day. Some experts consider that IVH occurring before 12 hours of life may be associated with antepartum or intrapartum events, whereas those occurring after 12 hours of life are often associated with other postnatal factors.^28,29,34,35,36,53^ Accordingly, the time of the onset of IVH is critical information that can help clinicians better understand the relationship between certain risk conditions and IVH and assess the value of preventive intervention.

Current recommendations on the timing and frequency of cranial ultrasonography do not support the diagnosis of the exact time of onset of IVH. The American Academy of Pediatrics recommends neurosonography in preterm infants within 7 days of birth.^88^ Of the 64 articles studied, 56 studies^12,24,26,27,28,30,31,32,33,34,35,36,37,38,39,40,41,42,43,44,45,46,47,48,49,50,51,52,53,54,55,56,57,59,60,61,62,63,64,65,66,68,69,72,73,74,75,76,78,79,80,81,82,83,84,86^ performed cranial ultrasonography within 24 HOL, and all 64 articles conducted cranial ultrasonography within 72 HOL. Some experts have emphasized the importance of detecting the onset of IVH by performing cranial ultrasonography several times during the first week rather than only once on days 7 to 10, arguing that this allows a better delineation of the timing of injury and identification of antecendence.^89^

In a comprehensive analysis by Lai et al^7^ using data from 2010 to 2020, the incidence of IVH was 34.3%, and the incidence of severe IVH was 15.0% for infants with GA less than 28 weeks. The Vermont Oxford Network reported in 2023 that the rates of IVH and severe IVH remained stable since 2012.^90^ Our study is not epidemiological, but our data are comparable to these epidemiological reports, indicating that we elaborate field data.

Al-Abdi et al^11^ reported a higher proportion of IVH in preterm infants up to 6 HOL before 2007. This may be explained by differences in the study population, exclusion, and inclusion criteria. Our study was more stringent, excluding certain articles^31,36^ and including others^26,30,40,50,52^ compared with the study by Al-Abdi et al.^11^ Due to relatively minor differences and the historical nature of the results before 2007, we do not consider this clinically relevant. In a 2019 study, Leiser et al^91^ reported that the onset of IVH occurs at a mean of 24 to 48 hours after delivery, with approximately 10% developing within 12 hours after birth, which is consistent with our findings.

The unchanged overall prevalence of IVH may be surprising. There are several possible explanations for this result. One is that with advances in neonatology, although the incidence of IVH may have decreased in infants with GA 26 to 28 weeks, this may have been offset by an increase in more vulnerable infants with GA 22 to 25 weeks with a higher incidence of IVH. Thus, for the cohort as a whole, the rate of IVH did not change significantly. This explanation is partially supported by Horbar et al,^92^ who reported that between 1997 and 2021, the mortality rate for extremely preterm infants decreased from 18.1% to 12.4% and distributions of infants by week of GA remained stable over the period. Similarly, our meta-analysis found that the pooled mean GA did not differ significantly between the 2 periods. However, we could not detect a similar reduction in mortality, as most of the articles included did not provide data on mortality and IVH by GA subgroups.

A further explanation may be that not all preventive measures to reduce IVH are currently well developed or consistently applied. However, the reduction in early IVH is an encouraging result. This may indicate a more consistent use of procedures with evidence of beneficial effects, eg, antenatal steroids, magnesium sulfate, delayed and physiological-based cord clamping, and thermal protection. Our meta-analysis is not well suited to prove this explanation in detail. However, it should be noted that all these measures may not be sufficient to completely eliminate and prevent IVH. Thus, the risk and susceptibility to IVH persist in the early days of life and may still occur due to respiratory and circulatory perturbation, pain, or other environmental stressors that are not yet sufficiently controlled.

### Implications for Practice and Future Research

High heterogeneity was observed across studies, suggesting a need for standardized reporting of cranial ultrasonography and accurate documentation of risk factors for each patient. We hypothesize that, due to the multifactorial etiology of IVH, timely detection of IVH in preterm infants may help us to gain a deeper understanding of the specific roles of each contributing factor and pathomechanism. Accurate, timely diagnosis of IVH is possible with an increased frequency of cranial ultrasonography during the early days of life. However, this is contrary to the recommended minimal handling of the infants. Other neurointensive monitoring tools, such as electroencephalography,^49^ near-infrared spectroscopy,^56,62,65,68,75,78,79^ ongoing evolution of ultrasonographic technology,^93^ or a combination of these, may promise more accurate, timely detection of IVH in the future. The translation of scientific research findings into clinical practice is crucial for the benefit of both clinicians and patients.^94,95^

### Strengths and Limitations

In terms of strengths, our analysis followed a preregistered protocol, applying a rigorous method. The main strength of this study is the large amount of individual data used. It is essential to acknowledge that the time of detection is not equal to the real-time onset; however, on the basis of our results, it is reasonable to assert that before 6 HOL is a sufficiently narrow time window to support clinically relevant accuracy in detecting changes in the development of IVH.

This study also has some limitations. Several factors may influence the prevalence and the time of onset of IVH, such as GA, antenatal steroids, birth setting, and resuscitation protocol, along with different exclusion criteria and mortality reporting.^96,97,98^ However, this study could not explore the specific role of these factors in the variation of IVH in different studies. Some experts point out that improved ultrasonography equipment with increased resolution allows the detection of more grade 1 IVH events.^91^ If advanced cranial ultrasonography techniques have indeed allowed more accurate detection of IVH in recent years, this rather supports our observation that the proportion of very early IVH has declined robustly, while a small reduction in overall IVH may be missed.

## Conclusions

The findings of this systematic review and meta-analysis indicate that the overall prevalence of IVH did not change significantly over the past 20 to 40 years; however, the very early presenting form of IVH declined substantially. Less than 10% of all IVH in preterm infants occurred before 6 hours of life. These data suggest that recently implemented preventive measures have the potential to postpone rather than reduce IVH in very preterm infants.

## Notes

Supplement 1.
eMethods.
**eTable 1.** Search Key **eTable 2.** Baseline Characteristics of the Included Studies (n=64) **eTable 3.** IVH Rate Among GA<28 and GA<32 Weeks Infants by Postnatal Age After 2007 **eTable 4.** Risk of Bias Assessment Using Quality in Prognostic Studies 2 (QUIPS-2) Tool **eFigure 1.** Any Grade IVH Proportion by GA Limits in GA<28, GA<32 and Noncategorized Studies Before 2007 **eFigure 2.** Any Grade IVH Proportion by GA Limits in GA<28, GA<32, and Noncategorized Studies After 2007 **eFigure 3.** Proportion of Severe IVH by GA Limits in GA<28, GA<32, Noncategorized Subgroups Before 2007 **eFigure 4.** Proportion of Severe IVH by GA Limits in GA<28, GA<32, and Noncategorized Studies After 2007 **eFigure 5.** IVH Rate Up to 6 HOL of All Preterm Infants in the Subgroups Before and After 2007 **eFigure 6.** IVH Rate Up to 12 HOL of All Preterm Infants in the Subgroups Before and After 2007 **eFigure 7.** IVH Rate Up to 24 HOL of All Preterm Infants in the Subgroups Before and After 2007 **eFigure 8.** IVH Rate Up to 48 HOL of All Preterm Infants in the Subgroups Before and After 2007 **eFigure 9.** IVH Rate Up to 72 HOL of All Preterm Infants in the Subgroups Before and After 2007 **eFigure 10.** IVH Rate Up to 6 HOL in All IVH Cases in the Subgroups Before and After 2007 **eFigure 11.** IVH Rate Up to 12 HOL in All IVH Cases in the Subgroups Before and After 2007 **eFigure 12.** IVH Rate Up to 24 HOL in All IVH Cases in the Subgroups Before and After 2007 **eFigure 13.** IVH Rate Up to 48 HOL in All IVH Cases in the Subgroups Before and After 2007 **eFigure 14.** IVH Rate Up to 72 HOL in All IVH Cases in the Subgroups Before and After 2007 **eFigure 15.** IVH Rate Up to 6 HOL by GA Limits in GA<28, GA<32 in the Subgroup After 2007 **eFigure 16.** IVH Rate Up to 12 HOL by GA Limits in GA<28, GA<32 in the Subgroup After 2007 **eFigure 17.** IVH Rate Up to 24 HOL by GA Limits in GA<28, GA<32 in the Subgroup After 2007 **eFigure 18.** IVH Rate Up to 48 HOL by GA Limits in GA<28, GA<32 in the Subgroup After 2007 **eFigure 19.** IVH Rate Up to 72 HOL by GA Limits in GA<28, GA<32 in the Subgroup After 2007 **eFigure 20.** IVH Rate Up to 6 HOL in GA<32 in the Subgroups Before and After 2007 **eFigure 21.** IVH Rate Up to 12 HOL in GA<32 in the Subgroups Before and After 2007 **eFigure 22.** IVH Rate Up to 24 HOL in GA<32 in the Subgroups Before and After 2007 **eFigure 23.** IVH Rate Up to 48 HOL in GA<32 in the Subgroups Before and After 2007 **eFigure 24.** IVH Rate Up to 72 HOL in GA<32 in the Subgroups Before and After 2007 **eFigure 25.** IVH Rate Up to 6 HOL in GA<28 in the Subgroups Before and After 2007 **eFigure 26.** Overall IVH Rate Up to 12 HOL in GA<28 in the Subgroups Before and After 2007 **eFigure 27.** IVH Rate Up to 24 HOL in GA<28 in the Subgroups Before and After 2007 **eFigure 28.** Overall IVH Rate Up to 48 HOL in GA<28 in the Subgroups Before and After 2007 **eFigure 29.** Overall IVH Rate Up to 72 HOL in GA<28 in the Subgroups Before and After 2007 **eFigure 30.** IVH Rate by Before and After 2007 Exclusion Criteria of Only Congenital Anomaly Among Preterm Neonates **eFigure 31.** IVH Rate by Before and After 2007 Exclusion Criteria of Other Than Only Congenital Anomaly Among Preterm Neonates **eFigure 32.** sIVH Rate by Exclusion Criteria of Only Congenital Anomaly Among Preterm Neonates in the Subgroups Before and After 2007 **eFigure 33.** sIVH Rate by Exclusion Criteria of Other Than Only Congenital Anomaly Among Preterm Neonates in the Subgroups Before and After 2007 **eFigure 34.** Antenatal Steroid Rate in the Subgroups Before and After 2007 **eFigure 35.** Outborn Rate in the Subgroups Before and After 2007 **eFigure 36.** The Pooled Mean Gestational Age Within Subgroups With Study Data Periods Before and After 2007. **eFigure 37.** Early Mortality in the Subgroups Before and After 2007 **eFigure 38.** Total Mortality in the Subgroups Before and After 2007 **eFigure 39.** Funnel Plot of the Overall IVH in the Subgroup Before 2007 **eFigure 40.** Funnel Plot of the Overall IVH in the Subgroup After 2007 **eFigure 41.** Peter’s Test in the Subgroup Before 2007 **eFigure 42.** Peter’s Test in the Subgroup After 2007
eReferences.

Supplement 2.
Data Sharing Statement
