# Supplementary material for: Occurrence and Time of Onset of Intraventricular Hemorrhage in Preterm Neonates: A Systematic Review and Meta-Analysis of Individual Patient Data
Source: JAMA Pediatr. 2024 Dec 30;179(2):145–54. doi: 10.1001/jamapediatrics.2024.5998 (PMC11791718; doi:10.1001/jamapediatrics.2024.5998)
Supplement: Supplement 1. — eMethods. eTable 1. Search Key eTable 2. Baseline Characteristics of the Included Studies (n=64) eTable 3. IVH Rate Among GA<28 and GA<32 Weeks Infants by Postnatal Age After 2007 eTable 4. Risk of Bias Assessment Using Quality in Prognostic Studies 2 (QUIPS-2) Tool eFigure 1. Any Grade IVH Proportion by GA Limits in GA<28, GA<32 and Noncategorized Studies Before 2007 eFigure 2. Any Grade IVH Proportion by GA Limits in GA<28, GA<32, and Noncategorized Studies After 2007 eFigure 3. Proportion of Severe IVH by GA Limits in GA<28, GA<32, Noncategorized Subgroups Before 2007 eFigure 4. Proportion of Severe IVH by GA Limits in GA<28, GA<32, and Noncategorized Studies After 2007 eFigure 5. IVH Rate Up to 6 HOL of All Preterm Infants in the Subgroups Before and After 2007 eFigure 6. IVH Rate Up to 12 HOL of All Preterm Infants in the Subgroups Before and After 2007 eFigure 7. IVH Rate Up to 24 HOL of All Preterm Infants in the Subgroups Before and After 2007 eFigure 8. IVH Rate Up to 48 HOL of All Preterm Infants in the Subgroups Before and After 2007 eFigure 9. IVH Rate Up to 72 HOL of All Preterm Infants in the Subgroups Before and After 2007 eFigure 10. IVH Rate Up to 6 HOL in All IVH Cases in the Subgroups Before and After 2007 eFigure 11. IVH Rate Up to 12 HOL in All IVH Cases in the Subgroups Before and After 2007 eFigure 12. IVH Rate Up to 24 HOL in All IVH Cases in the Subgroups Before and After 2007 eFigure 13. IVH Rate Up to 48 HOL in All IVH Cases in the Subgroups Before and After 2007 eFigure 14. IVH Rate Up to 72 HOL in All IVH Cases in the Subgroups Before and After 2007 eFigure 15. IVH Rate Up to 6 HOL by GA Limits in GA<28, GA<32 in the Subgroup After 2007 eFigure 16. IVH Rate Up to 12 HOL by GA Limits in GA<28, GA<32 in the Subgroup After 2007 eFigure 17. IVH Rate Up to 24 HOL by GA Limits in GA<28, GA<32 in the Subgroup After 2007 eFigure 18. IVH Rate Up to 48 HOL by GA Limits in GA<28, GA<32 in the Subgroup After 2007 eFigure 19. IVH Rate Up to 72 HOL by [file jamapediatr-e245998-s001.pdf]

Supplemental Online Content

Nagy Zs, Obeidat M, Máté V, et al. Occurrence and time of onset of intraventricular hemorrhage in preterm neonates: a systematic review and meta-analysis of individual patient data. *JAMA Pediatr*. Published online December 30, 2024.
doi:10.1001/jamapediatrics.2024.5998

eMethods.
eTable 1. Search Key
eTable 2. Baseline Characteristics of the Included Studies (n=64)
eTable 3. IVH Rate Among GA<28 and GA<32 Weeks Infants by Postnatal Age After 2007
eTable 4. Risk of Bias Assessment Using Quality in Prognostic Studies 2 (QUIPS-2) Tool
eFigure 1. Any Grade IVH Proportion by GA Limits in GA<28, GA<32 and Noncategorized Studies Before 2007
eFigure 2. Any Grade IVH Proportion by GA Limits in GA<28, GA<32, and Noncategorized Studies After 2007
eFigure 3. Proportion of Severe IVH by GA Limits in GA<28, GA<32, Noncategorized Subgroups Before 2007
eFigure 4. Proportion of Severe IVH by GA Limits in GA<28, GA<32, and Noncategorized Studies After 2007
eFigure 5. IVH Rate Up to 6 HOL of All Preterm Infants in the Subgroups Before and After 2007
eFigure 6. IVH Rate Up to 12 HOL of All Preterm Infants in the Subgroups Before and After 2007
eFigure 7. IVH Rate Up to 24 HOL of All Preterm Infants in the Subgroups Before and After 2007
eFigure 8. IVH Rate Up to 48 HOL of All Preterm Infants in the Subgroups Before and After 2007
eFigure 9. IVH Rate Up to 72 HOL of All Preterm Infants in the Subgroups Before and After 2007
eFigure 10. IVH Rate Up to 6 HOL in All IVH Cases in the Subgroups Before and After 2007
eFigure 11. IVH Rate Up to 12 HOL in All IVH Cases in the Subgroups Before and After 2007
eFigure 12. IVH Rate Up to 24 HOL in All IVH Cases in the Subgroups Before and After 2007
eFigure 13. IVH Rate Up to 48 HOL in All IVH Cases in the Subgroups Before and After 2007
eFigure 14. IVH Rate Up to 72 HOL in All IVH Cases in the Subgroups Before and After 2007
eFigure 15. IVH Rate Up to 6 HOL by GA Limits in GA<28, GA<32 in the Subgroup After 2007
eFigure 16. IVH Rate Up to 12 HOL by GA Limits in GA<28, GA<32 in the Subgroup After 2007
eFigure 17. IVH Rate Up to 24 HOL by GA Limits in GA<28, GA<32 in the Subgroup After 2007
eFigure 18. IVH Rate Up to 48 HOL by GA Limits in GA<28, GA<32 in the Subgroup After 2007
eFigure 19. IVH Rate Up to 72 HOL by GA Limits in GA<28, GA<32 in the Subgroup After 2007
eFigure 20. IVH Rate Up to 6 HOL in GA<32 in the Subgroups Before and After 2007

© 2024 Nagy Zs et al. *JAMA Pediatrics.*

eFigure 21. IVH Rate Up to 12 HOL in GA<32 in the Subgroups Before and After 2007
eFigure 22. IVH Rate Up to 24 HOL in GA<32 in the Subgroups Before and After 2007
eFigure 23. IVH Rate Up to 48 HOL in GA<32 in the Subgroups Before and After 2007
eFigure 24. IVH Rate Up to 72 HOL in GA<32 in the Subgroups Before and After 2007
eFigure 25. IVH Rate Up to 6 HOL in GA<28 in the Subgroups Before and After 2007
eFigure 26. Overall IVH Rate Up to 12 HOL in GA<28 in the Subgroups Before and After 2007
eFigure 27. IVH Rate Up to 24 HOL in GA<28 in the Subgroups Before and After 2007
eFigure 28. Overall IVH Rate Up to 48 HOL in GA<28 in the Subgroups Before and After 2007
eFigure 29. Overall IVH Rate Up to 72 HOL in GA<28 in the Subgroups Before and After 2007
eFigure 30. IVH Rate by Before and After 2007 Exclusion Criteria of Only Congenital Anomaly Among Preterm Neonates
eFigure 31. IVH Rate by Before and After 2007 Exclusion Criteria of Other Than Only Congenital Anomaly Among Preterm Neonates
eFigure 32. sIVH Rate by Exclusion Criteria of Only Congenital Anomaly Among Preterm Neonates in the Subgroups Before and After 2007
eFigure 33. sIVH Rate by Exclusion Criteria of Other Than Only Congenital Anomaly Among Preterm Neonates in the Subgroups Before and After 2007
eFigure 34. Antenatal Steroid Rate in the Subgroups Before and After 2007
eFigure 35. Outborn Rate in the Subgroups Before and After 2007
eFigure 36. The Pooled Mean Gestational Age Within Subgroups With Study Data Periods Before and After 2007.
eFigure 37. Early Mortality in the Subgroups Before and After 2007
eFigure 38. Total Mortality in the Subgroups Before and After 2007
eFigure 39. Funnel Plot of the Overall IVH in the Subgroup Before 2007
eFigure 40. Funnel Plot of the Overall IVH in the Subgroup After 2007
eFigure 41. Peter's Test in the Subgroup Before 2007
eFigure 42. Peter's Test in the Subgroup After 2007
eReferences.

This supplemental material has been provided by the authors to give readers additional information about their work.

© 2024 Nagy Zs et al. *JAMA Pediatrics.*

eMethods.

Statistical analysis

Meta-analysis of the occurrence of IVH at 0-6, 0-12, 0-24, 0-48, and 0-72 hours was challenging, as often there were not complete data on the number of IVH cases in the selected time interval, e.g. due to differences in study timing or reporting problems. The applied time window definitions refer to the period when the IVH occurred prior to the earliest detection. The available data provide only lower and upper bounds for when the actual event occurred; therefore, only interval-censored data was available. We used the nonparametric maximum likelihood approach implemented in the reliability process of SAS software, following the advice of Bogaerts et al.[1], to obtain an occurrence probability estimate with standard error. The classical inverse variance meta-analysis was applied with a restricted maximum likelihood (REML) tau estimator on the logit transformed ratios to obtain pooled occurrence rates. The standard error of the logit transformed ratio was calculated using the delta method.

For each time interval of the form [0,t], those studies, including the exact occurrence numbers, were collected, and pooled proportion was calculated using the approach of Stijnen et al.[2] on the resulting study set. The sensitivity analysis provided a similar pattern to that of the main analyses.

The restricted dataset was also useful for the analysis of the occurrence rates among all IVH cases. For each time interval of the form [0,t] and each study including the exact occurrence number, the number of IVH occurrences in the time interval was divided by the total IVH cases reported in the study. Then, the resulting proportions were meta-analyzed using the approach of Stijnen et al.[2]

Explorative analysis was performed in order to explain heterogeneity based on inclusion criteria and the publication year before and after 2007, and the GA (GA<28, GA<32, non-categorizable like GA<29, GA<30, BW<1500g, BW<1250g).

To assess potential differences between studies with data periods before and after 2007, as a supplementary analysis, we compared mortality, out-born ratio, antenatal steroid ratio, and gestational age across the two periods. To meta-analyze mean differences, we used the classical inverse variance approach with a REML tau estimator. Frequently, mean and standard deviation were not available. We used the default method of the metacont R function to estimate mean and standard deviation from median, lower, and upper quartiles or/and min and max. When gestational age data were reported separately in two distinct groups, we used formula[3] to get the combined outcome. When only the mean was available and the sample size was large, we imputed the value 2 for standard deviation.

The data used in the meta-calculations can be divided into three categories: directly available in a study, calculated from data available in a study, and calculated from raw data received from the authors of a study. Due to this mixed nature of the input data, publication bias analysis was performed only for the overall IVH cases separately in the "before 2007" and "after 2007" subgroups by creating modified funnel plots (the y-axis represents study size) and by performing Peter's test.

© 2024 Nagy Zs et al. *JAMA Pediatrics.*

eTable 1. Search key in the databases (MEDLINE, Embase, CENTRAL, and Web of Science)

(Infant OR Premature OR prematur* OR preterm OR preterms OR LBW OR "low birth weight" OR ELBW OR "periviable" OR "extremely low gestational age neonate" OR Infant OR Premature OR prematur* OR preterm OR preterms OR LBW OR "low birth weight" OR ELBW OR "periviable" OR "extremely low gestational age neonate" OR ELGAN) AND
("Intracranial Hemorrhages" OR "Intracranial Hemorrhag*" OR "Intracranial Haemorrhag*" OR "Intra-cranial Hemorrhag*" OR "Intra-cranial Haemorrhag*" OR "brain Hemorrhag*" OR "brain Haemorrhag*" OR "cerebral Hemorrhag*" OR "cerebral Haemorrhag*" OR "Intraventricular Hemorrhag*" OR "Intraventricular Haemorrhag*" OR "Intra-ventricular Hemorrhag*" OR "Intra-ventricular Haemorrhag*" OR ICH OR IVH) AND
(Ultrasonography OR Ultraso* OR "cranial ultrasound" OR time OR onset)

eTable 2. a. Baseline characteristics of the included neonatal studies

Study	Country	Study design	Study period	Population Gestational age, wk/Birth weight, g	Mean±SD Gestational age	Median [IQR] Gestational age	Sample size	Patients with any grade IVH	Patients with severe IVH	Onset interval in hours (HOL) or days of life (DOL)	Patients number with any IVH
*Alderliesten at el. (2013)[4]	The Netherlands	case-control (subanalysis of prosp. obs.)	2009 - 2010	≤32	NA	IVH G1:27[5]<br>IVH G2: 27[4]<br>IVH G3:27[6]<br>IVH G4:281]	650	146	35	0-12	61
										individual data in hours and minutes	30
										12-168	55
*Noori et al. (2014)[5]	USA	prospective observational	2009 - 2010	23-27	25.9±1.2	NA	23	7	5	0-6	1
										28-40	3
										40-52	2
										72-672	8
Sarkar et al. (2005)[6]	USA	prospective observational	NA	≤28	26.2±1.6	NA	62	17	NA	0-0.5	5
										0.5-24	0
										24-72	4
										72-672	8
Krediet et al. (2006)[7]	The Netherlands, USA	prospective observational	2002 - 2004	≤32	NoRDS: 30.1± 1.7<br>RDS:29.0±1	NA	114	28	13	0-11	16
										12-discharge	9
										4-36	1
										8-36	1
										11-36	1
	Italy			<32	NA	IVH:	20	8	1	0-12	0

© 2024 Nagy Zs et al. *JAMA Pediatrics.*

*Cimatti et al. (2020)[8]		prospective, pilot	2016 - 2017			25.4[25–26.7] NoIVH: 29.5[27–32.3]				12-24	1
										12-30	1
										18-30	1
										24-32	1
										28-48	1
										24-48	1
										30-48	1
										48-72	1
*Deshpande et al. (2020)[9]	Canada	prospective observational cohort	2016 - 2017	23-28	NA	25[8]	50	26	4	individual data in hours and minutes	
*El-Dib et al. (2022)[10]	USA	retrospective cohort	2018 - 2020	<28	NA	NoIVH:26.1[25.3–27.1] IVH:25.1[24.1–26.1]	62	28	14	individual data in hours and minutes	
Babnik et al. (2006)[11]	Slovenia	prospective observational	2000 - 2002	23-29	Control:27.3±1.5, EarlyIVH: 26.7±1.8, LateIVH:26.7±1.8	NA	125	46	20	0-24	25
										24-discharge	12
										24-168	9
Bada et al. (1984)[12]	USA	prospective	1981 - 1982	<1500	30.6 ± 2.2 NoIVH:32 ±2, earlyIVH:30±2, lateIVH:31±2	NA	155	122	19	0-24	85
										24-168	37
Chalak et al. (2011)[13]	USA	prospective pilot	2007	23-28 <1000	25±2	NA	30	9	7	0-13	1
										0-120	2
										24-120	6
Dani et al. (2005)[14]	Italy	RCT		<28	Ibuprofen:25.3±1.2,	NA	175	59	NA	0-6	29
										6-24	22

© 2024 Nagy Zs et al. *JAMA Pediatrics.*

			2002 - 2003		placebo:25.9± 1.1					24-48	6
										48-168	2
Dolfin et al. (1983)[15]	Canada	prospective	1981 - 1982	<32	NA	NA	62	20	12	0-6	4
										6-12	2
										12-24	5
										24-48	6
										48-62	3
										>72	0
Farag et al. (2022)[16]	Egypt	prospective observational	2020 - 2021	≤32 ≤1500	NoIVH:30.3±1.4, IVH:29.3 ± 1.6	NA	132	71	34	0-24	41
										24-72	20
										72-168	10
Ikeda et al. (2014)[17]	Japan	prospective observational	2010 - 2013	<1000	26.5±2.4	NA	81	8	1	0-8	0
										8-16	1
										40-48	2
										48-56	1
										64-72	2
										88-92	1
										92-96	1
										96-104	0
*Ishiguro et al. (2014)[18]	Japan	prospective observational	2008 - 2012	<1500	NA	IVH:26.0 NoIVH:27.6	92	15	7	0-6	3
										6-24	1
										7-22	1
										16-32	1
										22-33	1
										24-47	1
										26-30	1

© 2024 Nagy Zs et al. *JAMA Pediatrics.*

										33-68	1
										62-71	1
										125-144	1
										168-192	1
										192-336	1
										46-68	1
Katheria et al. (2014)[19]	California, San Diego	RCT	2010-2013	<32	UCM 28 ±2 ICC 28±3	NA	60	19	6	0-6	1
										6-72	18
*Katheria et al. (2019)[20]	California, San Diego	prospective observational	2015-2016	<32	NoIVH/death:28 ±2, IVH/death:26 ± 2	NA	127	13	4	0-4	1
										0-4.5	2
										0-11	1
										0-12	1
										0-26	2
										0-56	1
										0-23	1
										0-44	1
										12.5-60	1
										12.5-62	1
										13-37	1
Meek et al. (1998)[21]	UK	NA	NA	24-31	NA	NA	24	7	5	0-24	2
										24-48	4
										48-72	1
Kluckow et al. (2000)[22]	Australia	prospective observational	1995-1996	23-29	27	NA	126	27	13	0-4	7
										4-8	2
										9-16	1
										16-24	1

© 2024 Nagy Zs et al. *JAMA Pediatrics.*

										24-32	3
										32-40	3
										40-48	1
										48-56	2
										56-64	1
										64-72	1
										168-192	2
McDonald et al. (1984)[23]	USA	prospective observational	1980 - 1981	<33	NoIVH: 29.3 ±1.7<br>IVH G1:29.4<br>IVH G2-4:27 ± 2	NA	50	24	7	0-4	7
										4-8	2
										9-16	1
										16-24	1
										24-32	3
										32-40	3
										40-48	1
										48-56	2
										56-64	1
										64-72	1
										168-192	2
Ment et al. (1984)[24]	USA	prospective observational	1982 - 0983	25-33<br>600-1250	28.3	NA	31	19	7	0-6	8
										6-18	2
										18-30	4
										30-66	0
										66-78	2
										78-96	1
										96-120	1
										168-336	1

© 2024 Nagy Zs et al. *JAMA Pediatrics.*

Ment et al. (1994)[25]	USA	RCT	1989 - 1992	600-1250	indomethacin: 27.7±2 Placebo:28±2.2	NA	505	138		0-11	73
										12-35	27
										36-59	2
										60-95	10
										96-119	6
Perlman et al. (1986)[26]	USA	prospective observational	1982 - 1985	500-700	NA	NA	55	34	62	0-18	21
										19-72	7
										>72	6
				701-1000			366	39		0-18	7
										19-72	30
										>72	2
				1001-15000				52		0-18	6
										19-72	44
										>72	2
Amato et al. (1989)[27]	Switzerland	prospective observational	1983 - 1985	<34 <1501	IVH:28.2 ± 2.6, noIVH 31.3 ± 2.4	NA	50	15	1	0-24	2
										24-48	3
										48-72	9
										72-96	1
Duppré et al. (2015)[28]	Germany	retrospective observational	2010 - 2013	<1500 23-35	NoIVH:29.5 ± 2.4 IVH:27.4± 2.4	NA	250	55	25	0-24	11
										24-48	19
										48-72	14
										>72	11
	Japan	NA		<27	NA	NA	216	NA	55	0-24	7

© 2024 Nagy Zs et al. *JAMA Pediatrics.*

Naoshi et al. (2021) The 73rd Annual Congress of the Japan[29]			2009 - 2020							24-48	25
										48-72	13
										72-96	6
										96-120	4
Perlman et al. (1983)[30]	USA	NA	1982	<1500	30±3	NA	57	35	NA	0-12	7
										0-24	4
										24-48	18
										48-72	3
										72-96	1
										96-168	2
Schreiner et al. (2021)[31]	Austria	retrospective observational	2010 - 2018	<32	NA	NoIVH: 30.0 [28.3; 31.3] IVH: 28.9 [27.0;30.4]	536	80	16	0-24	7
										24-48	23
										48-72	15
										72-168	14
										>168	21
Salafia et al. (1995)[32]	USA	retrospective observational	1988 - 1993	<32	Early IVH: 26.5±2. Late IVH: 28.4 ± 2.3	NA	406	65	23	<72	44
										72-432	21
Zanardo et al. (2008)[33]	Italy	prospective observational	1999 - 2004	<32	NoIVH: 27.2±2.4, IVH: 25.5±2.4	NA	287	33	3	<72	19
										>72	14
Kalani et al. (2016)[34]	Iran	retrospective observational	2013 - 2014	<32 <1500	30.27±1.7	NA	93	14	NA	0-D3	5
										D3-D7	1
										D3-D14	6
										D7-D14	2

© 2024 Nagy Zs et al. *JAMA Pediatrics.*

Najib et al. (2018)[35]	Iran	prospective observational	2014 - 2015	26-32	NA	NA	68	19	NA	0-D3	11
										D7-D10	8
Florio et al. (2006)[36]	Italy	prospective observational	2002 - 2003	≤32	IVH:28.12± 1.5<br>noIVH:28.3±9 0.05	NA	53	11	0	0-D1	0
										D1-D10	11
*Galderisi et al. (2019)[37]	Italy	secondary analysis of RCT	2015 - 2016	≤32	NA	group1:<br>31[28, 32]<br>group2:<br>28[26, 28]<br>group3:<br>30[28,31]<br>group4: 28[28, 32]	50	11	2	0-49	0
										49-71	4
										95-119	3
										119-143	1
										49-168	1
										71-95	2
Osborn et al. (2003)[38]	Australia	prospective observational	1998 - 1999	<30	NoIVH:27.0±1.7, EarlyIVH:26.6 ±1.6	NA	128	38	17	0-3	19
										4-D28	19
Paradisis et al. (2009)[39]	Australia	RCT	2003 - 2006	<30	Milrinon:26±1.1<br>placebo:26±1.3	NA	93	25	NA	0-6	10
										6-D28	15
Szymonovicz et al. (1984)[40]	Australia	prospective observational	NA	24-32	27.0±4	NA	50	30	NA	0-12	15
										0-24	2
										12-24	2
										24-96	11

© 2024 Nagy Zs et al. *JAMA Pediatrics.*

Tanaka et al. (2022)[41]	Japan	prospective observational	2015-2020	<28 <1500	NA	NoIVH:26.0 [24.0,26.0] G1-2:25.0 [24.0,26.5] sIVH:24.0 [23.0,26.5]	64	13	NA	0-2	3
										2-24	0
										25-36	2
										37-48	3
										49-60	4
										73-84	1
										84-D28	0
Van Bel et al (2002)[42]	The Netherlands	prospective observational	NA	25-32	noIVH:29.1±1.6, MildIVH:28.2±1.7, sIVH:27.4±1.0	NA	83	23	5	0-12	8
										13-24	2
										25-48	7
										49-72	3
										73-168	3
Weindling et al (1985)[43]	UK	prospective observational	1982-1983	<32	NA	NA	82	34	3	0-6	4
										6-12	3
										12-18	2
										30-48	4
										0-discharge	21
Iyer et al. (2015)[44]	Sweden	cross-sectional	2005-2007	22-28	NA	NA	25	14	5	0-24	5
										0-48	3
										24-48	1
										24-72	5
*Himanshu et al (2019)[45]	Australia	prospective observational	2012-2016	<32	27±1	NA	51	11	N.A	0-6	1
										6-168	10
Thanhaeuser et al. (2018)[46]	Austria	prospective		<1000 23-33	NA		128	33	15	0-5,7	15
										0-D30	16

© 2024 Nagy Zs et al. *JAMA Pediatrics.*

		observational	2013-2015			noIVH:25[6], bleeding group:25[2]				0-discharge	2
Zanelli et al. (2021)[47]	USA	retrospective observational	2009-2018	<32 <1500	27	NA	638	279	84	0-48	34
										48-D10	37
										0-D10	208
Thorburn et al. (1982)[48]	UK	N.A.	1979	23-32	NA	no IVH:31 IVH:28	95	36	13	0-24	12
										24-48	4
										48-72	8
										72-96	5
										144-168	1
										0-discharge	6
Bates et al. (2015)[49]	UK	retrospective cochort	2008-2012	<28	IVH: 25.1 ±1.3 NoIVH:25.7±1.0	NA	108	46	22	0-12	8
										0-168	38
Yang et al. (1990)[50]	Taiwan	prospective observational	N.A.	500-1500	29.30±2.55	NA	83	45	15	0-24	22
										25-72	13
										73-120	7
										120-168	3
Bada et al. (1989)[51]	USA	prospective,random selection, double-blind clinical trial	1989	<1500 <31	28±2.4	NA	145	68	25	0-1	26
										1-336	42
Lampe et al. (2020)[52]	Germany	retrospective observational	2006-2016	23-30	control: 26.7±2. group1:	NA	254	136	56	0-24	16
										24-48	20
										48-72	40

© 2024 Nagy Zs et al. *JAMA Pediatrics.*

					26.3±2.0					72-96	20
										96-120	11
										120-336	29
Rumack et al. (1985)[53]	USA	prospective observational	N.A.	25-32	NA	NA	49	21	7	0-3	1
										0-4	4
										0-7	1
										0-18	1
										0-24	1
										24-48	7
										48-72	4
										120-144	1
										168-192	1
*Vesoulis et al. (2019)[54]	USA	prospective observational	2012 - 2017	<30	NosevereIVH: 25.2±1.9 SevereIVH25. 1±1.5	NA	157	61	29	0-24	17
										25-48	20
										49-72	12
										73-168	12
*Vesoulis et al. (2020)[55]	USA	prospective observational	2012 - 2019	<32	26.3±2.0	NA	185	54	16	0-30	13
										0-72	18
										72-168	8
										72-240	7
										240-270	8
Evans et al. (1996)[56]	Australia	retrospective observational	1992 - 1993	<1500 24-33	27.3	NA	141	36	10	0-31	8
										31-D28	19
										0-D28	9
*Sortica da Costa et al. (2018)[57]	UK	prospective	2013 - 2015	<32	NA	NA	56	18	9	individual data in hours and minutes	

© 2024 Nagy Zs et al. *JAMA Pediatrics.*

		observational									
Paradisis et al. (2006)[58]	Australia	prospective observational	2002-2003	<28	NA	NA	29	8	3	0-3	4
										3-30	3
										30-119	1
Hoffman et al. (2018)[59]	USA	prospective observational	2013-2016	24-29	noIVH: 26.5±1.6<br>sIVH:25.3±1.7	NA	61	N.A.	7	0-48	3
										0-168	4
*Juul et al. (2020)[60]	USA	RCT	2013-2016	24-28	Group1:<br>26.0±1.2, Group2: 25.8±1.1	NA	906	344	107	PENUT Trial<br>individual data in hours and minutes	
*Martini et al. (2022)[61]	Italy	prospective observational	2018-2022	<32	NA	29[26.1–31]	77	16	5	12-36	4
										9-21	1
										30-50	1
										8-32	1
										48-72	1
										12-24	1
										46-70	1
										10-22	1
										7-19	1
										26-50	1
										8-24	1
										6-30	1
										41-65	1
Rojas et al. (2021)[62]	Lima, Peru	retrospective cohort		<28	NA	NA	72	42	N.A.	0-24	10
										25-48	8

© 2024 Nagy Zs et al. *JAMA Pediatrics.*

			2013 - 2017							49-72	4
										73-96	6
										97-120	5
										120-144	1
										145-168	1
										169-192	2
										193-384	5
Ment et al. (1985)[63]	USA	RCT	1983 - 1985	600-1250	placebo:28.5± 2.20, Indo:28.7±1.92	NA	66	38	N.A.	0-6	18
										6-18	2
										18-30	6
										30-42	6
										42-54	1
										54-96	4
										67-120	1
Skubisz et al. (2024)[64]	the Netherlands	retrospective case-series analysis from a consecutive cohort	2015 - 2019	<32	N.A.	RBC transfusion before IVH: 26[4]<br>RBC transfusion within 48h after IVH: 26[4]<br>without RBC transfusion in first week after birth: 28[3]	N.A.	139	N.A.	0-6	34
										6-12	19
										12-24	19
										24-48	28
										48-72	14
										72-96	7
										96-120	7
										120-144	5
										168-	6
Jiang et al. (2023)[65]	China	prospective		≤32<br>≤1500 g	28.95 ± 2.33	N.A.	92	49	8	0-24	25
										24-72	19

		observational	2020 - 2023						72-168	5
Shan et al. (2023)[66]	China	retrospective observational	2019 - 2022	<1000 g	IVH: 27.4±1.5 NoIVH: 27.8±1.5	N.A.	238	82	28	0-72	68
									72-168	8
									168-	6
June et al. (2021)[67]	US	prospective observational	2018	<1250 g	25.9(22–30) 809(380–1,220)		99	36	21	0-24	5
									0-168	31

*Individual data from the authors; IVH any grade Intraventricular hemorrhage; sIVH severe Intraventricular hemorrhage; G1 grade 1; G2 grade 2; G3 grade 3; G4, grade 4; RDS Respiratory Distress Syndrome; No IVH without any grade Intraventricular hemorrhage; h hours; HOL hours of life; DOL days of life; D days; IQR interquartile range; NA not reported; RCT randomized; UCM umbilical cord milking; ICC immediate cord clamping; RBC red blood cell

© 2024 Nagy Zs et al. *JAMA Pediatrics.*

eTable 2. b. Baseline characteristics of the included neonatal studies

Study	Early mortality rate (%)	Total mortality rate (%)	Outborn rate (%)	Antenatal steroid prophylaxis rate (%)	DCC rate (%)	Magnesium administration rate (%)
Alderliesten et al. (2013)[4]	NA	NA	NA	NA	NA	NA
*Noori et al. (2014)[5]	NA	22	NA	86	NA	NA
Sarkar et al. (2005)[6]	0	6	NA	90	NA	NA
Krediet et al. (2006)[7]	NA	NA	NA	73	NA	NA
Cimatti et al. (2020)[8]	4	NA	NA	NA	NA	NA
*Deshpande et al. (2020)[9]	2	25	2	90	56	76
El-Dib et al. (2022)[10]	NA	NA	NA	NA	NA	NA
Babnik et al. (2006)[11]	0	10	0	87	NA	NA
Bada et al. (1984)[12]	NA	NA	NA	NA	NA	14,2
Chalak et al. (2011)[13]	NA	NA	0	100	NA	NA
Dani et al. (2005)[14]	NA	NA	NA	74	NA	NA
Dolfin et al. (1983)[15]	3	18	0	NA	NA	NA
Farag et al. (2022)[16]	NA	NA	NA	53	NA	NA
Ikeda et al. (2014)[17]	1	NA	0	12	NA	NA
Ishiguro et al. (2014)[18]	NA	NA	NA	64	NA	NA
Katheria et al. (2014)[19]	NA	3	0	100	NA	78
Katheria et al. (2018)[20]	1	NA	NA	95	0	84
Meek et al. (1998)[21]	0	8	NA	NA	NA	NA
Kluckow et al. (2000)[22]	2	18	9	87	NA	NA
McDonald et al. (1984)[23]	0	14	0	NA	NA	NA
Ment et al. (1984)[24]	0	29	NA	NA	NA	NA
Ment et al. (1994)[25]	7	17	17	30	NA	NA
Perlman et al. (1986)[26]	0	14	NA	NA	NA	NA
Amato et al. (1989)[27]	NA	NA	0	NA	NA	NA
Duppré et al. (2015)[28]	0	10	0	84	NA	NA
Naoshi et al. (2021)[29]	NA	NA	NA	NA	NA	NA
Perlman et al. (1983)[30]	NA	NA	43	NA	NA	NA
Schreiner et al. (2021)[31]	NA	NA	NA	93	NA	NA
Salafia et al. (1995)[32]	NA	NA	NA	34	NA	18
Zanardo et al. (2008)[33]	0	7	NA	80	NA	NA

© 2024 Nagy Zs et al. *JAMA Pediatrics.*

Kalani et al. (2016)[34]	NA	NA	NA	NA	NA	NA
Najib et al. (2018)[35]	NA	NA	0	60	NA	NA
Florio et al. (2006)[36]	NA	NA	NA	17	NA	NA
Galderisi et al. (2019)[37]	NA	NA	NA	NA	NA	NA
Osborn et al. (2003)[38]	NA	NA	NA	92	NA	NA
Paradisis et al. (2009)[39]	NA	NA	0	96	NA	NA
Szymonovicz et al. (1984)[40]	11	NA	7	NA	NA	NA
Tanaka et al. (2022)[41]	NA	8	0	42	NA	NA
Van Bel et al. (2002)[42]	NA	NA	NA	NA	NA	NA
Weindling et al. (1985)[43]	NA	NA	21	NA	NA	NA
Iyer et al. (2015)[44]	NA	NA	NA	NA	NA	NA
Himanshu et al (2019)[45]	NA	NA	NA	98	0	NA
Thanhaeuser et al. (2018)[46]	7	14	NA	94	NA	NA
Zanelli et al. (2021)[47]	NA	NA	17	NA	NA	NA
Thorburn et al. (1982)[48]	0	29	58	11	NA	NA
Bates et al. (2015)[49]	9	10	20	91	NA	NA
Yang et al. (1990)[50]	10	26	0	NA	NA	NA
Bada et al. (1989)[51]	NA	NA	0	46	NA	NA
Lampe et al. (2020)[52]	NA	NA	0	NA	NA	NA
Rumack et al. (1985)[53]	NA	NA	NA	NA	NA	NA
Vesoulis et al. (2019)[54]	NA	NA	NA	75	NA	NA
Vesoulis et al. (2020)[55]	11	21	NA	75	28	NA
Evans et al. (1996)[56]	2	NA	NA	72	NA	NA
*Sortica da Costa et al. (2018)[57]	0	7	12	98	NA	27
Paradisis et al. (2006)[58]	NA	NA	0	100	NA	NA
Hoffman et al. (2018)[59]	NA	NA	NA	NA	NA	NA
*Juul et al. (2020)[60]	6	NA	NA	90	47	79
Martini et al. (2022)[61]	NA	NA	NA	NA	NA	NA
Rojas et al. (2021)[62]	NA	NA	NA	NA	NA	NA
Ment et al. (1985)[63]	NA	NA	NA	NA	NA	NA
Skubisz et al. (2024)[64]	NA	NA	9,3	92	NA	NA
Jiang et al. (2023)[65]	2	9	0	60	NA	NA
Shan et al. (2023)[66]	NA	NA	NA	NA	NA	NA
June et al. (2021)[67]	NA	NA	4	NA	NA	NA

*Individual data from the authors, DCC delayed cord clamping

© 2024 Nagy Zs et al. *JAMA Pediatrics.*

eTable 3. IVH rate among GA<28 and GA<32 weeks infants by postnatal age after 2007

Hours of life (HOL)	Subgroup	Proportion	95%CI
0-6	GA<28	0.1	0.02-0.34
	GA<32	0.04	0.01-0.13
0-12	GA<28	0.11	0.04-0.31
	GA<32	0.07	0,02-0,25
0-24	GA<28	0.15	0.07-0.31
	GA<32	0.12	0.05-0.27
0-48	GA<28	0.29	0.15-0.48
	GA<32	0.16	0.08-0.30
0-72	GA<28	0.30	0.19-0.45
	GA<32	0.24	0.13-0.40

HOL hours of life; CI Confidence Intervals; GA Gestational age

© 2024 Nagy Zs et al. *JAMA Pediatrics.*

eTable 4. Risk of bias assessment using Quality in Prognostic Studies 2 (QUIPS2) tool

Risk of bias domains

Study	D1	D2	D3	D4	D5	D6	Overall
Thanhaeuser_2018	+	-	+	+	+	X	X
Alderliesten_2013	+	+	+	+	+	+	+
Noori_2014	+	+	+	+	+	+	+
Sarkar_2005	-	+	+	+	+	+	-
Krodiet_1996	-	-	+	+	-	+	X
Cimatti_2020	+	+	+	+	+	+	+
Deshpande_2020	+	+	+	+	+	+	+
Martini_2022	+	+	+	+	+	+	+
El_Dib_2022	+	?	+	+	+	+	+
Babnik_2006	+	+	+	+	-	+	-
Bada_1984	-	+	+	+	-	+	X
Chalak_2011	-	+	+	+	+	+	-
Dani_2005	-	+	+	+	+	+	-
Dolfin_1983	+	+	+	+	+	+	+
Farag_2022	+	+	+	+	+	+	+
Ikeda_2014	+	-	+	+	+	+	-
Ishiguro_2014	+	+	+	+	+	+	+
Katheria_2014	-	+	+	+	+	+	-
Katheria_2018	+	+	+	+	+	+	+
Meek_1998	X	+	+	+	-	+	X
Kluckow_2000	-	-	+	-	+	+	X
McDonald_1984	+	+	+	+	+	+	+
Ment_1984	+	+	+	+	+	+	+
Ment_1994	-	-	+	-	+	+	X
Osborn_2003	-	-	+	-	+	+	X
Paradisis_2009	-	+	+	-	+	+	X
Ment_1995	-	X	+	-	-	+	X
Himanshu_2019	+	+	+	+	+	+	+
Amato_1989	-	-	+	+	+	+	X
Duppré_2015	+	?	+	-	+	+	-
Naoshi_2021	X	?	-	-	X	+	X
Perlman_1983	-	-	+	-	-	+	X
Perlman_1986	+	+	+	+	-	+	X
Schreiner_2021	X	?	+	-	+	+	X
Salafia_1995	-	?	+	-	-	+	X
Zanardo_2008	+	+	+	-	+	-	X
Kalani_2016	X	?	+	-	-	-	X
Najib_2018	X	-	+	-	+	+	X
Florio_2006	-	+	+	+	+	+	-
Galderisi_2019	-	?	+	+	+	+	-
Hoffman_2018	-	+	+	+	-	+	X
Szymonovicz_1984	-	+	+	-	-	+	X
Tanaka_2022	X	+	+	+	+	+	X
Van Bel_2002	-	+	+	+	-	+	X
Weindling_1985	+	+	+	-	+	+	-
Iyer_2015	+	-	+	+	-	+	X
Zanelli_2021	+	?	+	-	+	+	-
Thorburn_1982	-	+	+	+	+	+	-
Bates_2015	X	?	+	-	+	+	X
Rojas_2021	-	?	+	-	+	+	-
Yang_1990	+	+	+	+	-	+	-
Bada_1989	-	-	+	-	+	+	X
Lampe_2020	+	?	+	+	+	+	+
Rumack_1985	X	-	-	+	-	+	X
Vesoulis_2019	-	+	+	+	+	+	-
Vesoulis_2020	+	+	+	+	+	+	+
Evans_1996	-	?	+	+	+	+	-
Da Costa_2018	+	+	+	+	+	+	+
Paradisis_2006	-	+	+	-	+	+	X
Skubisz_2024	+	+	+	+	+	+	+
Jiang_2023	+	-	+	+	+	+	-
Shan_2023	X	+	+	+	+	+	X
June_2021	+	+	+	-	+	+	-

Domains:
D1: Bias due to participation.
D2: Bias due to attrition.
D3: Bias due to prognostic factor measurement.
D4: Bias due to outcome measurement.
D5: Bias due to confounding.
D6: Bias in statistical analysis and reporting.

Judgement
X High
- Moderate
+ Low
? No information

© 2024 Nagy Zs et al. *JAMA Pediatrics.*

eFigure 1. Any grade IVH proportion by GA limits in GA<28, GA<32 and non-categorized studies before 2007

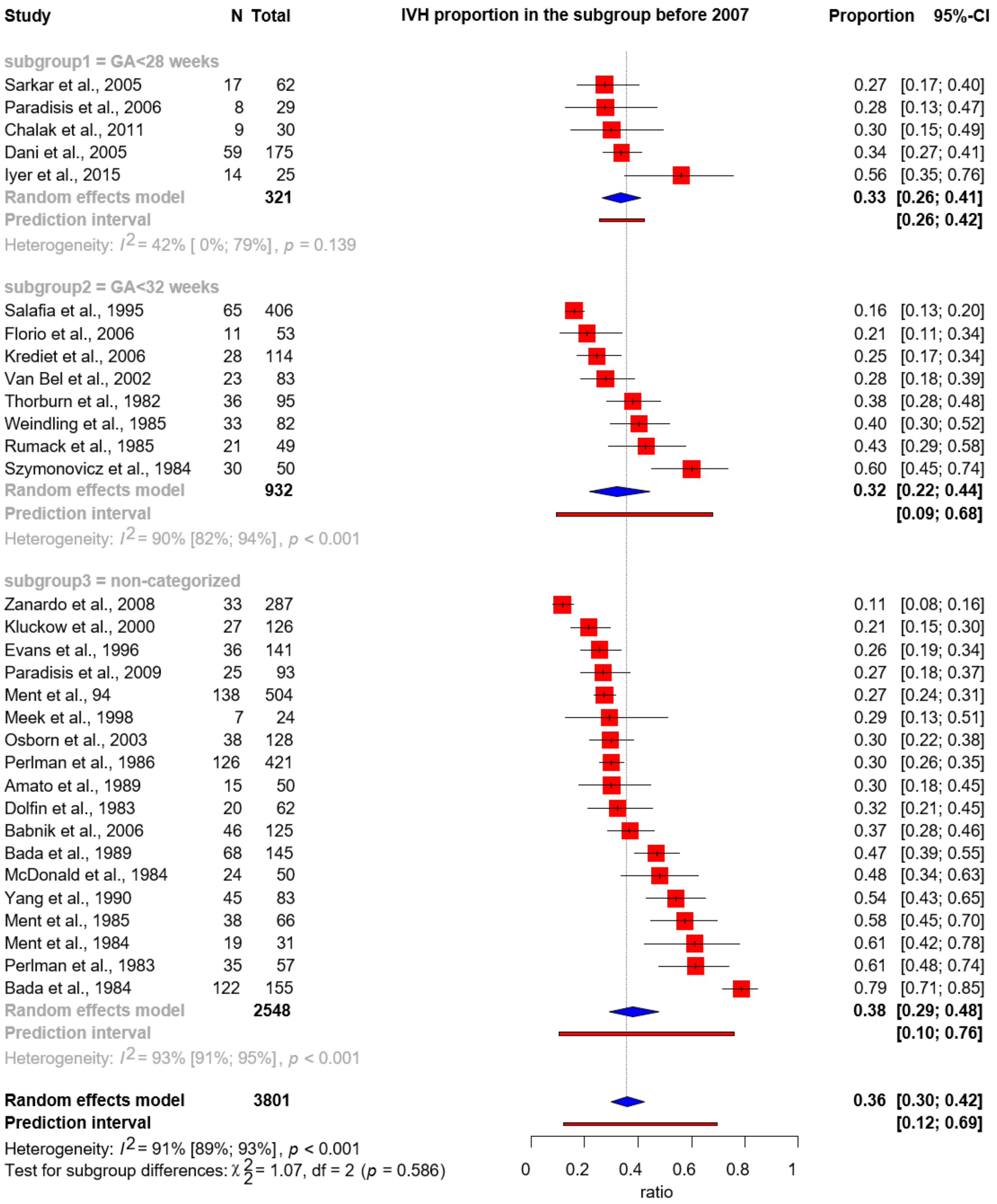

IVH: Intraventricular hemorrhage; CI: Confidence Intervals

© 2024 Nagy Zs et al. *JAMA Pediatrics.*

eFigure 2. Any grade IVH proportion by GA limits in GA<28, GA<32, and non-categorized studies after 2007

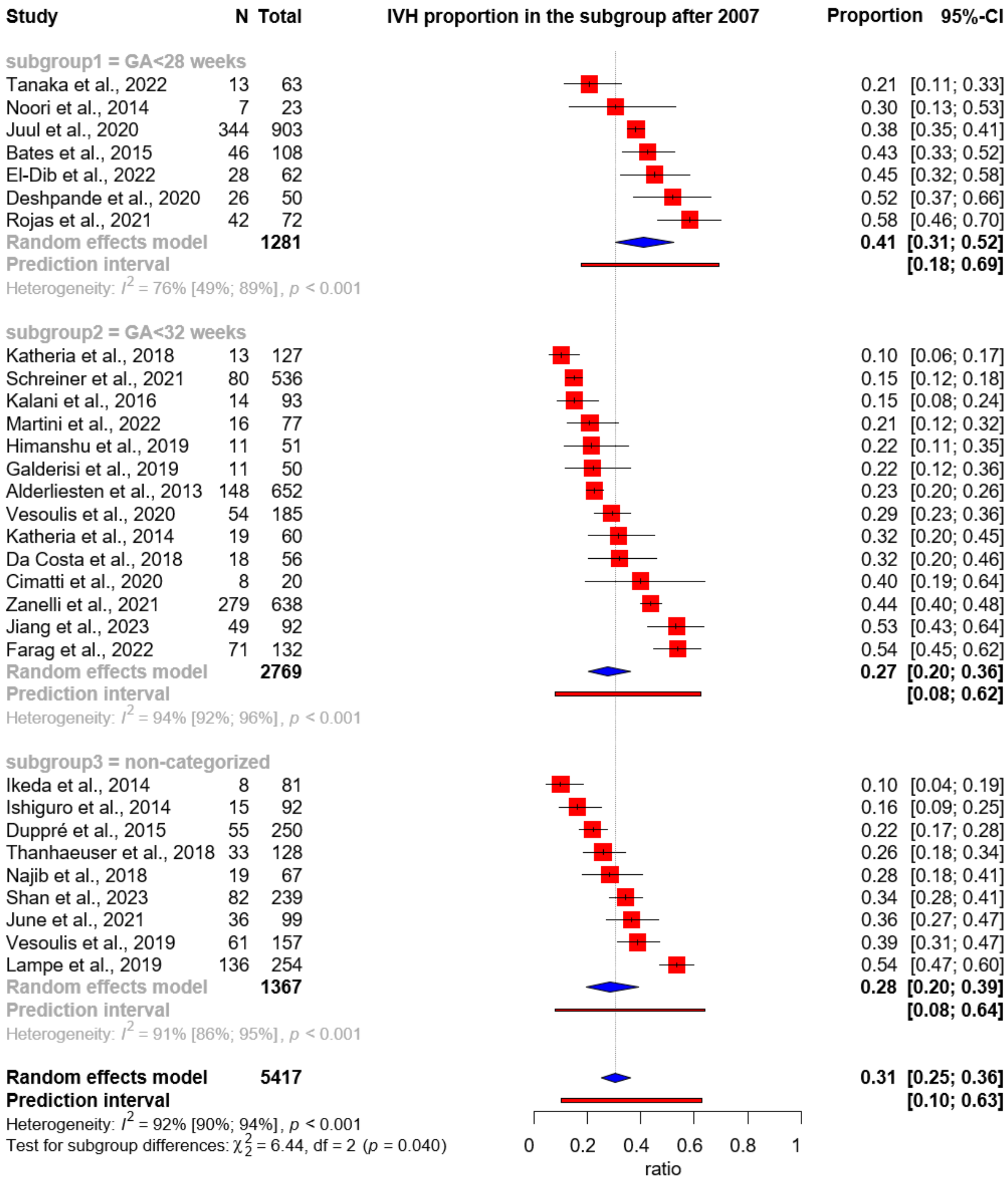

IVH: Intraventricular hemorrhage; CI: Confidence Intervals; GA: gestational age

© 2024 Nagy Zs et al. *JAMA Pediatrics.*

eFigure 3. Proportion of severe IVH by GA limits in GA<28, GA<32, non-categorized subgroups before 2007

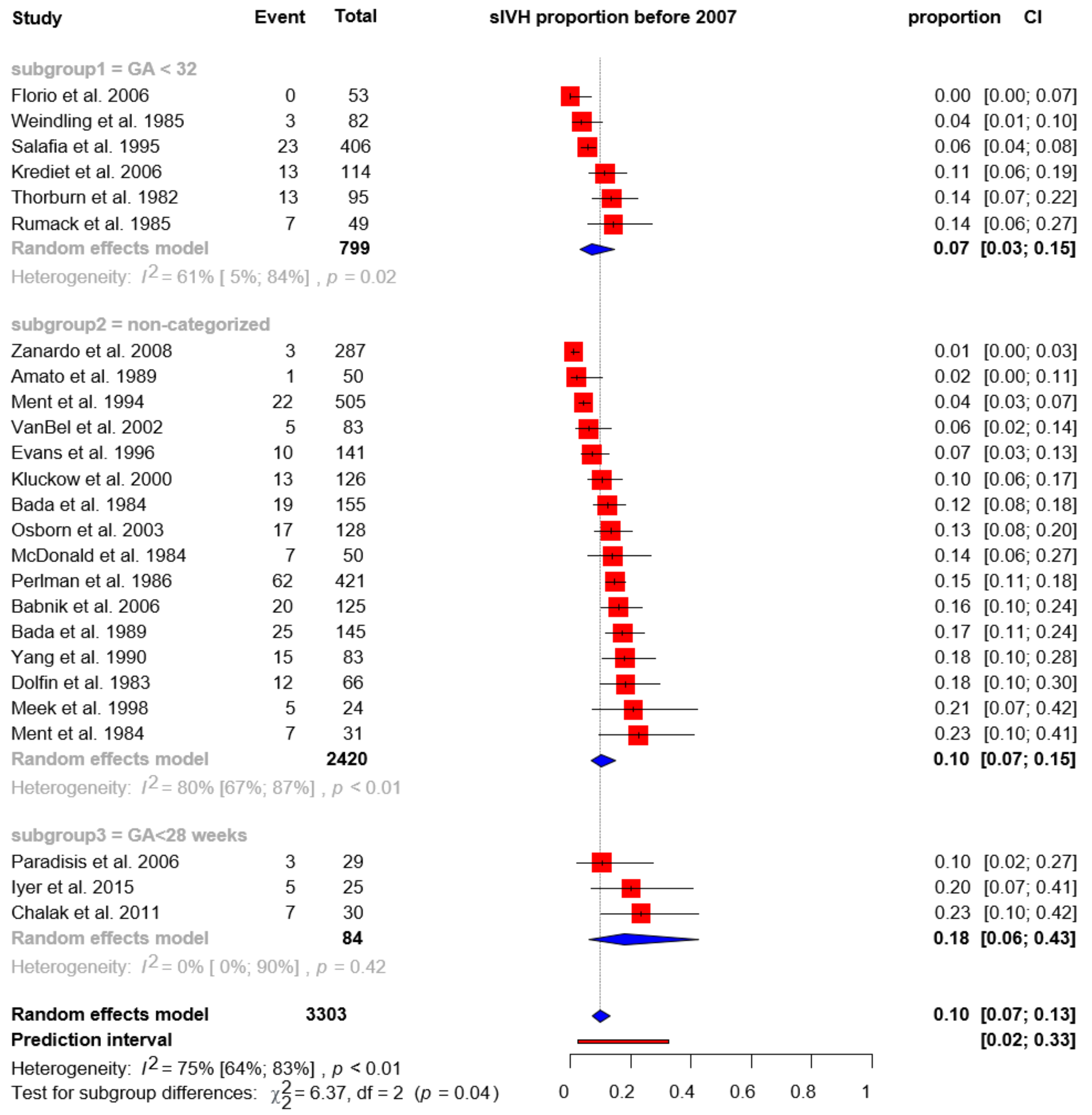

IVH: Intraventricular hemorrhage; CI: Confidence Intervals; GA: gestational age

© 2024 Nagy Zs et al. *JAMA Pediatrics.*

eFigure 4. Proportion of severe IVH by GA limits in GA<28, GA<32, and non-categorized studies after 2007

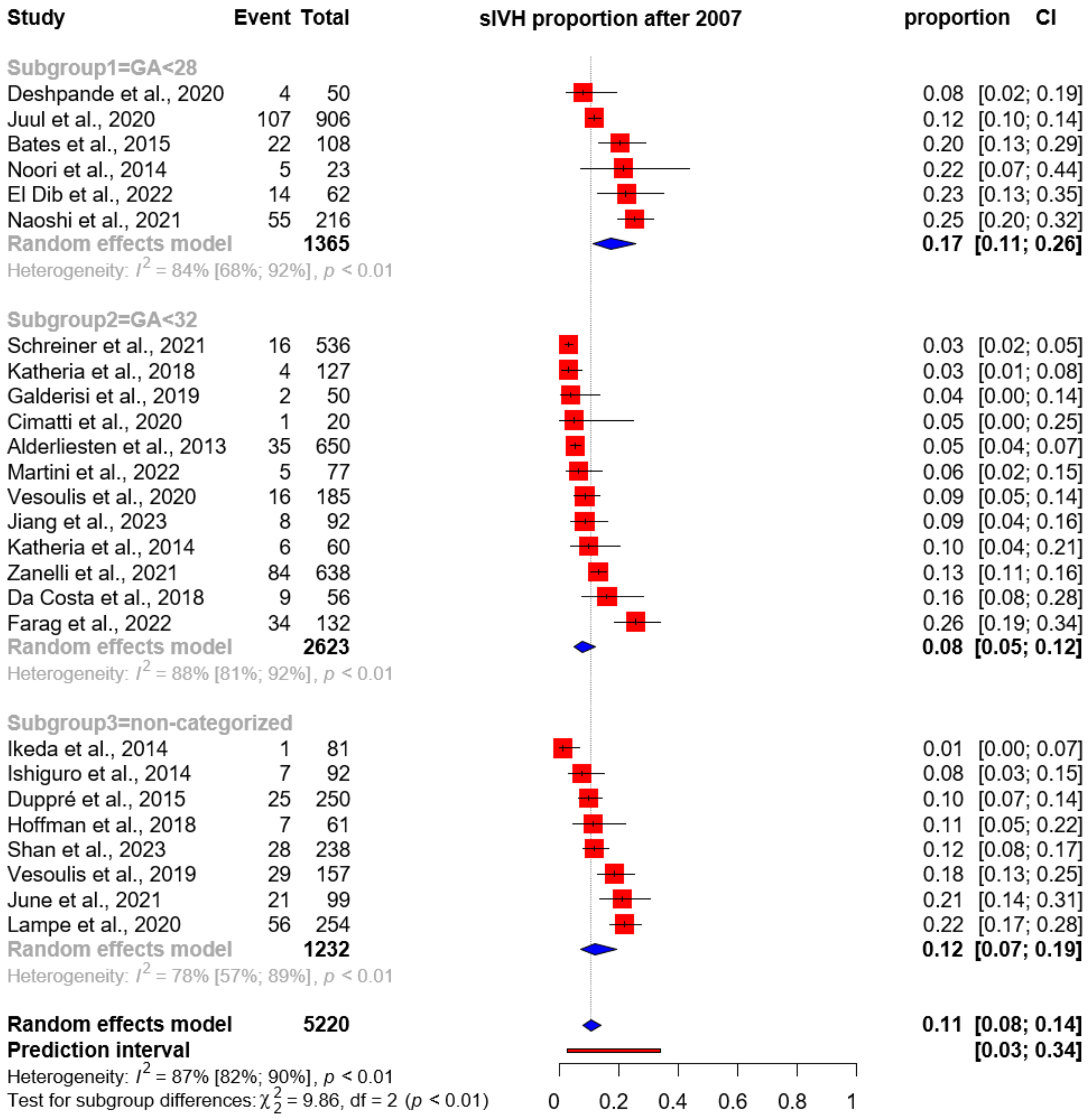

sIVH: severe intraventricular hemorrhage; CI: Confidence Intervals; GA: gestational age

© 2024 Nagy Zs et al. *JAMA Pediatrics.*

eFigure 5. IVH rate up to 6 HOL of all preterm infants in the subgroups before and after 2007

study	logit	se	proportion	ci	w.random
**subgroup1 = before 2007**					
Florio et al., 2006	-5.2933	1.9474	0.00	[0.00; 0.19]	1.5%
Dolfin et al., 1983	-2.6744	0.5171	0.06	[0.02; 0.16]	8.2%
Weindling et al., 1985	-2.6744	0.5171	0.06	[0.02; 0.16]	8.2%
Kluckow et al., 2000	-2.5654	0.3454	0.07	[0.04; 0.13]	10.2%
Sarkar et al., 2005	-2.4342	0.4669	0.08	[0.03; 0.18]	8.8%
Paradisis et al., 2009	-2.1165	0.3346	0.11	[0.06; 0.19]	10.3%
Osborn et al., 2003	-1.7469	0.2486	0.15	[0.10; 0.22]	11.2%
Rumack et al., 1985	-1.6339	0.3864	0.16	[0.08; 0.29]	9.7%
Dani et al., 2005	-1.6164	0.2033	0.17	[0.12; 0.23]	11.6%
Ment et al., 1984	-1.0559	0.4105	0.26	[0.13; 0.44]	9.4%
Ment et al., 1985	-0.9810	0.2763	0.27	[0.18; 0.39]	10.9%
**Random effects model (HK)**			**0.13**	**[0.08; 0.19]**	**100.0%**
Heterogeneity: $I^2 = 66\%$ [36%; 82%], $\tau^2 = 0.2547$, $p < 0.01$					
**subgroup2 = after 2007**					
Cimatti et al., 2020	-5.2933	3.1702	0.00	[0.00; 0.72]	0.9%
Galderisi et al., 2019	-5.2933	2.0050	0.00	[0.00; 0.20]	2.2%
Ikeda et al., 2014	-5.2933	1.5753	0.00	[0.00; 0.10]	3.3%
Martini et al., 2022	-5.2933	1.6157	0.00	[0.00; 0.11]	3.2%
Katheria et al., 2014	-4.0755	1.0048	0.02	[0.00; 0.11]	6.5%
Himanshu et al., 2019	-3.9124	1.0096	0.02	[0.00; 0.13]	6.5%
Ishiguro et al., 2014	-3.3903	0.5866	0.03	[0.01; 0.10]	11.6%
Noori et al., 2014	-3.0905	1.0214	0.04	[0.01; 0.25]	6.4%
Tanaka et al., 2022	-3.0123	0.5914	0.05	[0.02; 0.14]	11.5%
Katheria et al., 2018	-2.6827	0.4056	0.06	[0.03; 0.13]	14.6%
El-Dib et al., 2022	-1.3769	0.9111	0.20	[0.04; 0.60]	7.4%
Da Costa et al., 2018	-1.3664	0.3650	0.20	[0.11; 0.34]	15.4%
Juul et al., 2020	-1.3517	0.6545	0.21	[0.07; 0.48]	10.5%
**Random effects model (HK)**			**0.05**	**[0.02; 0.11]**	**100.0%**
Heterogeneity: $I^2 = 61\%$ [29%; 79%], $\tau^2 = 0.8775$, $p < 0.01$					
Test for subgroup differences: $\chi^2_1 = 5.39$, df = 1 ($p = 0.02$)					

IVH probability 0-6 HOL in all preterm neonates

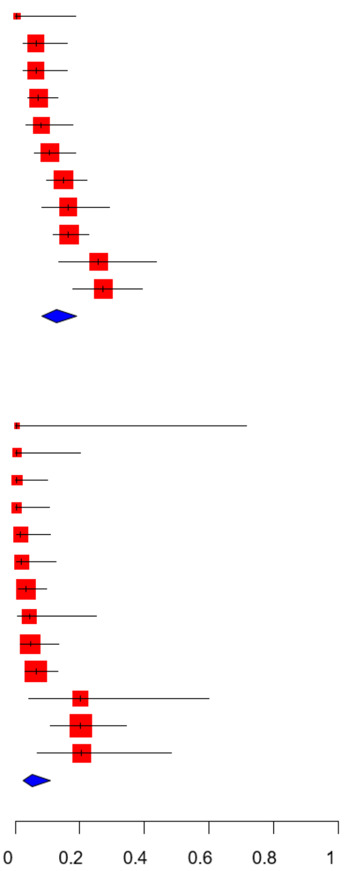

IVH: intraventricular haemorrhage; HOL: hours of life

© 2024 Nagy Zs et al. *JAMA Pediatrics.*

eFigure 6. IVH rate up to 12 HOL of all preterm infants in the subgroups before and after 2007

study	logit	se	IVH prob. 0-12 HOL in all preterm neonates	Proportion		ci w.random
**subgroup1 = before 2007**						
Florio et al., 2006	-5.2933	1.9474		0.00	[0.00; 0.19]	0.7%
Sarkar et al., 2005	-2.4342	0.4669		0.08	[0.03; 0.18]	7.3%
Kluckow et al., 2000	-2.3471	0.3150		0.09	[0.05; 0.15]	10.6%
Van Bel et al., 2002	-2.2379	0.3720		0.10	[0.05; 0.18]	9.2%
Dolfin et al., 1983	-2.2333	0.4289		0.10	[0.04; 0.20]	8.0%
Weindling et al., 1985	-2.0615	0.4014		0.11	[0.05; 0.22]	8.6%
Perlman et al., 1983	-1.9662	0.4038		0.12	[0.06; 0.24]	8.5%
Ment et al., 1994	-1.7760	0.1268		0.14	[0.12; 0.18]	15.3%
Krediet et al., 2006	-1.6822	0.2587		0.16	[0.10; 0.24]	12.0%
Rumack et al., 1985	-1.6339	0.3864		0.16	[0.08; 0.29]	8.9%
Szymonovicz et al., 1984	-0.6843	0.3029		0.34	[0.22; 0.48]	10.9%
**Random effects model (HK)**				**0.13**	**[0.09; 0.18]**	**100.0%**
Heterogeneity: $I^2 = 60\%$ [23%; 79%], $\tau^2 = 0.1659$, $p < 0.01$						
**subgroup2 = after 2007**						
Cimatti et al., 2020	-5.2933	3.1702		0.00	[0.00; 0.72]	0.5%
Galderisi et al., 2019	-5.2933	2.0050		0.00	[0.00; 0.20]	1.2%
Martini et al., 2022	-5.2933	1.6157		0.00	[0.00; 0.11]	1.8%
Noori et al., 2014	-3.0910	1.0225		0.04	[0.01; 0.25]	4.2%
Katheria et al., 2018	-2.6827	0.4056		0.06	[0.03; 0.13]	15.3%
Bates et al., 2015	-2.5257	0.3674		0.07	[0.04; 0.14]	16.8%
Alderliesten et al., 2013	-2.0826	0.1309		0.11	[0.09; 0.14]	27.5%
Da Costa et al., 2018	-1.3664	0.3650		0.20	[0.11; 0.34]	16.9%
Juul et al., 2020	-1.3517	0.7721		0.21	[0.05; 0.54]	6.7%
El-Dib et al., 2022	-1.3117	0.6275		0.21	[0.07; 0.48]	9.1%
**Random effects model (HK)**				**0.10**	**[0.06; 0.18]**	**100.0%**
Heterogeneity: $I^2 = 51\%$ [0%; 76%], $\tau^2 = 0.1935$, $p = 0.03$						
Test for subgroup differences: $\chi^2_1 = 0.75$, df = 1 ($p = 0.39$)						

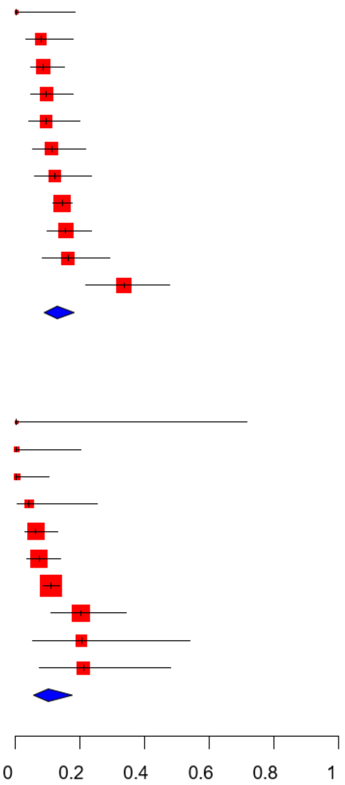

IVH: intraventricular haemorrhage; HOL: hours of life

© 2024 Nagy Zs et al. *JAMA Pediatrics.*

eFigure 7. IVH rate up to 24 HOL of all preterm infants in the subgroups before and after 2007

study	logit	se	proportion	CI	w.random
**subgroup1 = before 2007**					
Florio et al., 2006	-5.2933	1.9474	0.00	[0.00; 0.19]	1.2%
Amato et al., 1989	-3.1781	0.7214	0.04	[0.01; 0.15]	4.4%
Sarkar et al., 2005	-2.4342	0.4669	0.08	[0.03; 0.18]	5.7%
Meek et al., 1998	-2.3983	0.7386	0.08	[0.02; 0.28]	4.3%
Kluckow et al., 2000	-2.0019	0.2757	0.12	[0.07; 0.19]	6.7%
Van Bel et al., 2002	-1.9877	0.3369	0.12	[0.07; 0.21]	6.4%
Thorburn et al., 1982	-1.8592	0.3104	0.13	[0.08; 0.22]	6.5%
Weindling et al., 1985	-1.7728	0.3601	0.15	[0.08; 0.26]	6.3%
Rumack et al., 1985	-1.6339	0.3864	0.16	[0.08; 0.29]	6.1%
Dolfin et al., 1983	-1.5238	0.3329	0.18	[0.10; 0.29]	6.4%
Perlman et al., 1983	-1.4306	0.3358	0.19	[0.11; 0.32]	6.4%
McDonald et al., 1984	-1.2657	0.3415	0.22	[0.13; 0.36]	6.4%
Iyer et al., 2015	-1.0748	0.5028	0.25	[0.11; 0.48]	5.5%
Yang et al., 1990	-1.0196	0.2484	0.27	[0.18; 0.37]	6.8%
Dani et al., 2005	-0.8886	0.1666	0.29	[0.23; 0.36]	7.1%
Szymonovicz et al., 1984	-0.4895	0.2912	0.38	[0.26; 0.52]	6.6%
Bada et al., 1984	0.1942	0.1615	0.55	[0.47; 0.62]	7.1%
**Random effects model (HK)**			**0.18**	**[0.13; 0.26]**	**100.0%**
Heterogeneity: $I^2 = 87\%$ [81%; 91%], $\tau^2 = 0.5006$, $p < 0.01$					
**subgroup2 = after 2007**					
Galderisi et al., 2019	-5.2933	2.0050	0.00	[0.00; 0.20]	1.1%
Ikeda et al., 2014	-4.3858	1.0125	0.01	[0.00; 0.08]	2.8%
Schreiner et al., 2021	-4.3220	0.3790	0.01	[0.01; 0.03]	5.6%
Noori et al., 2014	-3.0910	1.0225	0.04	[0.01; 0.25]	2.8%
Duppre et al., 2015	-3.0786	0.3091	0.04	[0.02; 0.08]	5.9%
Tanaka et al., 2022	-3.0123	0.5914	0.05	[0.02; 0.14]	4.6%
Ishiguro et al., 2014	-2.7748	0.5248	0.06	[0.02; 0.15]	4.9%
Lampe et al., 2019	-2.6995	0.2575	0.06	[0.04; 0.10]	6.1%
Cimatti et al., 2020	-2.3445	1.0057	0.09	[0.01; 0.41]	2.9%
Katheria et al., 2018	-2.1708	0.2927	0.10	[0.06; 0.17]	6.0%
Vesoulis et al., 2019	-2.1082	0.2568	0.11	[0.07; 0.17]	6.1%
Rojas et al, 2021	-1.8245	0.3411	0.14	[0.08; 0.24]	5.8%
Martini et al., 2022	-1.7914	0.4180	0.14	[0.07; 0.27]	5.4%
Alderliesten et al., 2013	-1.5856	0.2062	0.17	[0.12; 0.23]	6.3%
El-Dib et al., 2022	-1.3117	0.7370	0.21	[0.06; 0.53]	3.9%
Juul et al., 2020	-1.1808	0.3478	0.23	[0.13; 0.38]	5.7%
Da Costa et al., 2018	-1.1637	0.3286	0.24	[0.14; 0.37]	5.8%
Jiang et al., 2023	-0.9860	0.2345	0.27	[0.19; 0.37]	6.2%
Deshpande et al., 2020	-0.9445	0.3150	0.28	[0.17; 0.42]	5.9%
Farag et al., 2022	-0.7973	0.1882	0.31	[0.24; 0.39]	6.3%
**Random effects model (HK)**			**0.11**	**[0.07; 0.17]**	**100.0%**
Heterogeneity: $I^2 = 87\%$ [82%; 91%], $\tau^2 = 0.8793$, $p < 0.01$					
Test for subgroup differences: $\chi^2_1 = 3.90$, df = 1 ($p = 0.05$)					

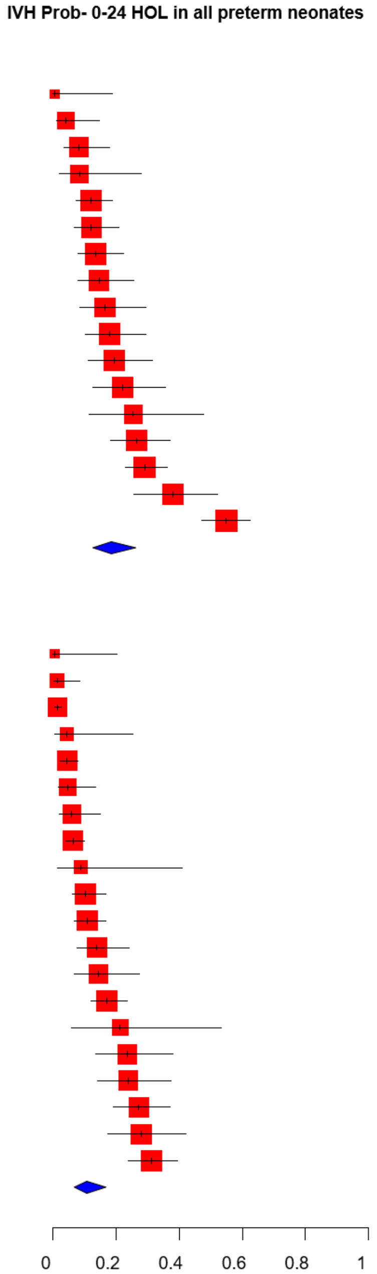

IVH: intraventricular haemorrhage; HOL: hours of life

© 2024 Nagy Zs et al. *JAMA Pediatrics.*

eFigure 8. IVH rate up to 48 HOL of all preterm infants in the subgroups before and after 2007

study	logit	se	Proportion	CI	w.random
**subgroup1 = before 2007**					
Amato et al., 1989	-2.1972	0.4711	0.10	[0.04; 0.22]	5.9%
Krediet et al., 2006	-1.6020	0.2515	0.17	[0.11; 0.25]	7.6%
Thorburn et al., 1982	-1.5177	0.2760	0.18	[0.11; 0.27]	7.4%
Van Bel et al., 2002	-1.3566	0.2720	0.20	[0.13; 0.31]	7.5%
Weindling et al., 1985	-1.3267	0.3120	0.21	[0.13; 0.33]	7.2%
Kluckow et al., 2000	-1.2992	0.2174	0.21	[0.15; 0.29]	7.8%
Meek et al., 1998	-1.0986	0.4715	0.25	[0.12; 0.46]	5.9%
Dolfin et al., 1983	-0.9474	0.2861	0.28	[0.18; 0.40]	7.4%
Rumack et al., 1985	-0.8184	0.3098	0.31	[0.19; 0.45]	7.2%
Dani et al., 2005	-0.7277	0.1612	0.33	[0.26; 0.40]	8.2%
McDonald et al., 1984	-0.5754	0.2947	0.36	[0.24; 0.50]	7.3%
Ment et al., 1984	-0.1942	0.3610	0.45	[0.29; 0.63]	6.8%
Perlman et al., 1983	0.0352	0.2649	0.51	[0.38; 0.64]	7.5%
Iyer et al., 2015	0.2412	0.4030	0.56	[0.37; 0.74]	6.4%
**Random effects model (HK)**			**0.28**	**[0.21; 0.36]**	**100.0%**
Heterogeneity: $I^2 = 76\%$ [60%; 86%], $\tau^2 = 0.2995$, $p < 0.01$					
**subgroup2 = after 2007**					
Galderisi et al., 2019	-5.2933	2.0050	0.00	[0.00; 0.20]	0.8%
Ikeda et al., 2014	-3.2507	0.5904	0.04	[0.01; 0.11]	4.1%
Schreiner et al., 2021	-2.8248	0.1873	0.06	[0.04; 0.08]	6.6%
Ishiguro et al., 2014	-2.2219	0.4386	0.10	[0.04; 0.20]	5.1%
Katheria et al., 2018	-2.1708	0.2927	0.10	[0.06; 0.17]	6.0%
Duppré et al., 2015	-1.9924	0.1951	0.12	[0.09; 0.17]	6.6%
Tanaka et al., 2022	-1.9277	0.3779	0.13	[0.06; 0.23]	5.5%
Lampe et al., 2019	-1.8012	0.1801	0.14	[0.10; 0.19]	6.6%
Martini et al., 2022	-1.7914	0.3919	0.14	[0.07; 0.26]	5.4%
Zanelli et al., 2021	-1.3285	0.1667	0.21	[0.16; 0.27]	6.7%
Alderliesten et al., 2013	-1.2610	0.1006	0.22	[0.19; 0.26]	6.9%
Vesoulis et al., 2019	-1.1764	0.1882	0.24	[0.18; 0.31]	6.6%
Rojas et al, 2021	-1.0986	0.2720	0.25	[0.16; 0.36]	6.1%
Da Costa et al., 2018	-0.8043	0.2917	0.31	[0.20; 0.44]	6.0%
Juul et al., 2020	-0.7345	0.6267	0.32	[0.12; 0.62]	3.9%
Cimatti et al., 2020	-0.6190	0.4690	0.35	[0.18; 0.57]	4.9%
Deshpande et al., 2020	-0.4055	0.2887	0.40	[0.27; 0.54]	6.0%
El-Dib et al., 2022	-0.3921	0.2739	0.40	[0.28; 0.54]	6.1%
**Random effects model (HK)**			**0.18**	**[0.12; 0.25]**	**100.0%**
Heterogeneity: $I^2 = 87\%$ [82%; 91%], $\tau^2 = 0.5175$, $p < 0.01$					
Test for subgroup differences: $\chi^2_1 = 4.77$, df = 1 ($p = 0.03$)					

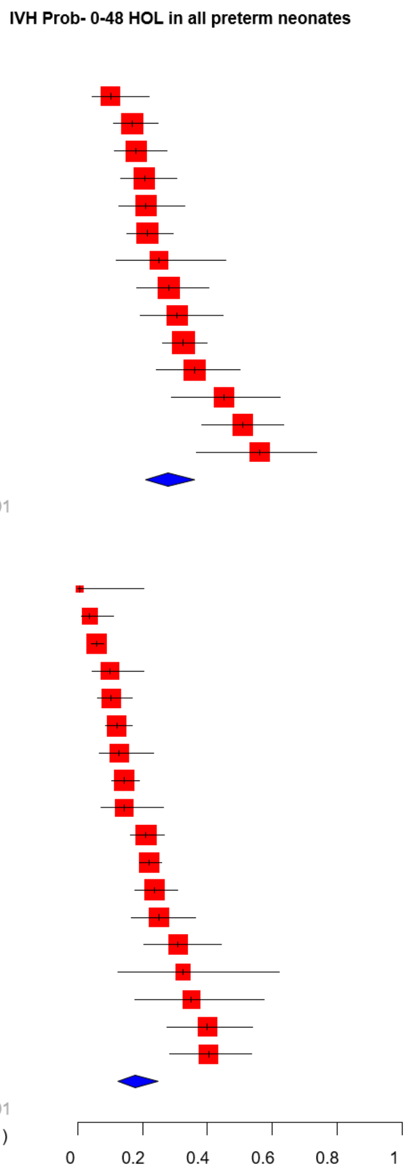

IVH: intraventricular haemorrhage; HOL: hours of life

© 2024 Nagy Zs et al. *JAMA Pediatrics.*

eFigure 9. IVH rate up to 72 HOL of all preterm infants in the subgroups before and after 2007

study	logit	se	proportion	CI	w.random
**subgroup1 = before 2007**					
Zanardo et al., 2008	-2.5915	0.2318	0.07	[0.05; 0.11]	6.5%
Salafia et al., 1995	-2.1072	0.1593	0.11	[0.08; 0.14]	6.9%
Sarkar et al., 2005	-1.7728	0.3601	0.15	[0.08; 0.26]	5.7%
Krediet et al., 2006	-1.6020	0.2515	0.17	[0.11; 0.25]	6.4%
Kluckow et al., 2000	-1.2992	0.2174	0.21	[0.15; 0.29]	6.6%
Van Bel et al., 2002	-1.1472	0.2564	0.24	[0.16; 0.34]	6.4%
Thorburn et al., 1982	-0.9961	0.2386	0.27	[0.19; 0.37]	6.5%
Amato et al., 1989	-0.9445	0.3150	0.28	[0.17; 0.42]	6.0%
Perlman et al., 1986	-0.9430	0.1086	0.28	[0.24; 0.33]	7.0%
Meek et al., 1998	-0.8871	0.4492	0.29	[0.15; 0.50]	5.1%
Dolfin et al., 1983	-0.7095	0.2738	0.33	[0.22; 0.46]	6.3%
Rumack et al., 1985	-0.4566	0.2932	0.39	[0.26; 0.53]	6.1%
Yang et al., 1990	-0.3158	0.2223	0.42	[0.32; 0.53]	6.6%
McDonald et al., 1984	-0.2412	0.2849	0.44	[0.31; 0.58]	6.2%
Iyer et al., 2015	0.2412	0.4029	0.56	[0.37; 0.74]	5.4%
Perlman et al., 1983	0.2468	0.2668	0.56	[0.43; 0.68]	6.3%
**Random effects model (HK)**			**0.27**	**[0.20; 0.36]**	**100.0%**
Heterogeneity: $I^2 = 90\%$ [86%; 93%], $\tau^2 = 0.5525$, $p < 0.01$					
**subgroup2 = after 2007**					
Kalani et al., 2016	-2.8672	0.4597	0.05	[0.02; 0.12]	4.0%
Ikeda et al., 2014	-2.5137	0.4243	0.07	[0.03; 0.16]	4.1%
Schreiner et al., 2021	-2.3892	0.1560	0.08	[0.06; 0.11]	5.4%
Ishiguro et al., 2014	-1.8974	0.3122	0.13	[0.08; 0.22]	4.7%
Najib et al., 2018	-1.6273	0.3301	0.16	[0.09; 0.27]	4.6%
Vesoulis et al., 2020	-1.6030	0.1969	0.17	[0.12; 0.23]	5.2%
Duppré et al., 2015	-1.5437	0.1662	0.18	[0.13; 0.23]	5.3%
Tanaka et al., 2022	-1.4468	0.3210	0.19	[0.11; 0.31]	4.7%
Martini et al., 2022	-1.3382	0.2892	0.21	[0.13; 0.32]	4.8%
Noori et al., 2014	-1.0413	0.4750	0.26	[0.12; 0.47]	3.9%
Shan et al., 2023	-0.9164	0.1436	0.29	[0.23; 0.35]	5.4%
Lampe et al., 2019	-0.8511	0.1369	0.30	[0.25; 0.36]	5.4%
Rojas et al, 2021	-0.8208	0.2559	0.31	[0.21; 0.42]	5.0%
Da Costa et al., 2018	-0.8043	0.2917	0.31	[0.20; 0.44]	4.8%
Vesoulis et al., 2019	-0.7903	0.1723	0.31	[0.24; 0.39]	5.3%
Katheria et al., 2014	-0.7690	0.2777	0.32	[0.21; 0.44]	4.9%
Juul et al., 2020	-0.7345	0.6267	0.32	[0.12; 0.62]	3.2%
Cimatti et al., 2020	-0.4055	0.4562	0.40	[0.21; 0.62]	4.0%
Deshpande et al., 2020	-0.1603	0.2838	0.46	[0.33; 0.60]	4.8%
Farag et al., 2022	-0.1519	0.1746	0.46	[0.38; 0.55]	5.3%
Jiang et al., 2023	-0.0869	0.2088	0.48	[0.38; 0.58]	5.2%
**Random effects model (HK)**			**0.24**	**[0.18; 0.31]**	**100.0%**
Heterogeneity: $I^2 = 90\%$ [86%; 93%], $\tau^2 = 0.4852$, $p < 0.01$					
Test for subgroup differences: $\chi^2_1 = 0.46$, df = 1 ($p = 0.50$)					

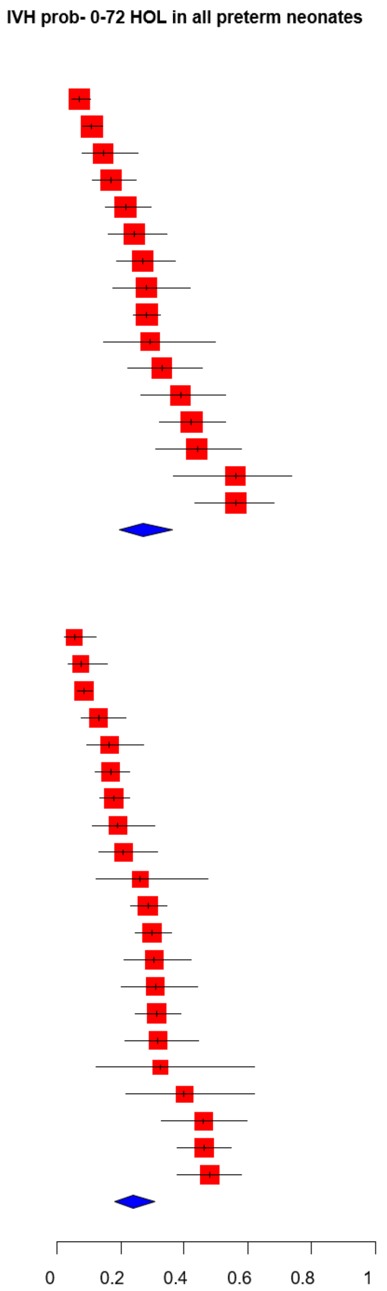

IVH: intraventricular haemorrhage; HOL: hours of life

© 2024 Nagy Zs et al. *JAMA Pediatrics.*

eFigure 10: IVH rate up to 6 HOL in all IVH cases in the subgroups before and after 2007

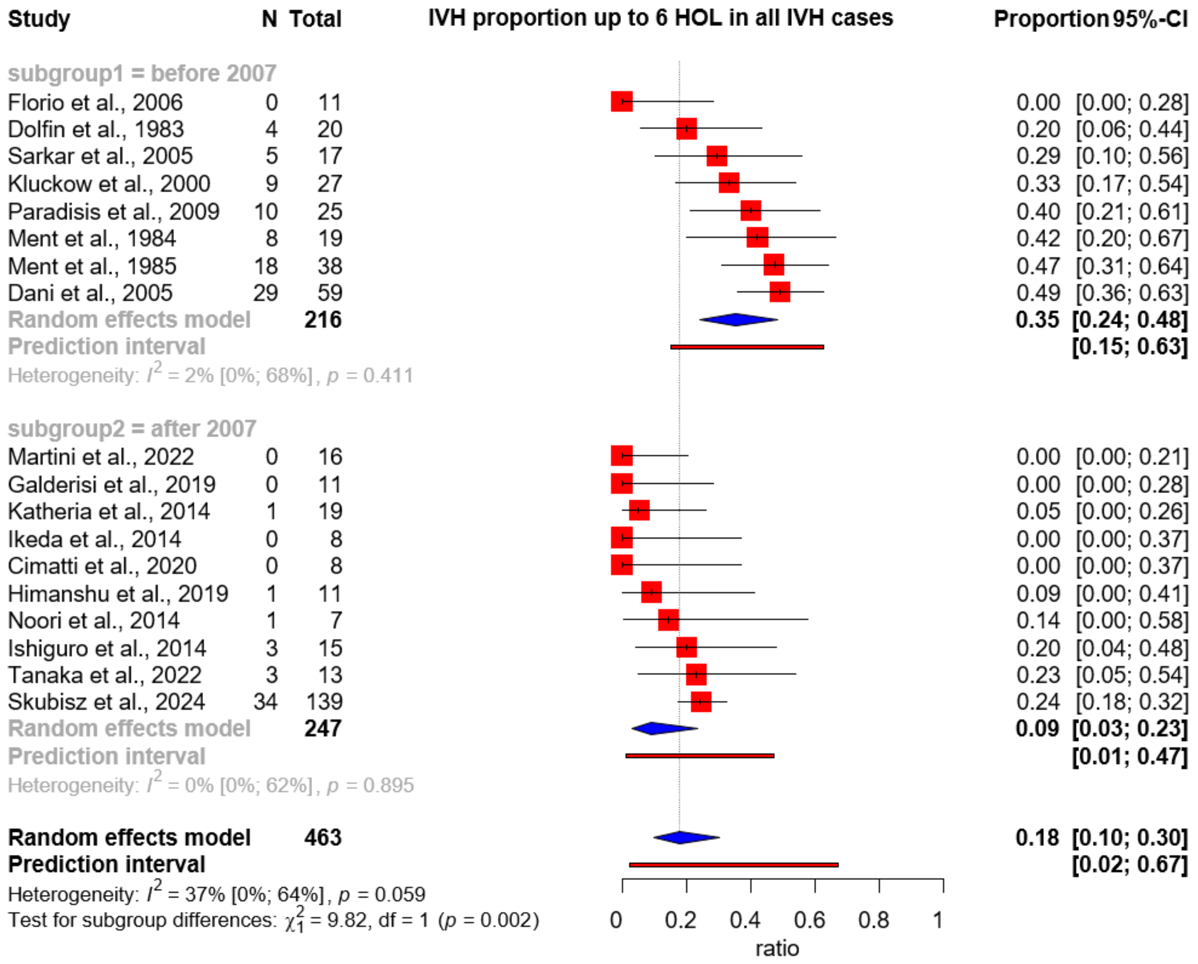

IVH, intraventricular hemorrhage; HOL, hours of life

© 2024 Nagy Zs et al. *JAMA Pediatrics.*

eFigure 11: IVH rate up to 12 HOL in all IVH cases in the subgroups before and after 2007

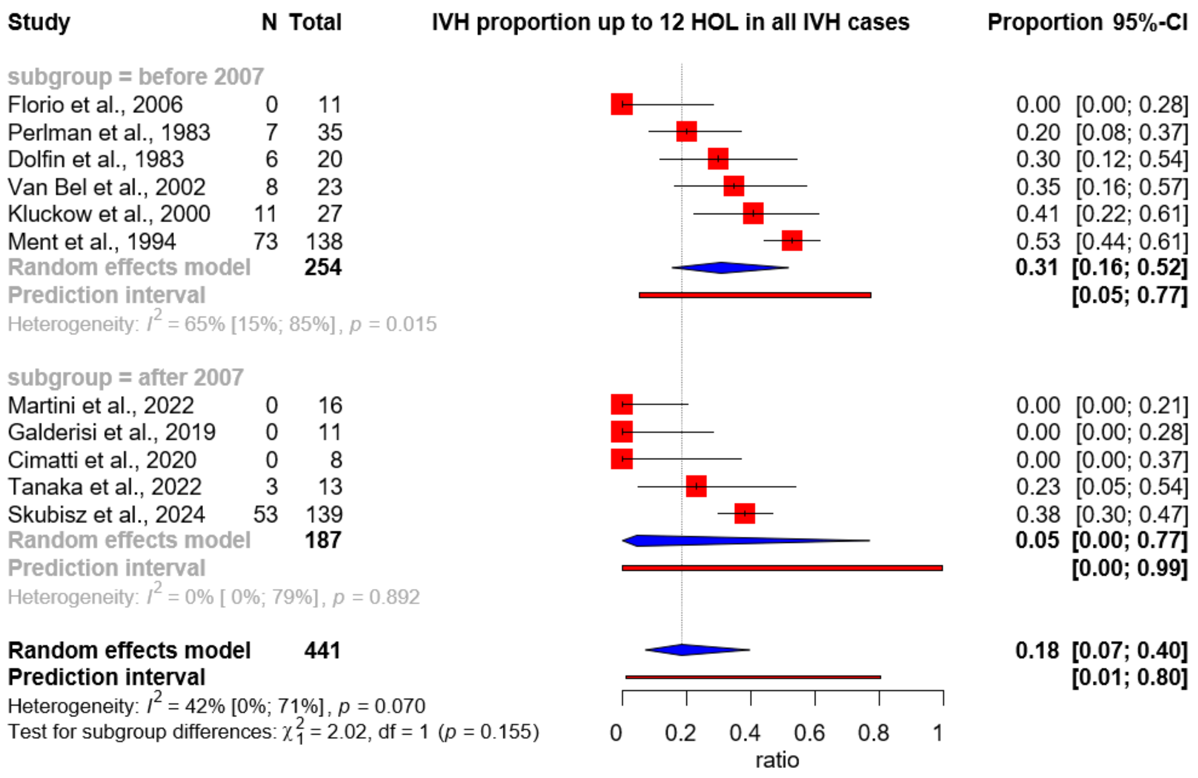

IVH, intraventricular hemorrhage; HOL, hours of life

© 2024 Nagy Zs et al. *JAMA Pediatrics.*

eFigure 12: IVH rate up to 24 HOL in all IVH cases in the subgroups before and after 2007

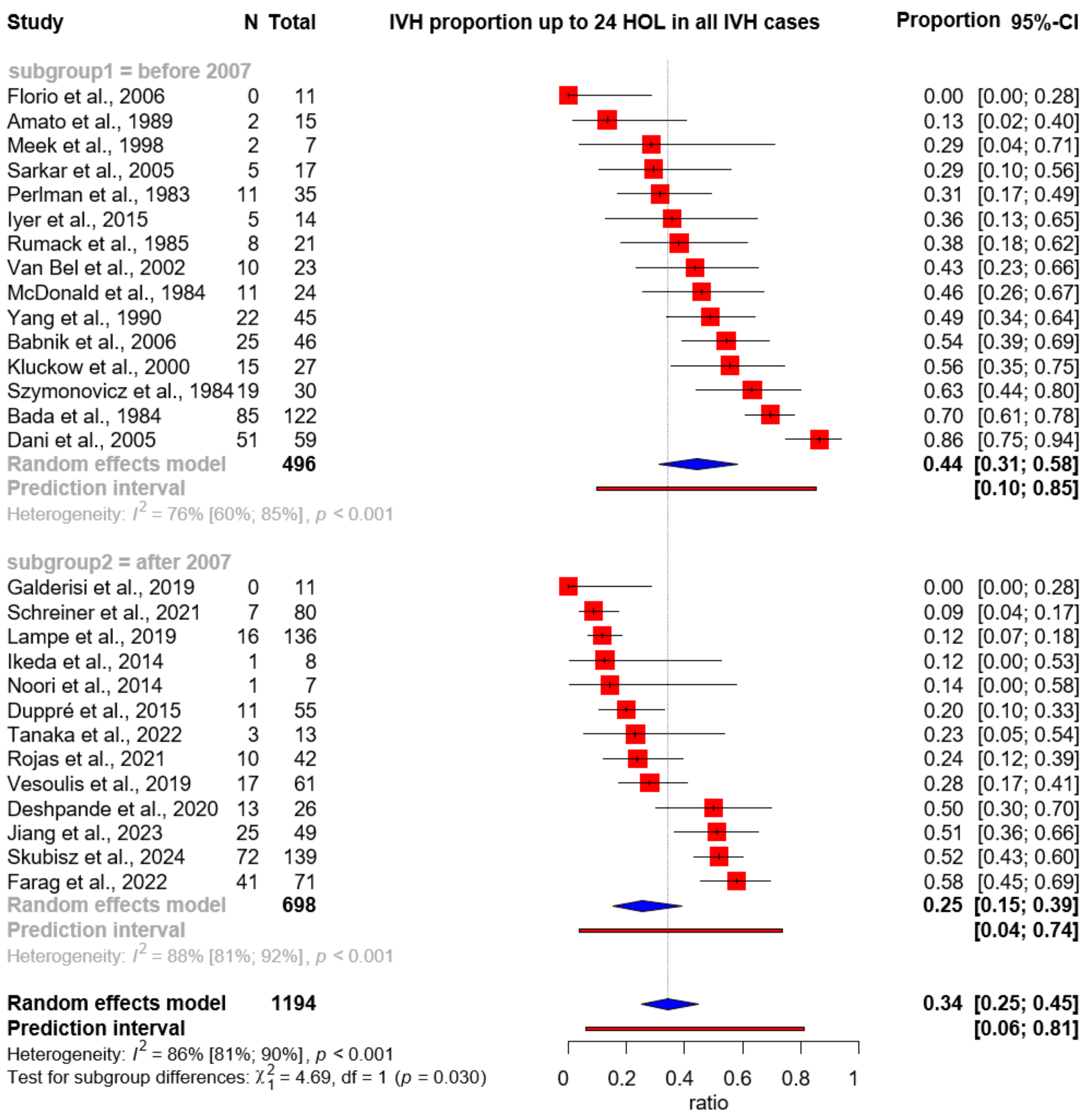

IVH, intraventricular hemorrhage; HOL, hours of life

© 2024 Nagy Zs et al. *JAMA Pediatrics.*

eFigure 13.: IVH rate up to 48 HOL in all IVH cases in the subgroups before and after 2007

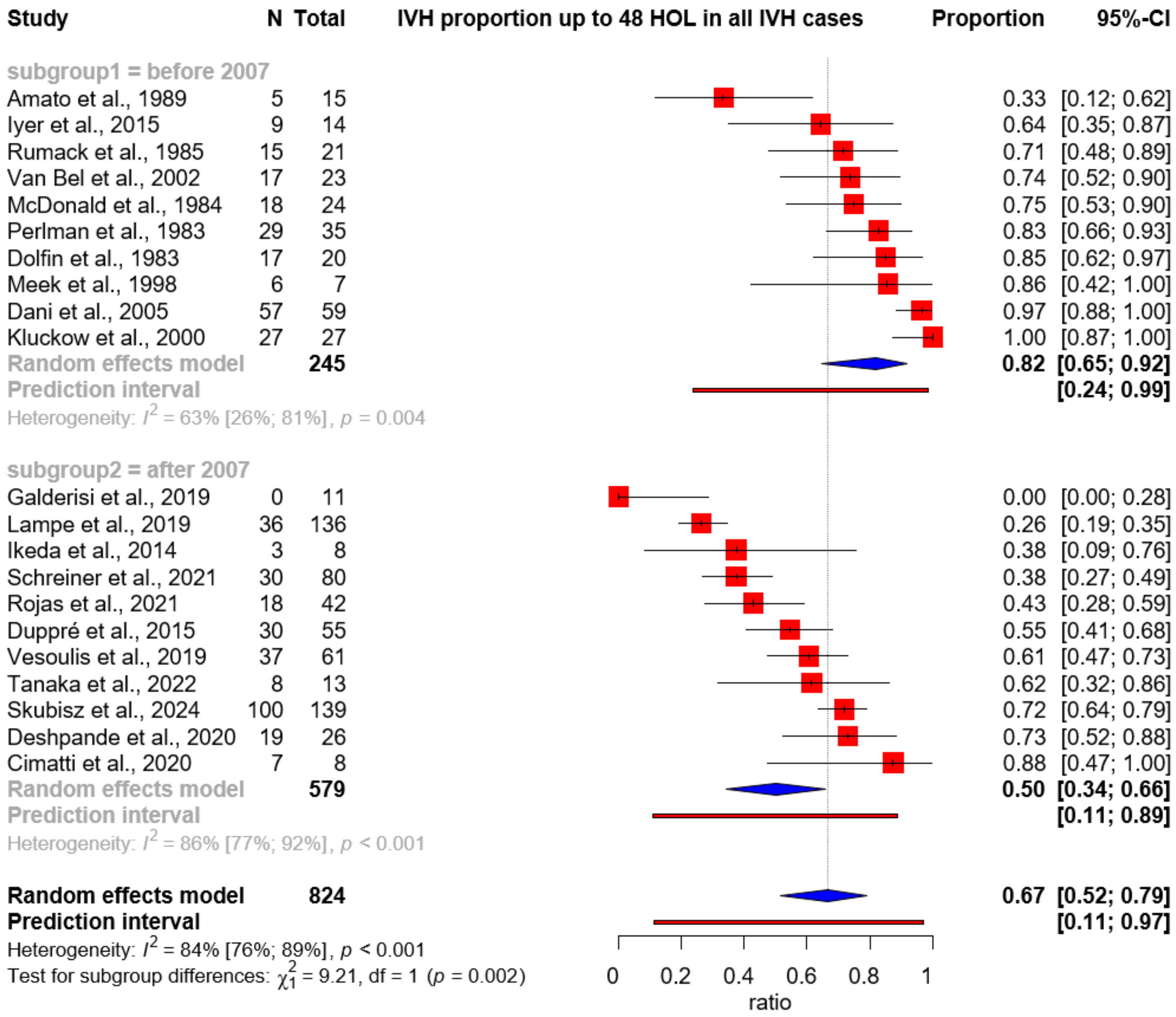

IVH, intraventricular hemorrhage; HOL, hours of life

© 2024 Nagy Zs et al. *JAMA Pediatrics.*

eFigure 14.: IVH rate up to 72 HOL in all IVH cases in the subgroups before and after 2007

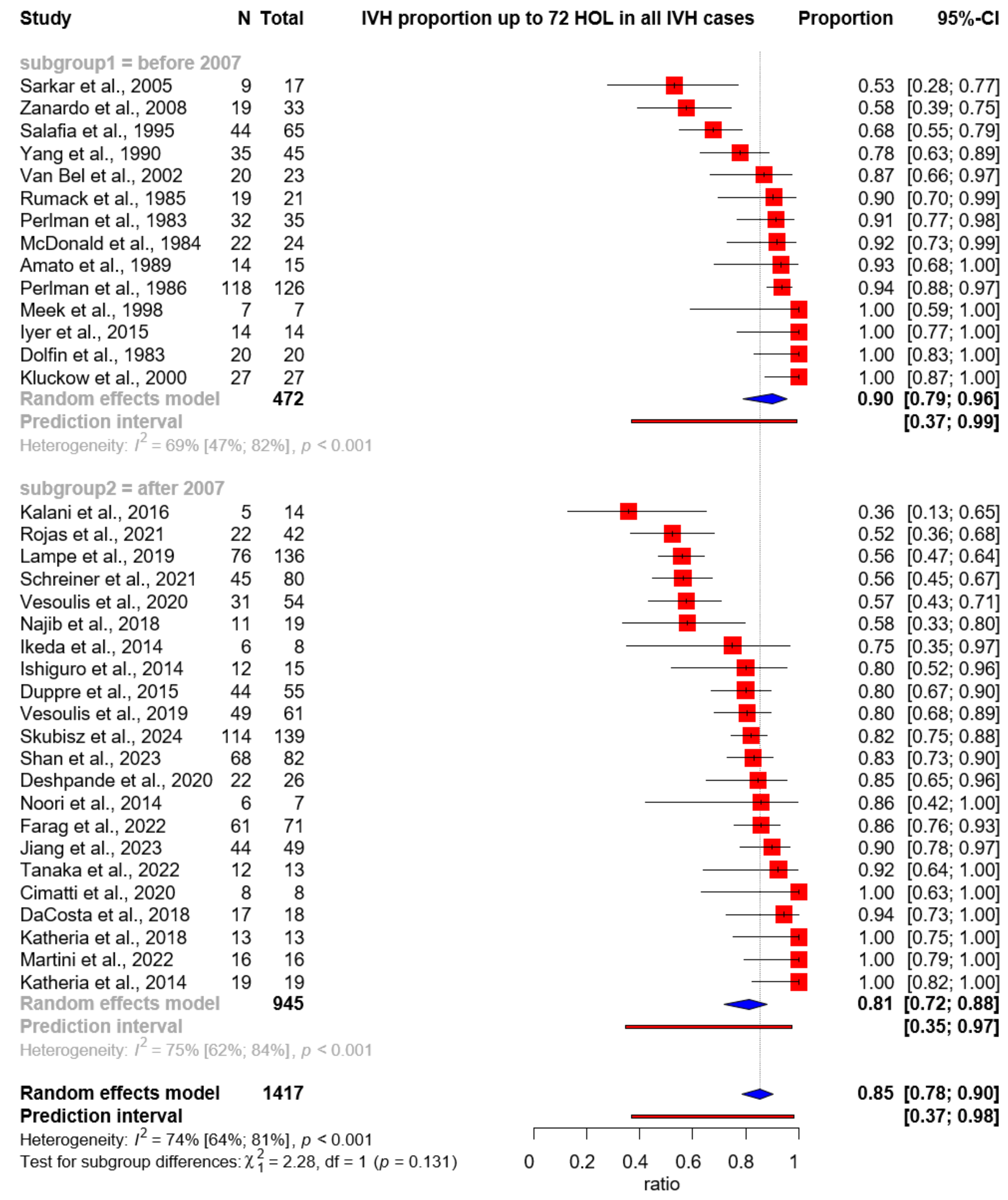

IVH, intraventricular hemorrhage; HOL, hours of life

© 2024 Nagy Zs et al. *JAMA Pediatrics.*

eFigure 15. IVH rate up to 6 HOL by GA limits in GA<28, GA<32 in the subgroup after 2007

study	logit	se	prop	ci	w.random
**subgroup1 = GA<28 weeks**					
Noori et al., 2014	-3.0905	1.0214	0.04	[0.01; 0.25]	19.2%
Tanaka et al., 2022	-3.0123	0.5914	0.05	[0.02; 0.14]	30.5%
El-Dib et al., 2022	-1.3769	0.9111	0.20	[0.04; 0.60]	21.7%
Juul et al., 2020	-1.3517	0.6545	0.21	[0.07; 0.48]	28.6%
**Random effects model (HK)**			**0.10**	**[0.02; 0.34]**	**100.0%**
Heterogeneity: I^2 = 42% [0%; 80%] , τ^2 = 0.4235 , p = 0.16					
**subgroup2 = GA<32 weeks**					
Cimatti et al., 2020	-5.2933	3.1702	0.00	[0.00; 0.72]	2.5%
Galderisi et al., 2019	-5.2933	2.0050	0.00	[0.00; 0.20]	5.6%
Martini et al., 2022	-5.2933	1.6157	0.00	[0.00; 0.11]	7.8%
Katheria et al., 2014	-4.0755	1.0048	0.02	[0.00; 0.11]	14.6%
Himanshu et al., 2019	-3.9124	1.0096	0.02	[0.00; 0.13]	14.6%
Katheria et al., 2018	-2.6827	0.4056	0.06	[0.03; 0.13]	27.0%
Da Costa et al., 2018	-1.3664	0.3650	0.20	[0.11; 0.34]	27.9%
**Random effects model (HK)**			**0.04**	**[0.01; 0.13]**	**100.0%**
Heterogeneity: I^2 = 69% [33%; 86%] , τ^2 = 1.5324 , p < 0.01					
Test for subgroup differences: χ^2_1 = 2.20, df = 1 (p = 0.14)					

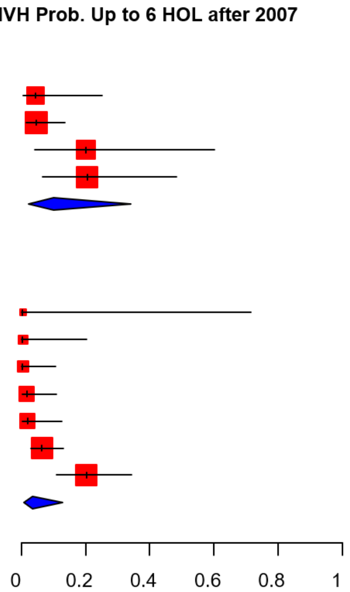

IVH: intraventricular hemorrhage; HOL: hours of life; GA: gestational age

eFigure 16. IVH rate up to 12 HOL by GA limits in GA<28, GA<32 in the subgroup after 2007

study	logit	se	proportion	CI	w.random
**subgroup1 = GA<28 weeks**					
Noori et al., 2014	-3.0910	1.0225	0.04	[0.01; 0.25]	11.8%
Bates et al., 2015	-2.5257	0.3674	0.07	[0.04; 0.14]	44.6%
Juul et al., 2020	-1.3517	0.7721	0.21	[0.05; 0.54]	18.6%
El-Dib et al., 2022	-1.3117	0.6275	0.21	[0.07; 0.48]	25.0%
**Random effects model (HK)**			**0.11**	**[0.04; 0.31]**	**100.0%**
Heterogeneity: I^2 = 37% [0%; 78%] , τ^2 = 0.2332 , p = 0.19					
**subgroup2 = GA<32 weeks**					
Cimatti et al., 2020	-5.2933	3.1702	0.00	[0.00; 0.72]	0.9%
Galderisi et al., 2019	-5.2933	2.0050	0.00	[0.00; 0.20]	2.1%
Martini et al., 2022	-5.2933	1.6157	0.00	[0.00; 0.11]	3.2%
Katheria et al., 2018	-2.6827	0.4056	0.06	[0.03; 0.13]	24.7%
Alderliesten et al., 2013	-2.0826	0.1309	0.11	[0.09; 0.14]	42.0%
Da Costa et al., 2018	-1.3664	0.3650	0.20	[0.11; 0.34]	27.1%
**Random effects model (HK)**			**0.07**	**[0.02; 0.25]**	**100.0%**
Heterogeneity: I^2 = 63% [11%; 85%] , τ^2 = 0.7880 , p = 0.02					
Test for subgroup differences: χ^2_1 = 0.45, df = 1 (p = 0.50)					

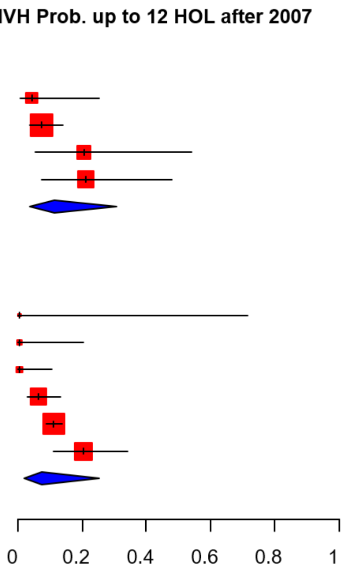

IVH, intraventricular hemorrhage; HOL, hours of life; GA: gestational age

© 2024 Nagy Zs et al. *JAMA Pediatrics.*

eFigure 17. IVH rate up to 24 HOL by GA limits in GA<28, GA<32 in the subgroup after 2007

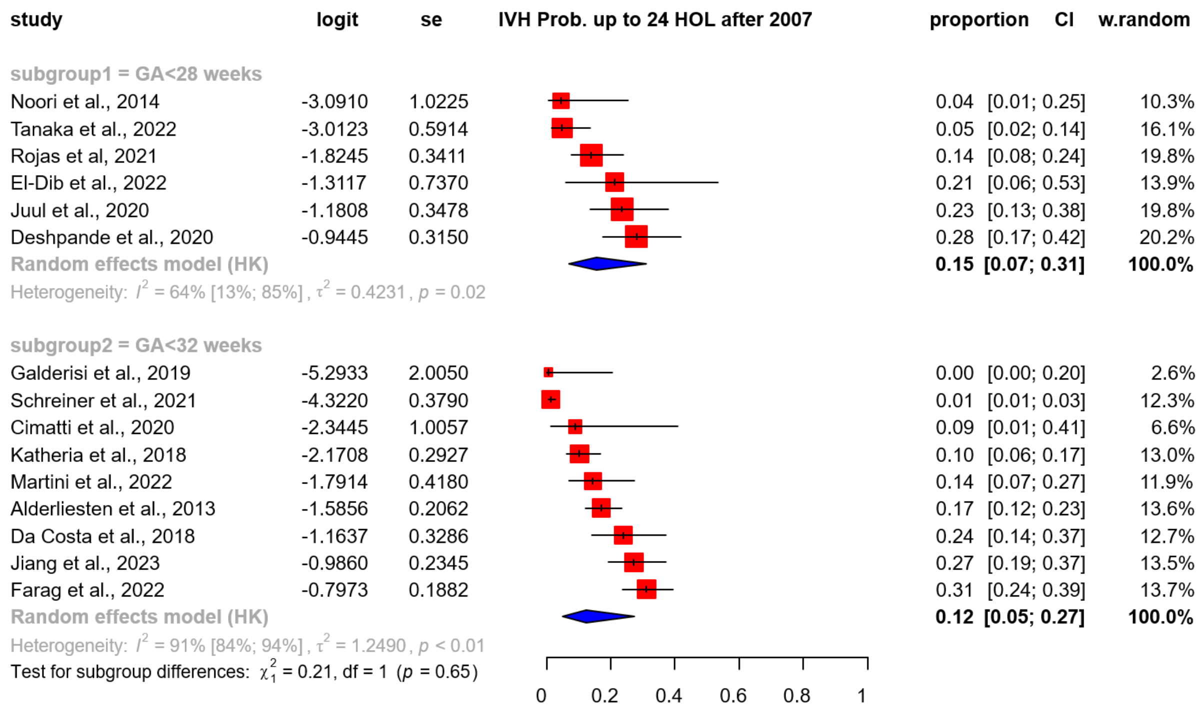

IVH, intraventricular hemorrhage; HOL, hours of life; GA: gestational age

eFigure 18. IVH rate up to 48 HOL by GA limits in GA<28, GA<32 in the subgroup after 2007

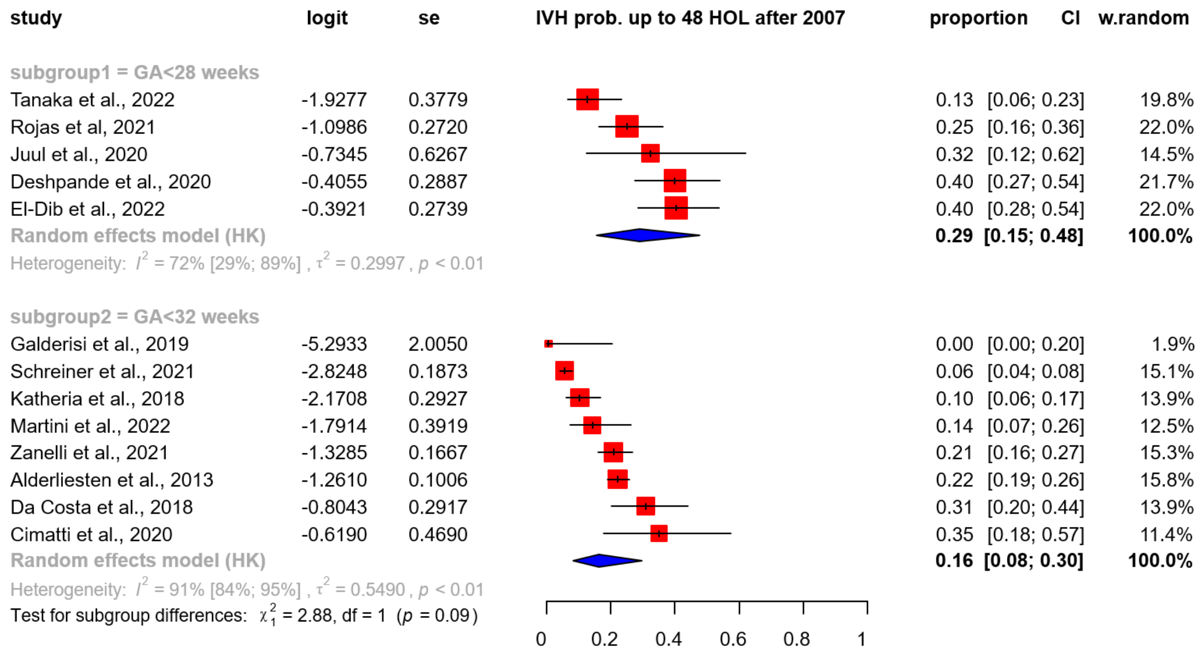

IVH, intraventricular hemorrhage; HOL, hours of life; GA: gestational age

© 2024 Nagy Zs et al. *JAMA Pediatrics.*

eFigure 19. IVH rate up to 72 HOL by GA limits in GA<28, GA<32 in the subgroup after 2007

study	logit	se	proportion	CI	w.random
**subgroup1 = GA<28 weeks**					
Tanaka et al., 2022	-1.4468	0.3210	0.19	[0.11; 0.31]	21.5%
Noori et al., 2014	-1.0413	0.4750	0.26	[0.12; 0.47]	18.3%
Rojas et al, 2021	-0.8208	0.2559	0.31	[0.21; 0.42]	22.8%
Juul et al., 2020	-0.7345	0.6267	0.32	[0.12; 0.62]	15.2%
Deshpande et al., 2020	-0.1603	0.2838	0.46	[0.33; 0.60]	22.3%
**Random effects model (HK)**			**0.30**	**[0.19; 0.45]**	**100.0%**
Heterogeneity: I^2 = 58% [0%; 84%] , τ^2 = 0.1673 , p = 0.05					
**subgroup2 = GA<32 weeks**					
Kalani et al., 2016	-2.8672	0.4597	0.05	[0.02; 0.12]	9.3%
Schreiner et al., 2021	-2.3892	0.1560	0.08	[0.06; 0.11]	12.2%
Vesoulis et al., 2020	-1.6030	0.1969	0.17	[0.12; 0.23]	11.9%
Martini et al., 2022	-1.3382	0.2892	0.21	[0.13; 0.32]	11.1%
Da Costa et al., 2018	-0.8043	0.2917	0.31	[0.20; 0.44]	11.1%
Katheria et al., 2014	-0.7690	0.2777	0.32	[0.21; 0.44]	11.2%
Cimatti et al., 2020	-0.4055	0.4562	0.40	[0.21; 0.62]	9.4%
Farag et al., 2022	-0.1519	0.1746	0.46	[0.38; 0.55]	12.1%
Jiang et al., 2023	-0.0869	0.2088	0.48	[0.38; 0.58]	11.8%
**Random effects model (HK)**			**0.24**	**[0.13; 0.40]**	**100.0%**
Heterogeneity: I^2 = 95% [92%; 96%] , τ^2 = 0.8356 , p < 0.01					
Test for subgroup differences: χ^2_1 = 0.66, df = 1 (p = 0.42)					

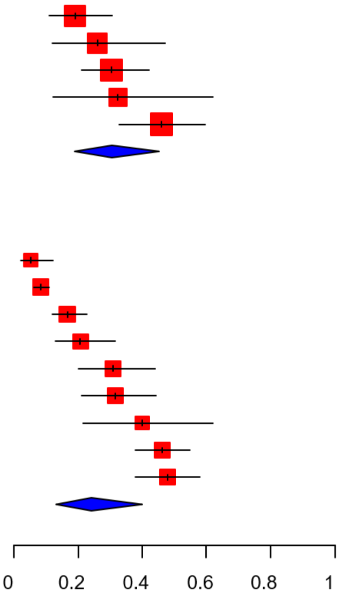

IVH, intraventricular hemorrhage; HOL, hours of life; GA: gestational age

eFigure 20. IVH rate up to 6 HOL in GA<32 in the subgroups before and after 2007

study	logit	se	proportion	CI	w.random
**subgroup1 = before 2007**					
Florio et al., 2006	-5.2933	1.9474	0.00	[0.00; 0.19]	11.8%
Weindling et al., 1985	-2.6744	0.5171	0.06	[0.02; 0.16]	42.1%
Rumack et al., 1985	-1.6339	0.3864	0.16	[0.08; 0.29]	46.1%
**Random effects model (HK)**			**0.08**	**[0.00; 0.67]**	**100.0%**
Heterogeneity: I^2 = 63% [0%; 89%] , τ^2 = 0.6001 , p = 0.07					
**subgroup2 = after 2007**					
Cimatti et al., 2020	-5.2933	3.1702	0.00	[0.00; 0.72]	2.9%
Galderisi et al., 2019	-5.2933	2.0050	0.00	[0.00; 0.20]	6.3%
Martini et al., 2022	-5.2933	1.6157	0.00	[0.00; 0.11]	8.7%
Katheria et al., 2014	-4.0755	1.0048	0.02	[0.00; 0.11]	15.3%
Himanshu et al., 2019	-3.9124	1.0096	0.02	[0.00; 0.13]	15.2%
Katheria et al., 2018	-2.6827	0.4056	0.06	[0.03; 0.13]	25.4%
Da Costa et al., 2018	-1.3664	0.3650	0.20	[0.11; 0.34]	26.1%
**Random effects model (HK)**			**0.04**	**[0.01; 0.13]**	**100.0%**
Heterogeneity: I^2 = 69% [33%; 86%] , τ^2 = 1.5324 , p < 0.01					
Test for subgroup differences: χ^2_1 = 1.03, df = 1 (p = 0.31)					

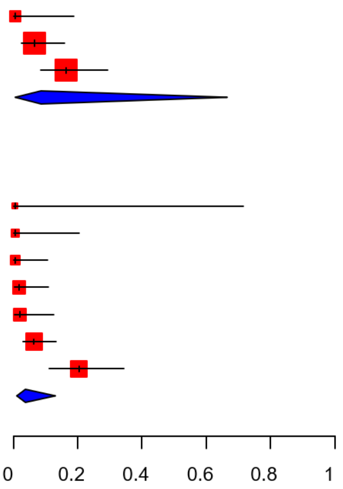

IVH, intraventricular hemorrhage; HOL, hours of life; GA: gestational age

© 2024 Nagy Zs et al. *JAMA Pediatrics.*

eFigure 21. IVH rate up to 12 HOL in GA<32 in the subgroups before and after 2007

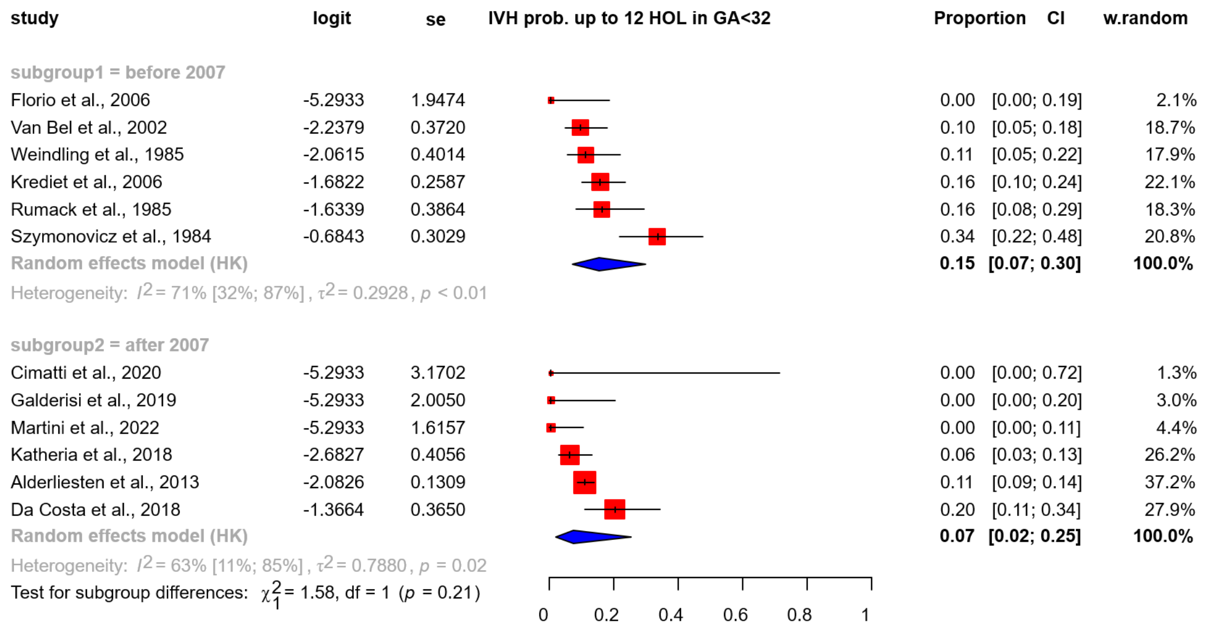

IVH, intraventricular hemorrhage; HOL, hours of life; GA: gestational age

eFigure 22. IVH rate up to 24 HOL in GA<32 in the subgroups before and after 2007

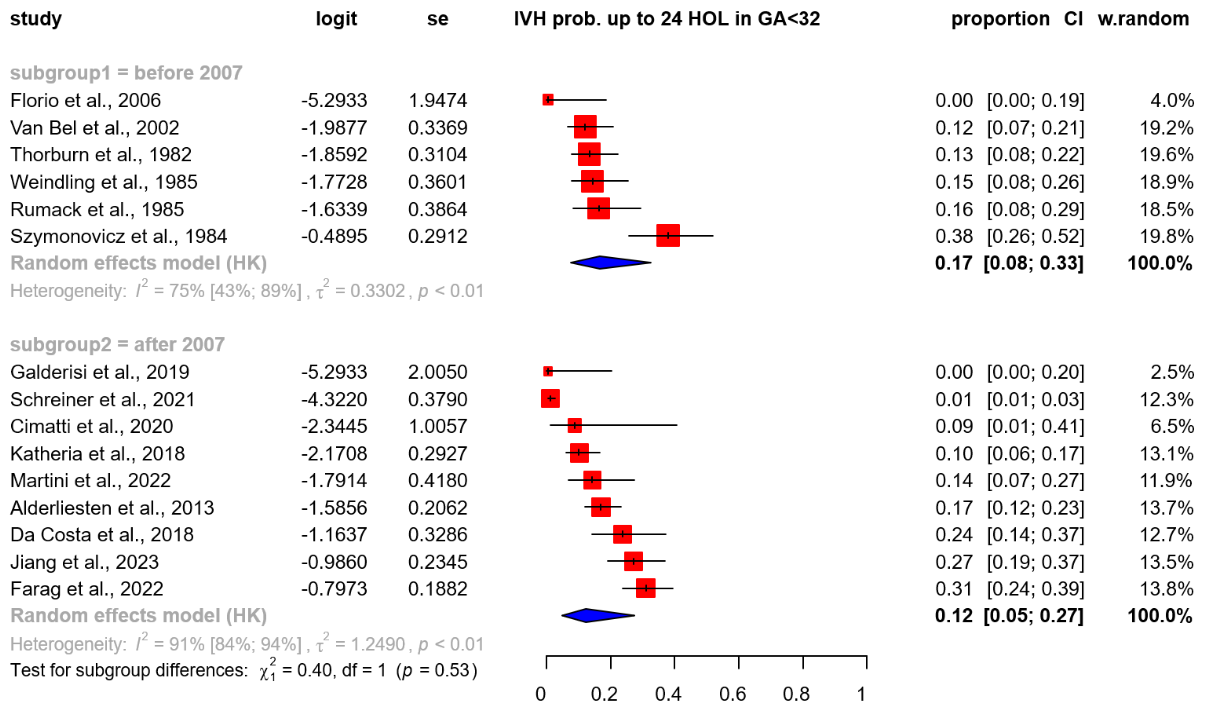

IVH, intraventricular hemorrhage; HOL, hours of life; GA: gestational age

© 2024 Nagy Zs et al. *JAMA Pediatrics.*

eFigure 23. IVH rate up to 48 HOL in GA<32 in the subgroups before and after 2007

study	logit	se	proportion	CI	w.random
**subgroup1 = before 2007**					
Krediet et al., 2006	-1.6020	0.2515	0.17	[0.11; 0.25]	20.9%
Thorburn et al., 1982	-1.5177	0.2760	0.18	[0.11; 0.27]	20.2%
Van Bel et al., 2002	-1.3566	0.2720	0.20	[0.13; 0.31]	20.4%
Weindling et al., 1985	-1.3267	0.3120	0.21	[0.13; 0.33]	19.2%
Rumack et al., 1985	-0.8184	0.3098	0.31	[0.19; 0.45]	19.3%
**Random effects model (HK)**			**0.20**	**[0.15; 0.27]**	**100.0%**
Heterogeneity: $I^2 = 7\%$ [0%; 81%], $\tau^2 = 0.0015$, $p = 0.36$					
**subgroup2 = after 2007**					
Galderisi et al., 2019	-5.2933	2.0050	0.00	[0.00; 0.20]	1.3%
Schreiner et al., 2021	-2.8248	0.1873	0.06	[0.04; 0.08]	15.8%
Katheria et al., 2018	-2.1708	0.2927	0.10	[0.06; 0.17]	13.8%
Martini et al., 2022	-1.7914	0.3919	0.14	[0.07; 0.26]	11.8%
Zanelli et al., 2021	-1.3285	0.1667	0.21	[0.16; 0.27]	16.2%
Alderliesten et al., 2013	-1.2610	0.1006	0.22	[0.19; 0.26]	17.0%
Da Costa et al., 2018	-0.8043	0.2917	0.31	[0.20; 0.44]	13.8%
Cimatti et al., 2020	-0.6190	0.4690	0.35	[0.18; 0.57]	10.3%
**Random effects model (HK)**			**0.16**	**[0.08; 0.30]**	**100.0%**
Heterogeneity: $I^2 = 91\%$ [84%; 95%], $\tau^2 = 0.5490$, $p < 0.01$					
Test for subgroup differences: $\chi^2_1 = 0.64$, df = 1 ($p = 0.42$)					

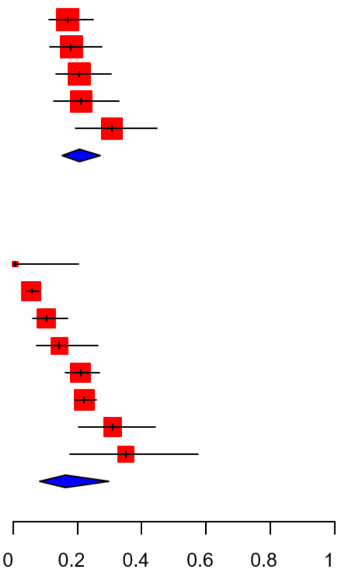

IVH, intraventricular hemorrhage; HOL, hours of life; GA: gestational age

eFigure 24. IVH rate up to 72 HOL in GA<32 in the subgroups before and after 2007

study	logit	se	proportion	CI	w.random
**subgroup1 = before 2007**					
Salafia et al., 1995	-2.1072	0.1593	0.11	[0.08; 0.14]	21.1%
Krediet et al., 2006	-1.6020	0.2515	0.17	[0.11; 0.25]	19.9%
Van Bel et al., 2002	-1.1472	0.2564	0.24	[0.16; 0.34]	19.8%
Thorburn et al., 1982	-0.9961	0.2386	0.27	[0.19; 0.37]	20.1%
Rumack et al., 1985	-0.4566	0.2932	0.39	[0.26; 0.53]	19.2%
**Random effects model (HK)**			**0.22**	**[0.11; 0.38]**	**100.0%**
Heterogeneity: $I^2 = 88\%$ [75%; 94%], $\tau^2 = 0.3428$, $p < 0.01$					
**subgroup2 = after 2007**					
Kalani et al., 2016	-2.8672	0.4597	0.05	[0.02; 0.12]	9.4%
Schreiner et al., 2021	-2.3892	0.1560	0.08	[0.06; 0.11]	12.1%
Vesoulis et al., 2020	-1.6030	0.1969	0.17	[0.12; 0.23]	11.9%
Martini et al., 2022	-1.3382	0.2892	0.21	[0.13; 0.32]	11.1%
Da Costa et al., 2018	-0.8043	0.2917	0.31	[0.20; 0.44]	11.1%
Katheria et al., 2014	-0.7690	0.2777	0.32	[0.21; 0.44]	11.2%
Cimatti et al., 2020	-0.4055	0.4562	0.40	[0.21; 0.62]	9.4%
Farag et al., 2022	-0.1519	0.1746	0.46	[0.38; 0.55]	12.0%
Jiang et al., 2023	-0.0869	0.2088	0.48	[0.38; 0.58]	11.8%
**Random effects model (HK)**			**0.24**	**[0.13; 0.40]**	**100.0%**
Heterogeneity: $I^2 = 95\%$ [92%; 96%], $\tau^2 = 0.8356$, $p < 0.01$					
Test for subgroup differences: $\chi^2_1 = 0.11$, df = 1 ($p = 0.74$)					

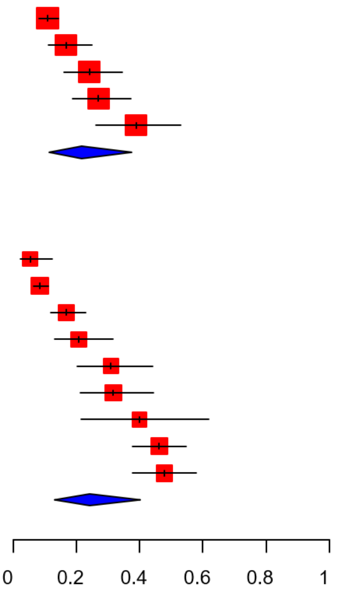

IVH, intraventricular hemorrhage; HOL, hours of life; GA: gestational age

© 2024 Nagy Zs et al. *JAMA Pediatrics.*

eFigure 25. IVH rate up to 6 HOL in GA<28 in the subgroups before and after 2007

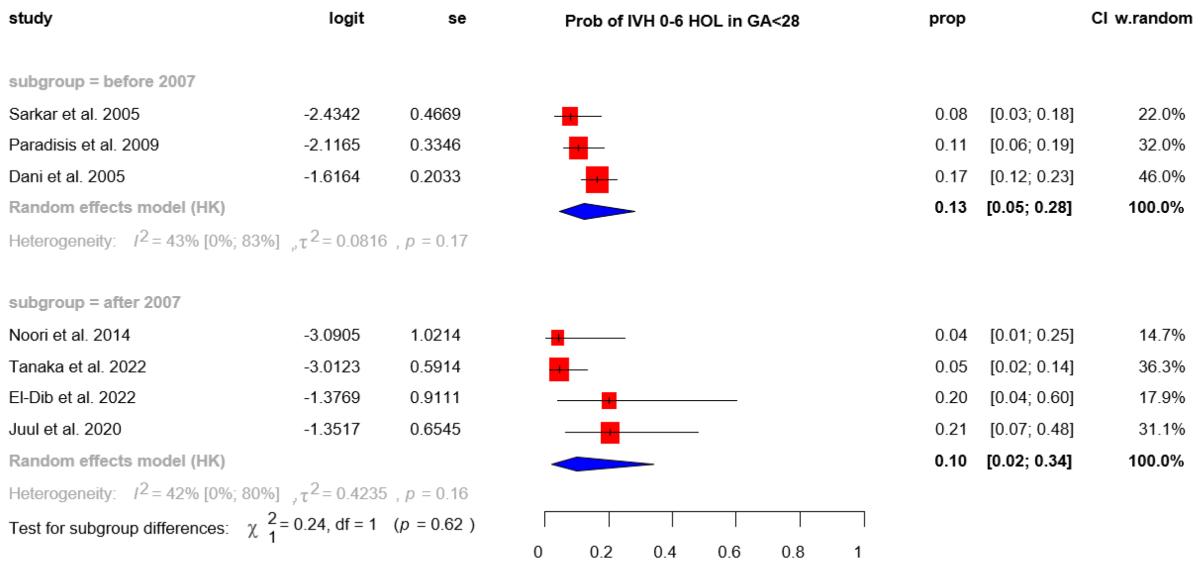

IVH, intraventricular hemorrhage; HOL, hours of life; GA: gestational age

eFigure 26. Overall IVH rate up to 12 HOL in GA<28 in the subgroups before and after 2007

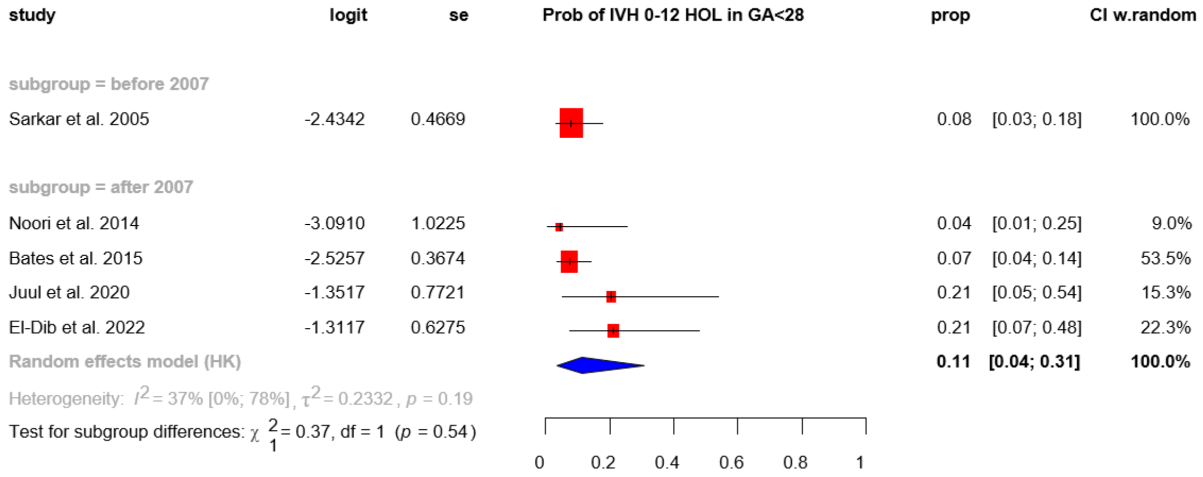

IVH, intraventricular hemorrhage; HOL, hours of life; GA: gestational age

© 2024 Nagy Zs et al. *JAMA Pediatrics.*

eFigure 27. IVH rate up to 24 HOL in GA<28 in the subgroups before and after 2007

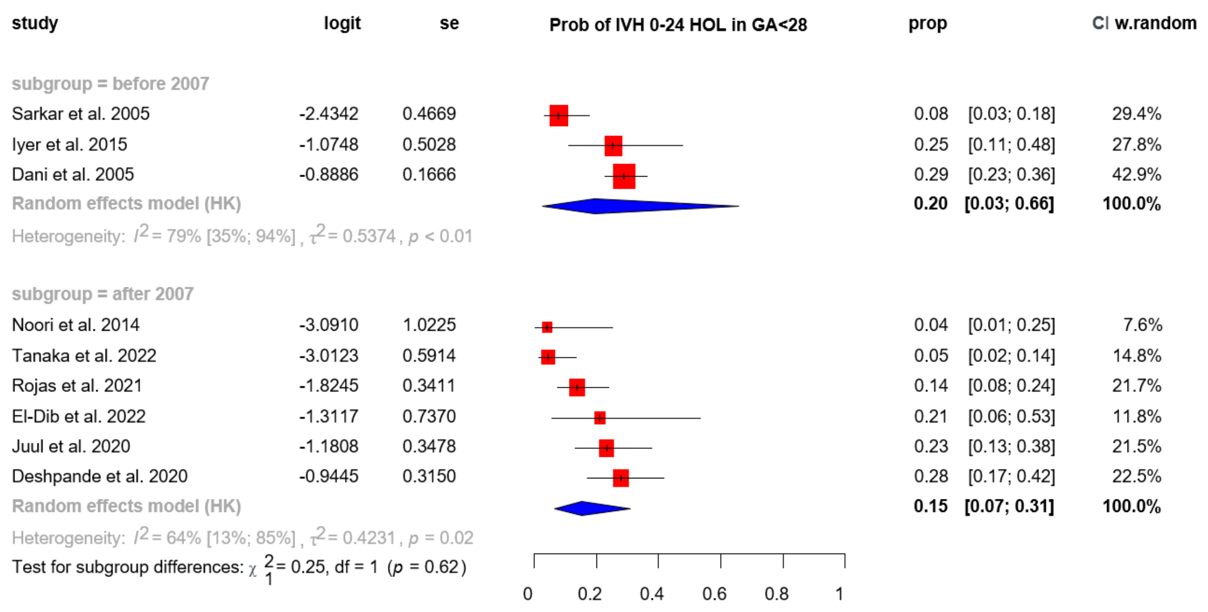

IVH, intraventricular hemorrhage; HOL, hours of life; GA: gestational age

eFigure 28. Overall IVH rate up to 48 HOL in GA<28 in the subgroups before and after 2007

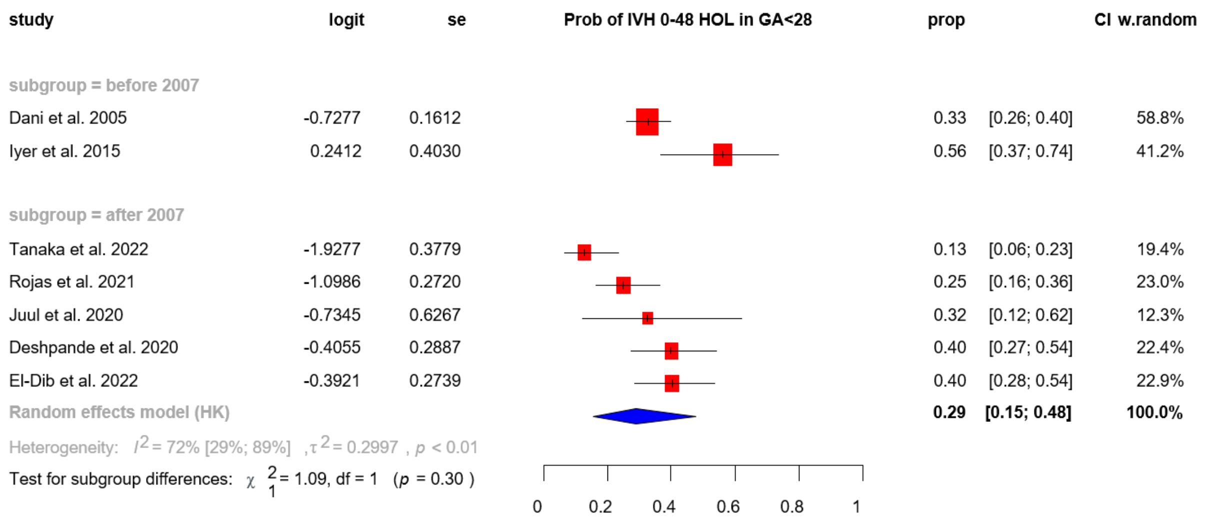

IVH, intraventricular hemorrhage; HOL, hours of life; GA: gestational age

© 2024 Nagy Zs et al. *JAMA Pediatrics.*

eFigure 29. Overall IVH rate up to 72 HOL in GA<28 in the subgroups before and after 2007

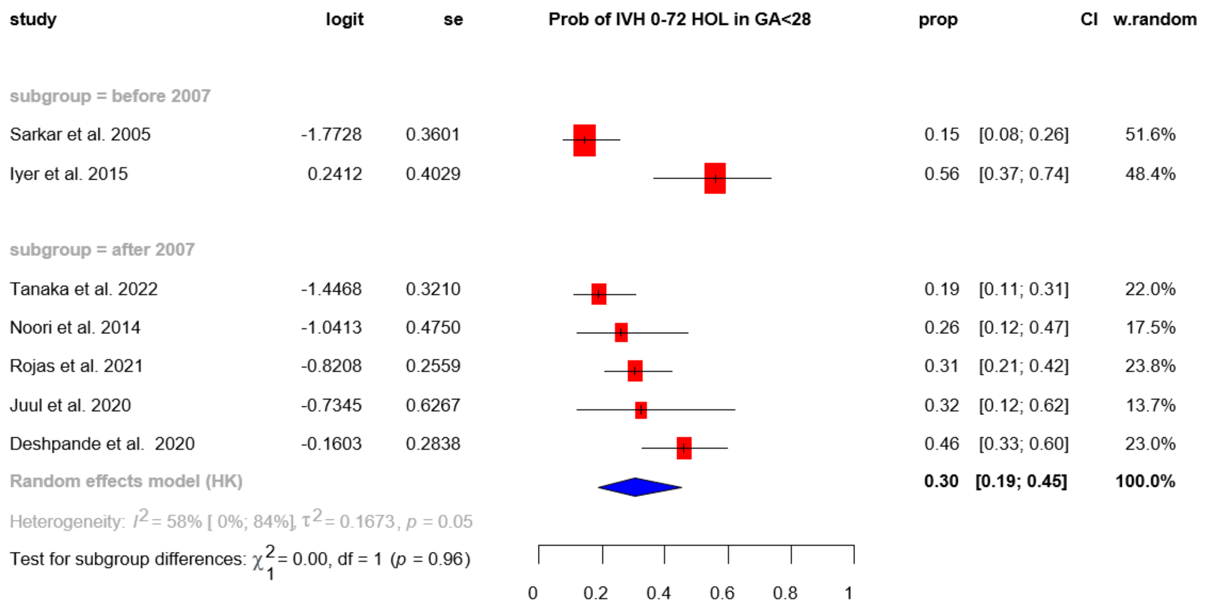

IVH, intraventricular hemorrhage; HOL, hours of life; GA: gestational age

© 2024 Nagy Zs et al. *JAMA Pediatrics.*

eFigure 30. IVH rate by before and after 2007 exclusion criteria of only congenital anomaly among preterm neonates

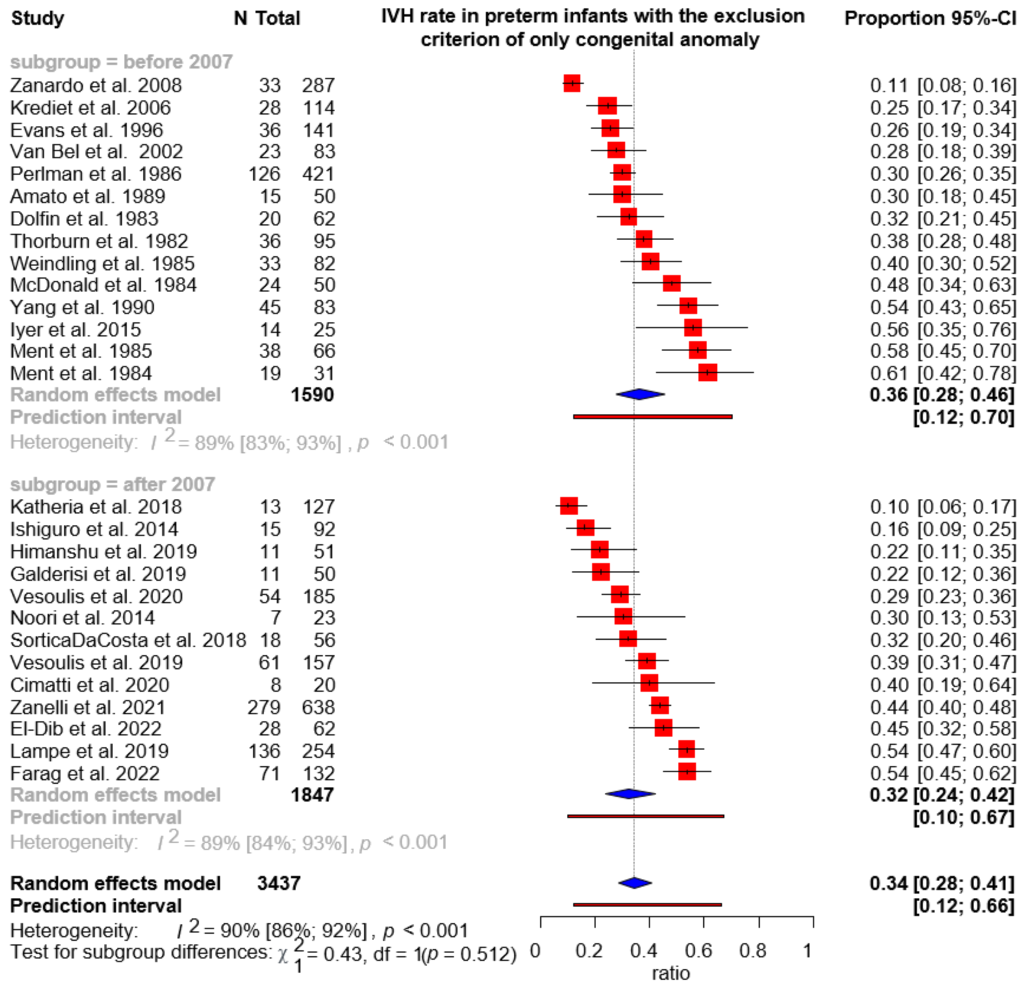

IVH, intraventricular hemorrhage; HOL, hours of life

© 2024 Nagy Zs et al. *JAMA Pediatrics.*

eFigure 31. IVH rate by before and after 2007 exclusion criteria of other than only congenital anomaly among preterm neonates

Study	N	Total	IVH prop. exclusion criteria of other than only cong. anomaly	Proportion	95%-CI
**subgroup1 = before 2007**					
Salafia et al., 1995	65	406		0.16	[0.13; 0.20]
Florio et al., 2006	11	53		0.21	[0.11; 0.34]
Kluckow et al., 2000	27	126		0.21	[0.15; 0.30]
Paradisis et al., 2009	25	93		0.27	[0.18; 0.37]
Ment et al., 94	138	504		0.27	[0.24; 0.31]
Sarkar et al., 2005	17	62		0.27	[0.17; 0.40]
Meek et al., 1998	7	24		0.29	[0.13; 0.51]
Osborn et al., 2003	38	128		0.30	[0.22; 0.38]
Chalak et al., 2011	9	30		0.30	[0.15; 0.49]
Dani et al., 2005	59	175		0.34	[0.27; 0.41]
Babnik et al., 2006	46	125		0.37	[0.28; 0.46]
Rumack et al., 1985	21	49		0.43	[0.29; 0.58]
Bada et al., 1989	68	145		0.47	[0.39; 0.55]
Szymonovicz et al., 1984	30	50		0.60	[0.45; 0.74]
Perlman et al., 1983	35	57		0.61	[0.48; 0.74]
Bada et al., 1984	122	155		0.79	[0.71; 0.85]
**Random effects model**		**2182**		**0.36**	**[0.27; 0.46]**
**Prediction interval**					**[0.10; 0.74]**
Heterogeneity: $I^2 = 93\%$ [91%; 95%], $p < 0.001$					
**subgroup2 = after 2007**					
Ikeda et al., 2014	8	81		0.10	[0.04; 0.19]
Schreiner et al., 2021	80	536		0.15	[0.12; 0.18]
Kalani et al., 2016	14	93		0.15	[0.08; 0.24]
Tanaka et al., 2022	13	63		0.21	[0.11; 0.33]
Martini et al., 2022	16	77		0.21	[0.12; 0.32]
Duppré et al., 2015	55	250		0.22	[0.17; 0.28]
Alderliesten et al., 2013	148	652		0.23	[0.20; 0.26]
Thanhaeuser et al., 2018	33	128		0.26	[0.18; 0.34]
Najib et al., 2018	19	67		0.28	[0.18; 0.41]
Katheria et al., 2014	19	60		0.32	[0.20; 0.45]
Shan et al., 2023	82	239		0.34	[0.28; 0.41]
June et al., 2021	36	99		0.36	[0.27; 0.47]
Juul et al., 2020	344	903		0.38	[0.35; 0.41]
Bates et al., 2015	46	108		0.43	[0.33; 0.52]
Deshpande et al., 2020	26	50		0.52	[0.37; 0.66]
Jiang et al., 2023	49	92		0.53	[0.43; 0.64]
Rojas et al., 2021	42	72		0.58	[0.46; 0.70]
**Random effects model**		**3570**		**0.29**	**[0.23; 0.37]**
**Prediction interval**					**[0.09; 0.63]**
Heterogeneity: $I^2 = 92\%$ [89%; 94%], $p < 0.001$					
**Random effects model**		**5752**		**0.32**	**[0.27; 0.38]**
**Prediction interval**					**[0.10; 0.67]**
Heterogeneity: $I^2 = 93\%$ [91%; 94%], $p < 0.001$					
Test for subgroup differences: $\chi^2_1 = 1.39$, df = 1 ($p = 0.238$)					

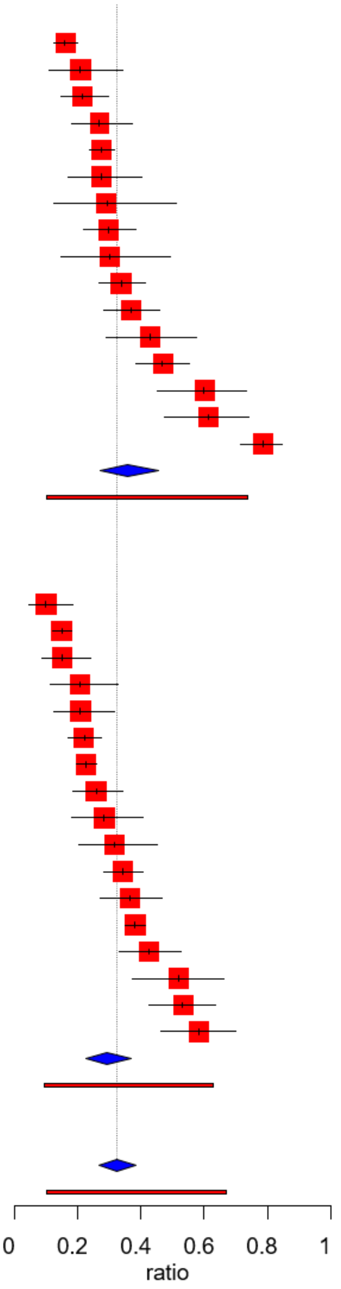

IVH, intraventricular hemorrhage; HOL, hours of life

© 2024 Nagy Zs et al. *JAMA Pediatrics.*

eFigure 32. sIVH rate by exclusion criteria of only congenital anomaly among preterm neonates in the subgroups before and after 2007

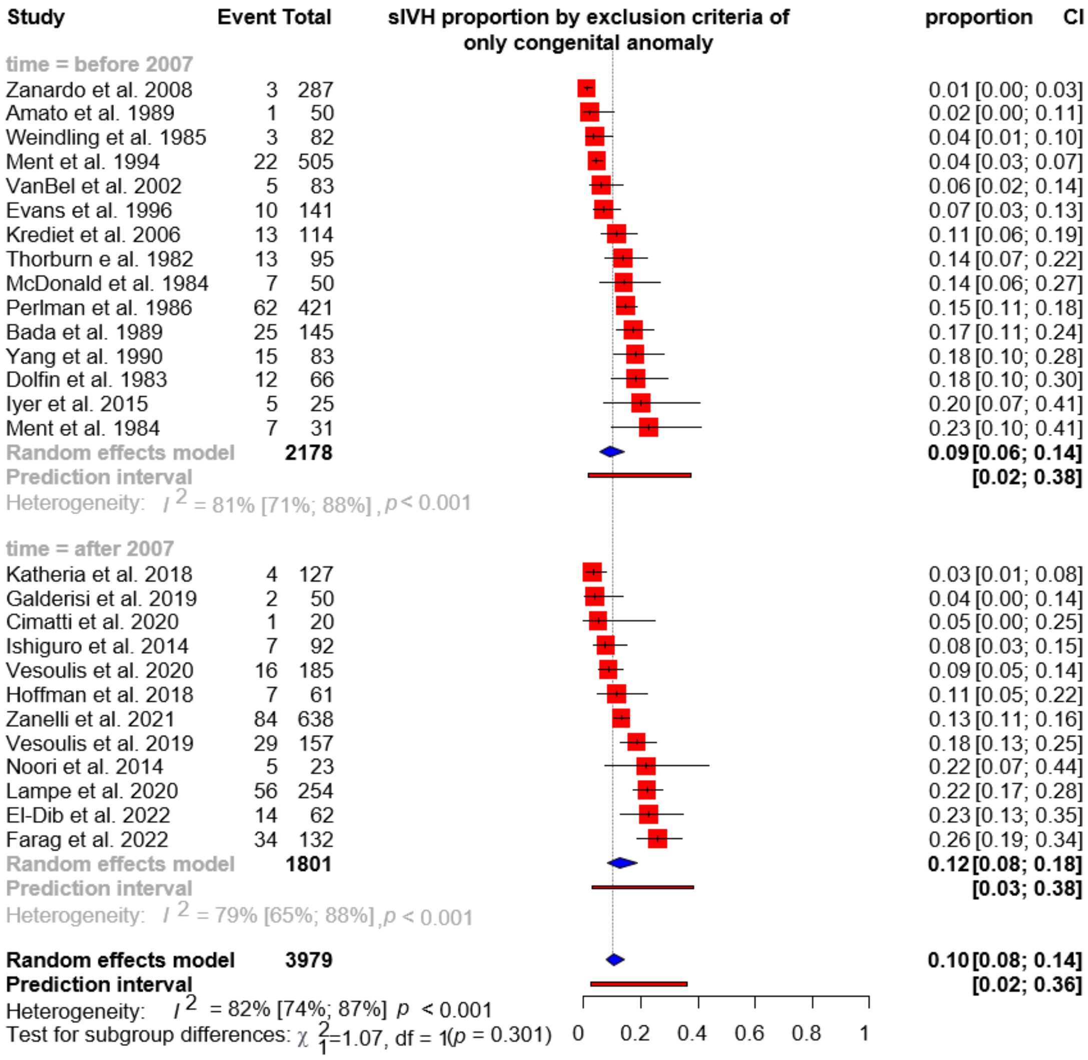

sIVH, severe intraventricular hemorrhage; HOL, hours of life, CI: Confidence Intervals

© 2024 Nagy Zs et al. *JAMA Pediatrics.*

eFigure 33. sIVH rate by exclusion criteria of other than only congenital anomaly among preterm neonates in the subgroups before and after 2007

Study	Event	Total	proportion	CI
subgroup1 = before 2007				
Florio et al., 2006	0	53	0.00	[0.00; 0.07]
Ment et al., 1994	22	505	0.04	[0.03; 0.07]
Salafia et al., 1995	23	406	0.06	[0.04; 0.08]
Kluckow et al., 2000	13	126	0.10	[0.06; 0.17]
Paradisis et al., 2006	3	29	0.10	[0.02; 0.27]
Bada et al., 1984	19	155	0.12	[0.08; 0.18]
Osborn et al., 2003	17	128	0.13	[0.08; 0.20]
Rumack et al., 1985	7	49	0.14	[0.06; 0.27]
Babnik et al., 2006	20	125	0.16	[0.10; 0.24]
Meek et al., 1998	5	24	0.21	[0.07; 0.42]
Chalak et al., 2011	7	30	0.23	[0.10; 0.42]
Random effects model		**1630**	**0.10**	**[0.06; 0.15]**
Prediction interval				**[0.03; 0.31]**
Heterogeneity: $I^2 = 76\%$ [57%; 87%], $p < 0.001$				
subgroup2 = after 2007				
Ikeda et al., 2014	1	81	0.01	[0.00; 0.07]
Schreiner et al., 2021	16	536	0.03	[0.02; 0.05]
Alderliesten et al., 2013	35	650	0.05	[0.04; 0.07]
Martini et al., 2022	5	77	0.06	[0.02; 0.15]
Deshpande et al., 2020	4	50	0.08	[0.02; 0.19]
Jiang et al., 2023	8	92	0.09	[0.04; 0.16]
Duppré et al., 2015	25	250	0.10	[0.07; 0.14]
Katheria et al., 2014	6	60	0.10	[0.04; 0.21]
Shan et al., 2023	28	238	0.12	[0.08; 0.17]
Juul et al., 2020	107	906	0.12	[0.10; 0.14]
Da Costa et al., 2018	9	56	0.16	[0.08; 0.28]
Bates et al., 2015	22	108	0.20	[0.13; 0.29]
June et al., 2021	21	99	0.21	[0.14; 0.31]
Naoshi et al., 2021	55	216	0.25	[0.20; 0.32]
Random effects model		**3419**	**0.10**	**[0.06; 0.14]**
Prediction interval				**[0.02; 0.34]**
Heterogeneity: $I^2 = 89\%$ [84%; 93%], $p < 0.001$				
**Random effects model**		**5049**	**0.10**	**[0.07; 0.13]**
**Prediction interval**				**[0.03; 0.30]**

Heterogeneity: $I^2 = 85\%$ [80%; 90%], $p < 0.001$
Test for subgroup differences: $\chi^2_1 = 0.00$, df = 1 ($p = 0.944$)

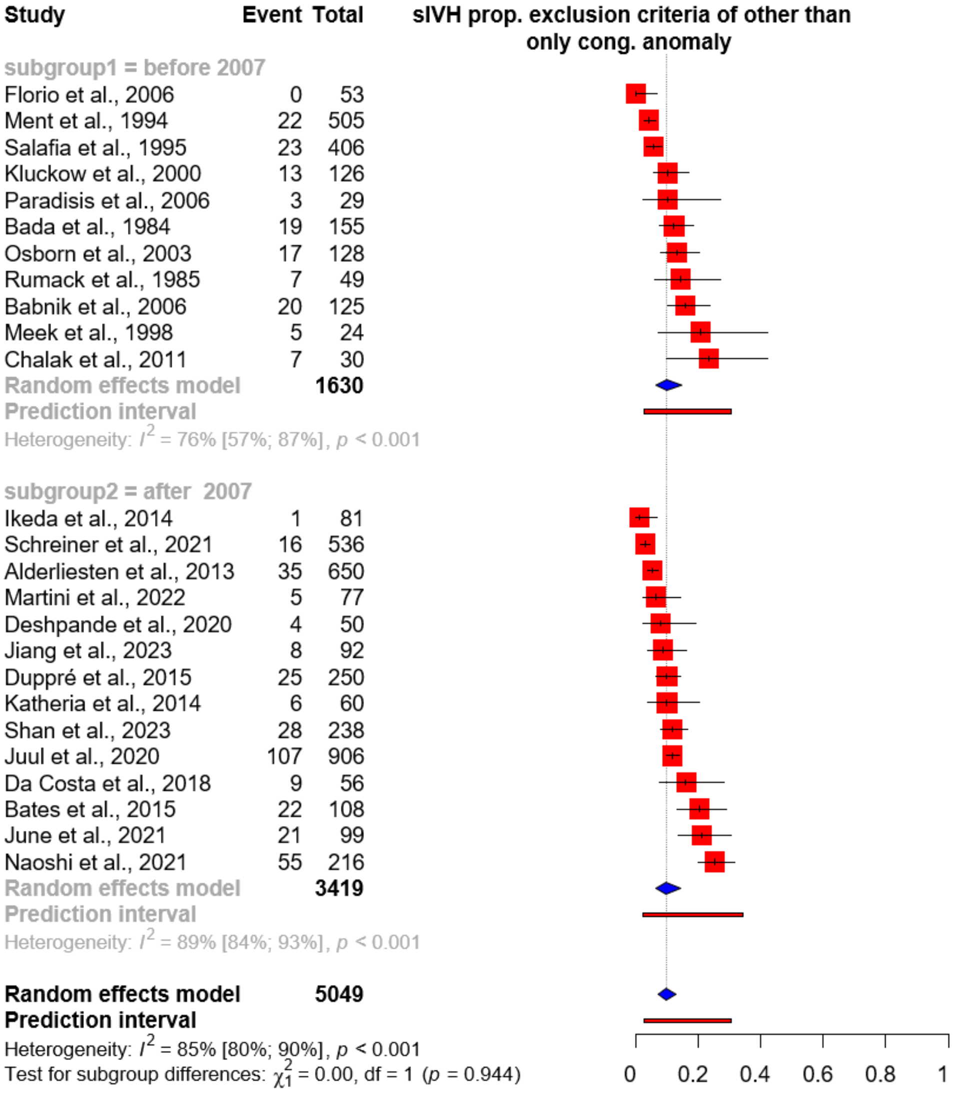

sIVH, severe intraventricular hemorrhage; HOL, hours of life, CI: Confidence Intervals

© 2024 Nagy Zs et al. *JAMA Pediatrics.*

eFigure 34. Antenatal steroid rate in the subgroups before and after 2007

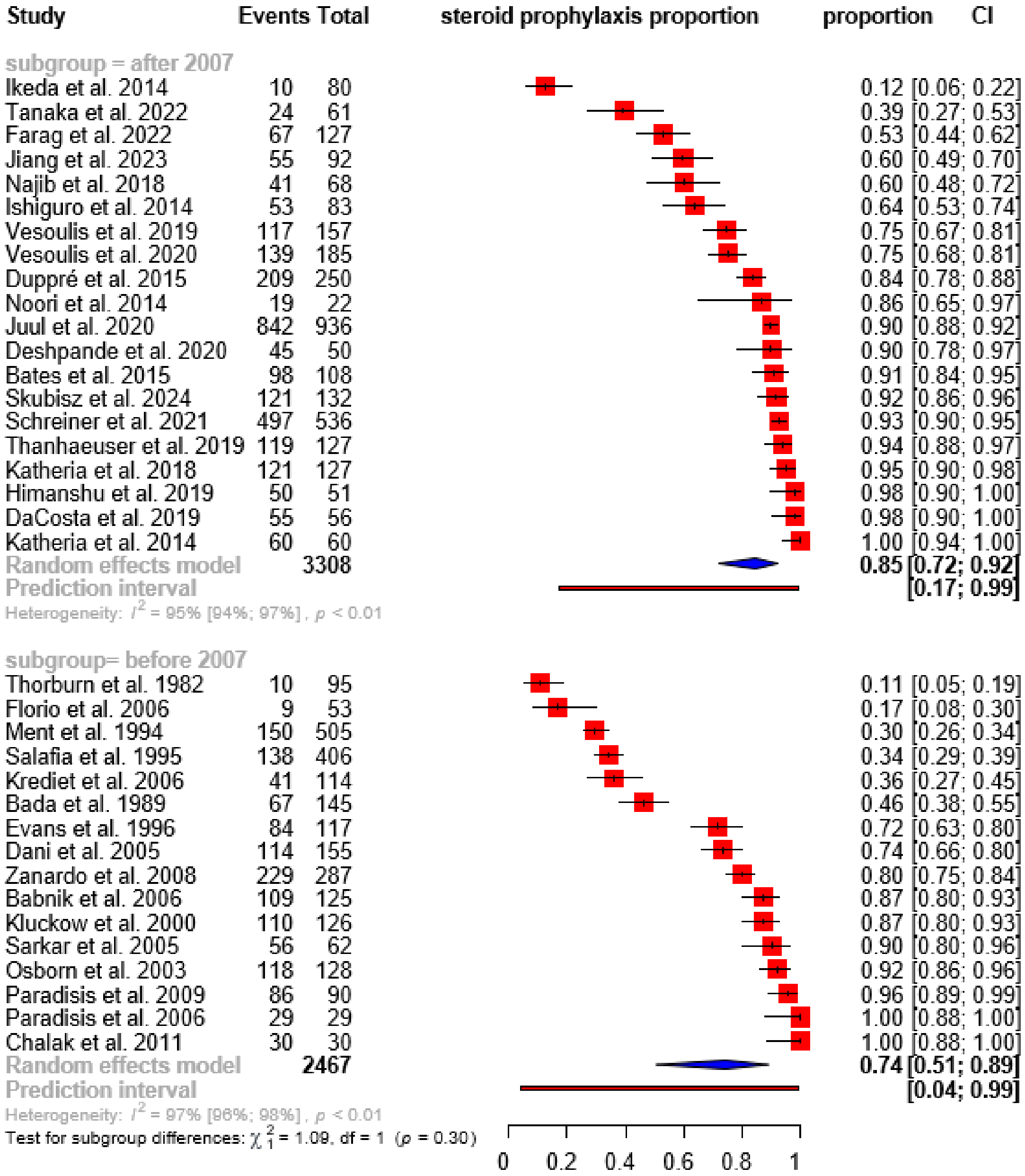

CI: Confidence Intervals

© 2024 Nagy Zs et al. *JAMA Pediatrics.*

eFigure 35. Outborn rate in the subgroups before and after 2007

Study	Events	Total	proportion	CI
**subgroup = after 2007**				
Lampe et al 2020	0	254	0.00	[0.00; 0.01]
Duppré et al. 2015	0	251	0.00	[0.00; 0.01]
Najib et al. 2018	0	140	0.00	[0.00; 0.03]
Jiang et al. 2023	0	102	0.00	[0.00; 0.04]
Ikeda et al. 2014	0	81	0.00	[0.00; 0.04]
Tanaka et al. 2022	0	64	0.00	[0.00; 0.06]
Katheria et al. 2014	0	60	0.00	[0.00; 0.06]
Deshpande et al. 2020	1	51	0.02	[0.00; 0.10]
June et al. 2021	4	99	0.04	[0.01; 0.10]
SorticaDaCosta et al. 2018	4	56	0.07	[0.02; 0.17]
Skubisz et al. 2024	12	132	0.09	[0.05; 0.15]
Zanelli et al. 2021	108	638	0.17	[0.14; 0.20]
Bates et al. 2015	24	119	0.20	[0.13; 0.29]
**Random effects model**		**2047**	**0.01**	**[0.00; 0.06]**
**Prediction interval**				**[0.00; 0.80]**
Heterogeneity: $I^2 = 48\%$ [0%; 72%], $p = 0.03$				
**subgroup = before 2007**				
Bada et al. 1984	0	155	0.00	[0.00; 0.02]
Bada et al. 1989	0	145	0.00	[0.00; 0.03]
Babnik et al. 2006	0	125	0.00	[0.00; 0.03]
Paradisis et al. 2009	0	93	0.00	[0.00; 0.04]
Yang et al. 1990	0	92	0.00	[0.00; 0.04]
Dolfin et al. 1983	0	66	0.00	[0.00; 0.05]
McDonald et al. 1984	0	50	0.00	[0.00; 0.07]
Chalak et al. 2011	0	30	0.00	[0.00; 0.12]
Paradisis et al. 2006	0	29	0.00	[0.00; 0.12]
Szymonovicz et al. 1984	4	56	0.07	[0.02; 0.17]
Kluckow et al. 2000	12	128	0.09	[0.05; 0.16]
Ment et al. 1994	79	466	0.17	[0.14; 0.21]
Weindling et al. 1985	17	82	0.21	[0.13; 0.31]
Perlman et al 1983	23	50	0.46	[0.32; 0.61]
Thorburn et al. 1982	55	95	0.58	[0.47; 0.68]
**Random effects model**		**1662**	**0.01**	**[0.00; 0.09]**
**Prediction interval**				**[0.00; 0.97]**
Heterogeneity: $I^2 = 85\%$ [78%; 91%], $p < 0.01$				
Test for subgroup differences: $\chi^2_1 = 0.00$, df = 1 ($p = 0.97$)				

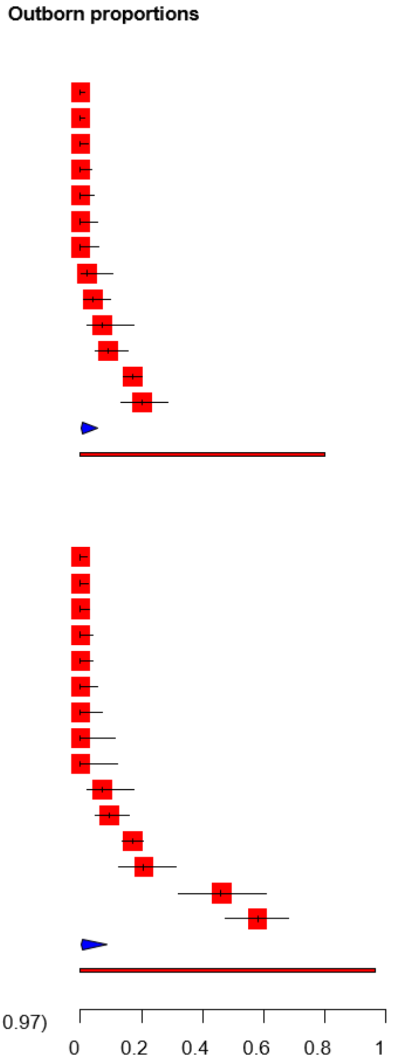

CI: Confidence Intervals

© 2024 Nagy Zs et al. *JAMA Pediatrics.*

eFigure 36. The pooled mean gestational age within subgroups with study data periods before and after 2007.

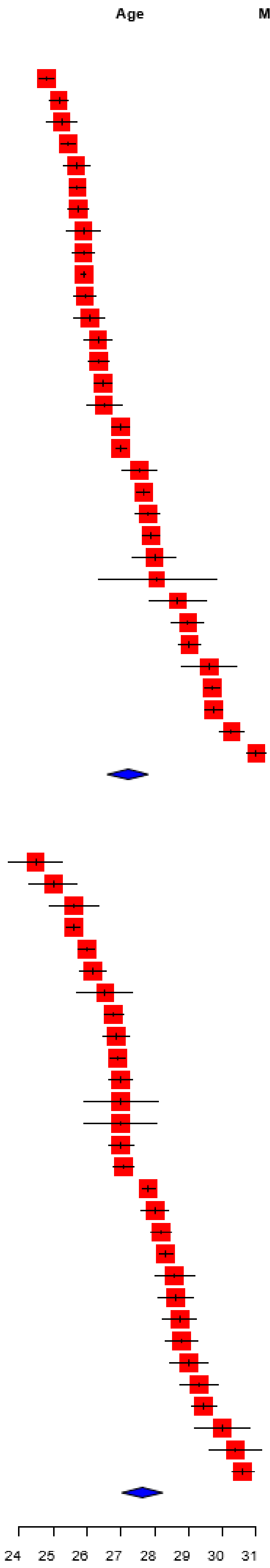

Study	Total	Mean	SD	MRAW	95%-CI
subgroup = after 2007					
Naoshi et al. 2021	216	24.80	1.47	24.80	[24.60; 25.00]
Vesoulis et al. 2019	157	25.18	1.83	25.18	[24.90; 25.47]
Tanaka et al. 2022	61	25.26	1.85	25.26	[24.79; 25.72]
Bates et al. 2015	108	25.44	1.17	25.44	[25.22; 25.67]
El-Dib et al. 2022	62	25.69	1.55	25.69	[25.30; 26.07]
Rojas et al. 2021	72	25.72	1.00	25.72	[25.49; 25.95]
Deshpande et al. 2020	50	25.75	1.07	25.75	[25.45; 26.04]
Noori et al. 2014	22	25.90	1.18	25.90	[25.40; 26.39]
June et al. 2021	99	25.90	1.60	25.90	[25.58; 26.22]
Juul et al. 2020	936	25.90	1.16	25.90	[25.83; 25.98]
Thanhaeuser et al. 2019	127	25.95	1.94	25.95	[25.61; 26.28]
SorticaDaCosta et al. 2018	56	26.08	1.68	26.08	[25.64; 26.52]
Hoffman et al. 2018	61	26.32	1.63	26.32	[25.91; 26.73]
Vesoulis et al. 2020	185	26.35	2.00	26.35	[26.06; 26.64]
Lampe et al. 2020	254	26.49	2.10	26.49	[26.23; 26.74]
Ikeda et al. 2014	80	26.51	2.36	26.51	[25.99; 27.03]
Himanshu et al. 2019	51	27.00	0.99	27.00	[26.73; 27.27]
Zanelli et al. 2021	638	27.00	2.00	27.00	[26.84; 27.16]
Ishiguro et al. 2014	83	27.55	2.30	27.55	[27.06; 28.05]
Shan et al. 2023	238	27.66	1.51	27.66	[27.47; 27.85]
Katheria et al. 2018	127	27.80	2.08	27.80	[27.43; 28.16]
Alderliesten et al. 2013	90	27.89	1.29	27.89	[27.62; 28.15]
Katheria et al. 2014	60	28.00	2.53	28.00	[27.36; 28.64]
Cimatti et al. 2020	20	28.06	3.97	28.06	[26.32; 29.80]
Martini et al. 2022	77	28.68	3.70	28.68	[27.86; 29.51]
Jiang et al. 2023	92	28.97	2.29	28.97	[28.51; 29.44]
Duppré et al. 2015	250	29.02	2.60	29.02	[28.69; 29.34]
Galderisi et al. 2019	50	29.60	2.87	29.60	[28.81; 30.40]
Schreiner et al. 2021	536	29.70	2.31	29.70	[29.50; 29.89]
Farag et al. 2022	127	29.74	1.59	29.74	[29.46; 30.02]
Kalani et al. 2016	93	30.27	1.70	30.27	[29.92; 30.62]
Najib et al.2018	68	31.00	1.27	31.00	[30.70; 31.30]
Random effects model	**5146**			**27.21**	**[26.61; 27.82]**
Heterogeneity: I^2 = 99% [99%; 99%], τ^2 = 2.7871, p = 0					
subgroup = before 2007					
Iyer et al. 2015	25	24.48	2.00	24.48	[23.70; 25.26]
Chalak et al. 2011	30	25.00	2.00	25.00	[24.28; 25.72]
Paradisis et al. 2006	29	25.60	2.00	25.60	[24.87; 26.33]
Dani et al. 2005	155	25.60	1.19	25.60	[25.41; 25.79]
Paradisis et al. 2009	90	26.00	1.16	26.00	[25.76; 26.24]
Sarkar et al. 2005	62	26.17	1.62	26.17	[25.76; 26.57]
Meek et al. 1998	24	26.54	2.04	26.54	[25.72; 27.36]
Zanardo et al. 2008	287	26.80	2.50	26.80	[26.51; 27.09]
Osborn et al. 2003	128	26.85	2.23	26.85	[26.47; 27.24]
Salafia et al. 1995	406	26.90	2.30	26.90	[26.68; 27.12]
Kluckow et al. 2000	126	27.00	2.00	27.00	[26.65; 27.35]
Szymonovicz et al. 1984	50	27.00	4.00	27.00	[25.89; 28.11]
Thorburn et al. 1982	95	27.00	5.29	27.00	[25.94; 28.06]
Evans et al. 1996	117	27.00	2.00	27.00	[26.64; 27.36]
Babnik et al. 2006	125	27.08	1.63	27.08	[26.79; 27.36]
Ment et al. 1994	505	27.81	2.07	27.81	[27.63; 27.99]
Bada et al. 1989	145	28.00	2.40	28.00	[27.61; 28.39]
VanBel et al. 2002	83	28.20	1.40	28.20	[27.90; 28.50]
Florio et al. 2006	53	28.33	0.67	28.33	[28.15; 28.51]
Ment et al. 1985	48	28.60	2.05	28.60	[28.02; 29.18]
McDonald et al. 1984	50	28.62	1.85	28.62	[28.11; 29.13]
Weindling et al. 1985	82	28.73	2.30	28.73	[28.23; 29.23]
Dolfin et al. 1983	60	28.80	1.79	28.80	[28.35; 29.25]
Rumack et al. 1985	49	29.00	2.00	29.00	[28.44; 29.56]
Yang et al. 1990	83	29.30	2.55	29.30	[28.75; 29.85]
Krediet et al. 2006	114	29.45	1.89	29.45	[29.11; 29.80]
Perlman et al. 1983	50	30.00	3.00	30.00	[29.17; 30.83]
Amato et al. 1989	50	30.37	2.83	30.37	[29.59; 31.15]
Bada et al. 1984	155	30.60	2.00	30.60	[30.29; 30.91]
Random effects model	**3276**			**27.62**	**[27.02; 28.22]**
Heterogeneity: I^2 = 98% [98%; 99%], τ^2 = 2.3918, p = 0					
Test for subgroup differences: χ^2_1 = 0.95, df = 1 (p = 0.33)					

© 2024 Nagy Zs et al. *JAMA Pediatrics.*

eFigure 37. Early study mortality in the subgroups before and after 2007

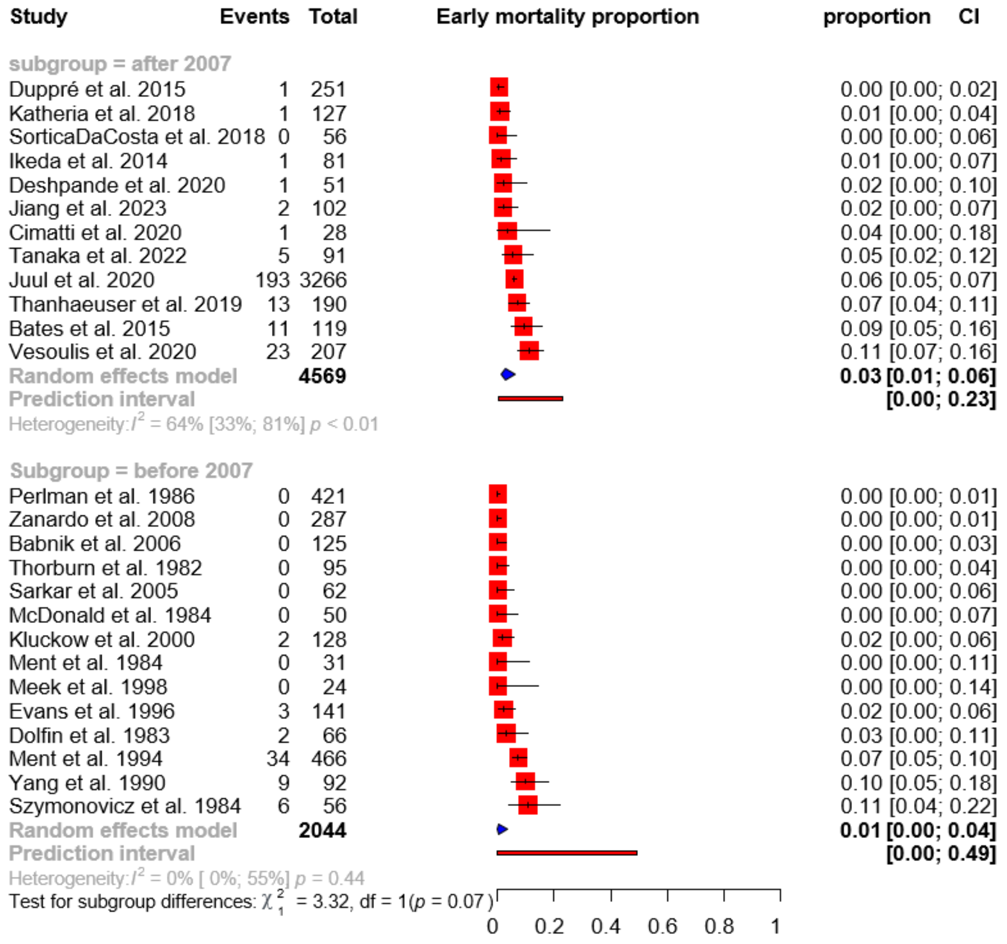

© 2024 Nagy Zs et al. *JAMA Pediatrics.*

eFigure 38. Total mortality in the subgroups before and after 2007

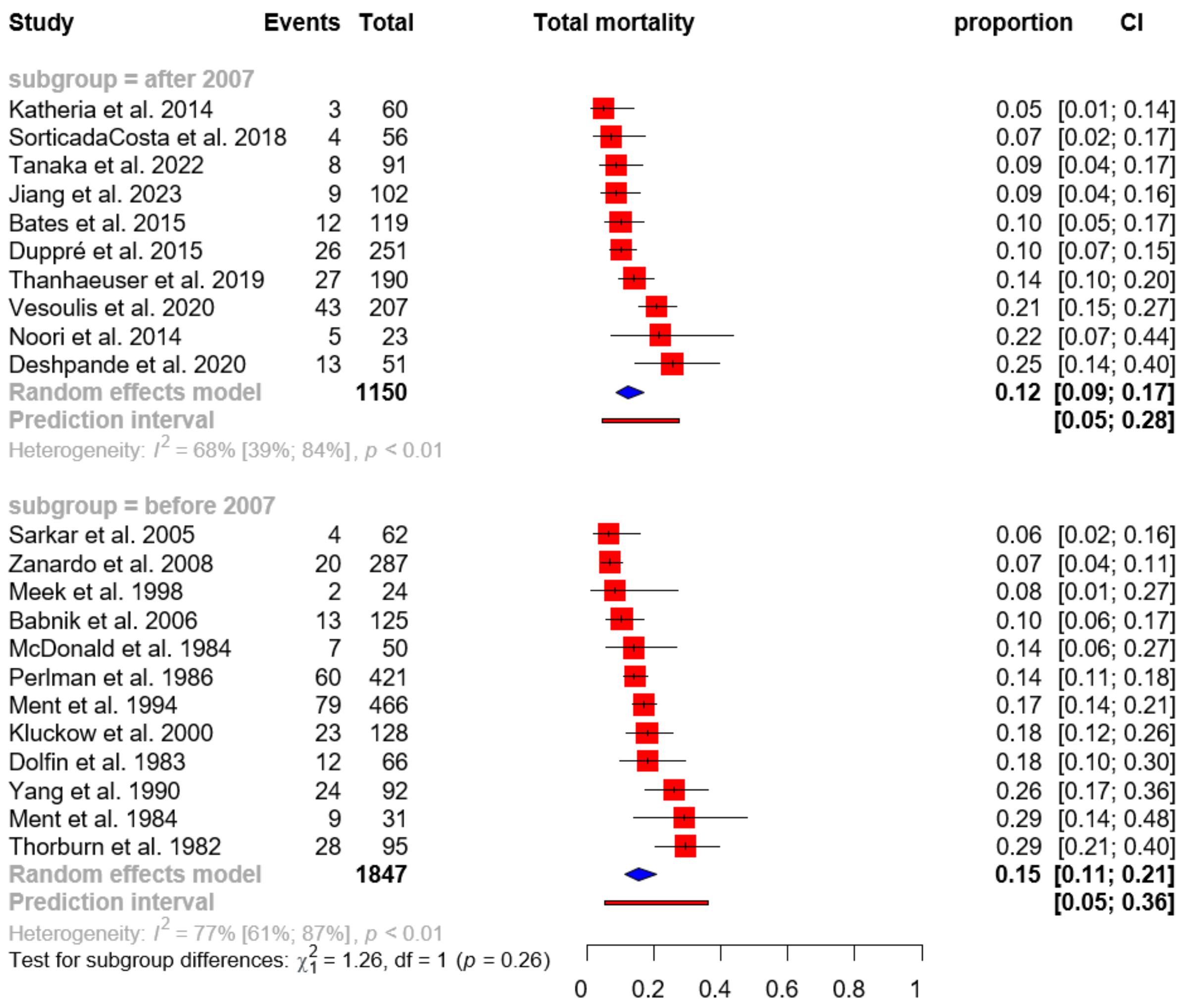

© 2024 Nagy Zs et al. *JAMA Pediatrics.*

eFigure 39. Funnel plot of the overall IVH in the subgroup before 2007

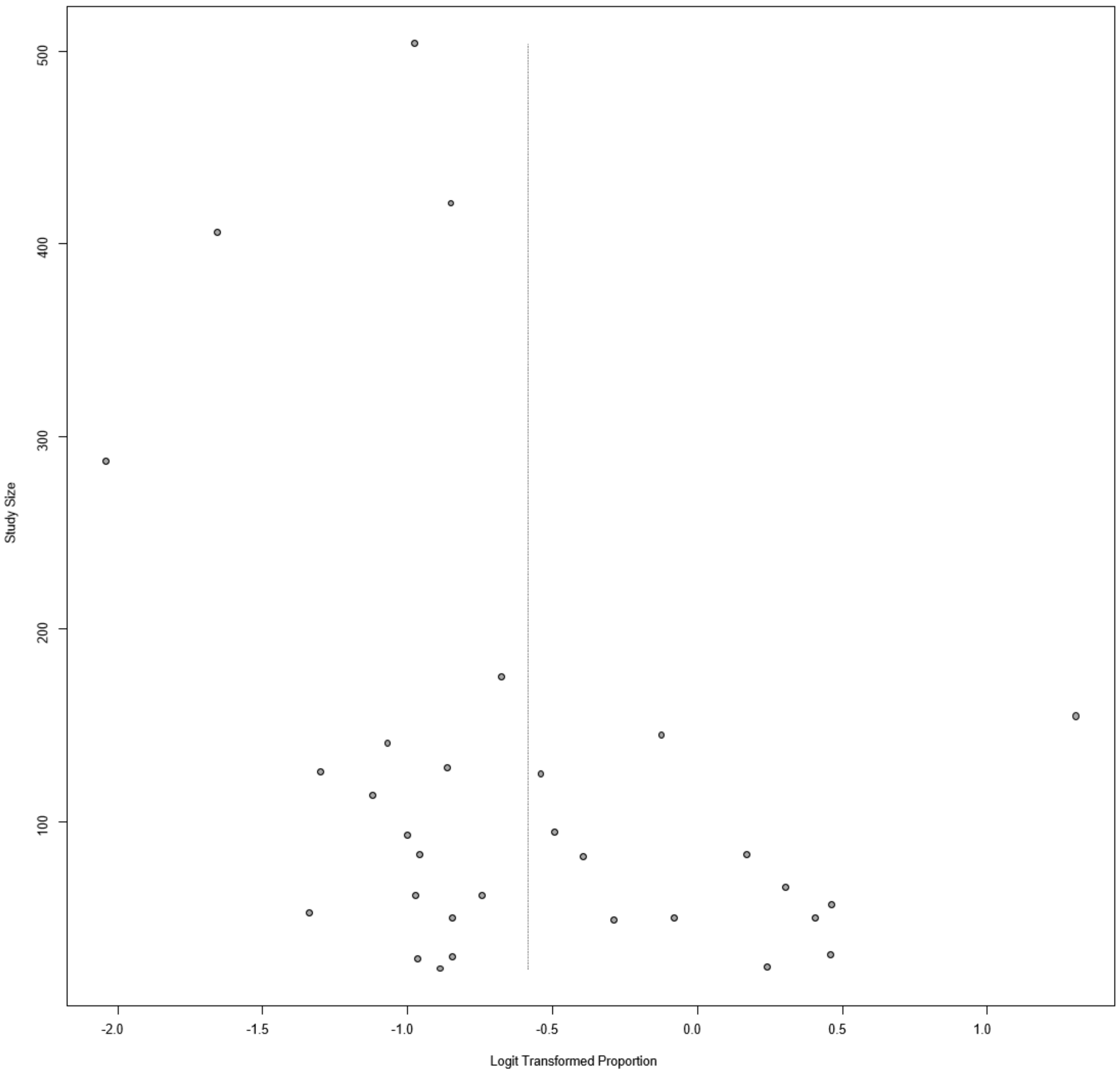

© 2024 Nagy Zs et al. *JAMA Pediatrics.*

eFigure 40. Funnel plot of the overall IVH in the subgroup after 2007

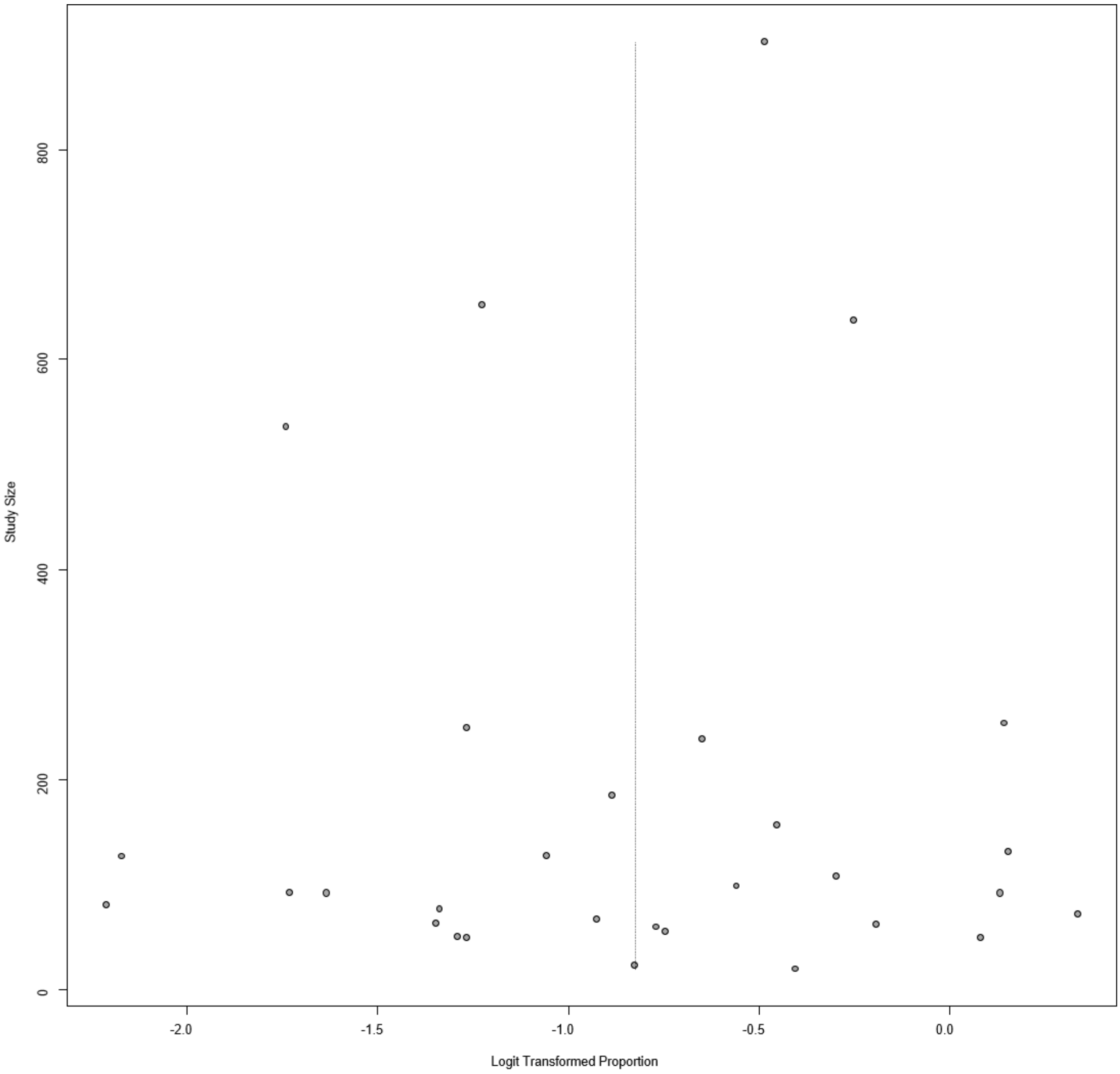

eFigure 41. Peter's test in the subgroup before 2007:

Linear regression test of funnel plot asymmetry

Test result: t = 2.13, df = 29, p-value = 0.0419

eFigure 42. Peter's test in the subgroup after 2007:

Linear regression test of funnel plot asymmetry

Test result: t = 0.03, df = 28, p-value = 0.9725

© 2024 Nagy Zs et al. *JAMA Pediatrics.*

Sample estimates:

```
   bias se.bias intercept se.intercept
 0.5960 17.1528   -0.6796       0.1442
```

Details:

- multiplicative residual heterogeneity variance (tau^2 = 13.1356)
- predictor: inverse of total sample size
- weight: inverse variance of average event probability
- reference: Peters et al. (2006), JAMA

© 2024 Nagy Zs et al. *JAMA Pediatrics.*

eREFERENCES

1. Bogaerts K, Komárek A, Lesaffre E. *Survival Analysis with Interval-Censored Data*. Chapman and Hall/CRC; 2017. doi:10.1201/9781315116945

2. Stijnen T, Hamza TH, Özdemir P. Random effects meta-analysis of event outcome in the framework of the generalized linear mixed model with applications in sparse data. *Stat Med*. 2010;29(29):3046-3067. doi:10.1002/sim.4040

3. Julian PT Higgins, Sally Green. *Cochrane Handbook for Systematic Reviews of Interventions Version Version 5.1.0 [Updated March 2011] Chapter 7./Table 7. 7. a. Formulae for Combining Groups*.

4. Alderliesten T, Lemmers PMA, Smarius JJM, van de Vosse RE, Baerts W, van Bel F. Cerebral Oxygenation, Extraction, and Autoregulation in Very Preterm Infants Who Develop Peri-Intraventricular Hemorrhage. *J Pediatr*. 2013;162(4):698-704.e2. doi:10.1016/j.jpeds.2012.09.038

5. Noori S, McCoy M, Anderson MP, Ramji F, Seri I. Changes in Cardiac Function and Cerebral Blood Flow in Relation to Peri/Intraventricular Hemorrhage in Extremely Preterm Infants. *J Pediatr*. 2014;164(2):264-270.e3. doi:10.1016/j.jpeds.2013.09.045

6. Sarkar S, Kaplan C, Wiswell TE, Spitzer AR. Histological Chorioamnionitis and the Risk of Early Intraventricular Hemorrhage in Infants Born ≤28 Weeks Gestation. *Journal of Perinatology*. 2005;25(12):749-752. doi:10.1038/sj.jp.7211399

7. Krediet TG, Kavelaars A, Vreman HJ, Heijnen CJ, van Bel F. Respiratory distress syndrome-associated inflammation is related to early but not late peri/intraventricular hemorrhage in preterm infants. *J Pediatr*. 2006;148(6):740-746. doi:10.1016/j.jpeds.2006.01.037

8. Cimatti AG, Martini S, Galletti S, et al. Cerebral Oxygenation and Autoregulation in Very Preterm Infants Developing IVH During the Transitional Period: A Pilot Study. *Front Pediatr*. 2020;8. doi:10.3389/fped.2020.00381

9. Deshpande P, Jain A, Ibarra Ríos D, et al. Combined Multimodal Cerebral Monitoring and Focused Hemodynamic Assessment in the First 72 h in Extremely Low Gestational Age Infants. *Neonatology*. 2020;117(4):504-512. doi:10.1159/000508961

© 2024 Nagy Zs et al. *JAMA Pediatrics.*

10. El-Dib M, Munster C, Sunwoo J, et al. Association of early cerebral oxygen saturation and brain injury in extremely preterm infants. *Journal of Perinatology*. 2022;42(10):1385-1391. doi:10.1038/s41372-022-01447-w

11. Babnik J, Stucin-Gantar I, Kornhauser-Cerar L, Sinkovec J, Wraber B, Derganc M. Intrauterine Inflammation and the Onset of Peri-Intraventricular Hemorrhage in Premature Infants. *Neonatology*. 2006;90(2):113-121. doi:10.1159/000092070

12. Bada HS, Korones SB, Anderson GD, Magill HL, Wong SP. Obstetric factors and relative risk of neonatal germinal layer/intraventricular hemorrhage. *Am J Obstet Gynecol*. 1984;148(6):798-804. doi:10.1016/0002-9378(84)90571-4

13. Chalak LF, Sikes NC, Mason MJ, Kaiser JR. Low-Voltage aEEG as Predictor of Intracranial Hemorrhage in Preterm Infants. *Pediatr Neurol*. 2011;44(5):364-369. doi:10.1016/j.pediatrneurol.2010.11.018

14. Dani C, Bertini G, Pezzati M, et al. Prophylactic Ibuprofen for the Prevention of Intraventricular Hemorrhage Among Preterm Infants: A Multicenter, Randomized Study. *Pediatrics*. 2005;115(6):1529-1535. doi:10.1542/peds.2004-1178

15. Dolfin T, Skidmore MB, Fong KW, Hoskins EM, Shennan AT. Incidence, severity, and timing of subependymal and intraventricular hemorrhages in preterm infants born in a perinatal unit as detected by serial real-time ultrasound. *Pediatrics*. 1983;71(4):541-546.

16. Farag MM, Gouda MH, Almohsen AMA, Khalifa MA. Intraventricular hemorrhage prediction in premature neonates in the era of hemodynamics monitoring: a prospective cohort study. *Eur J Pediatr*. 2022;181(12):4067-4077. doi:10.1007/s00431-022-04630-5

17. Ikeda T, Amizuka T, Ito Y, et al. Changes in the perfusion waveform of the internal cerebral vein and intraventricular hemorrhage in the acute management of extremely low-birth-weight infants. *Eur J Pediatr*. 2015;174(3):331-338. doi:10.1007/s00431-014-2396-1

18. Ishiguro A, Suzuki K, Sekine T, et al. Skin blood flow as a predictor of intraventricular hemorrhage in very-low-birth-weight infants. *Pediatr Res*. 2014;75(2):322-327. doi:10.1038/pr.2013.215

© 2024 Nagy Zs et al. *JAMA Pediatrics.*

19. Katheria AC, Leone TA, Woelkers D, Garey DM, Rich W, Finer NN. The Effects of Umbilical Cord Milking on Hemodynamics and Neonatal Outcomes in Premature Neonates. *J Pediatr*. 2014;164(5):1045-1050.e1. doi:10.1016/j.jpeds.2014.01.024

20. Katheria AC, Harbert MJ, Nagaraj SB, et al. The Neu-Prem Trial: Neuromonitoring of Brains of Infants Born Preterm During Resuscitation—A Prospective Observational Cohort Study. *J Pediatr*. 2018;198:209-213.e3. doi:10.1016/j.jpeds.2018.02.065

21. Meek JH, Tyszczuk L, Elwell CE, Wyatt JS. Low cerebral blood flow is a risk factor for severe intraventricular haemorrhage. *Arch Dis Child Fetal Neonatal Ed*. 1999;81(1):F15-F18. doi:10.1136/fn.81.1.F15

22. Kluckow M. Low superior vena cava flow and intraventricular haemorrhage in preterm infants. *Arch Dis Child Fetal Neonatal Ed*. 2000;82(3):188F - 194. doi:10.1136/fn.82.3.F188

23. McDonald MM, Koops BL, Johnson ML, et al. Timing and Antecedents of Intracranial Hemorrhage in the Newborn. *Pediatrics*. 1984;74(1):32-36. doi:10.1542/peds.74.1.32

24. Ment LR, Duncan CC, Ehrenkranz RA, et al. Intraventricular hemorrhage in the preterm neonate: Timing and cerebral blood flow changes. *J Pediatr*. 1984;104(3):419-425. doi:10.1016/S0022-3476(84)81109-9

25. Ment LR, Oh W, Ehrenkranz RA, Philip AGS, Duncan CC, Makuch RW. Antenatal steroids, delivery mode, and intraventricular hemorrhage in preterm infants. *Am J Obstet Gynecol*. 1995;172(3):795-800. doi:10.1016/0002-9378(95)90001-2

26. Perlman JM. Intraventricular Hemorrhage in Extremely Small Premature Infants. *Arch Pediatr Adolesc Med*. 1986;140(11):1122. doi:10.1001/archpedi.1986.02140250048034

27. Amato M, Hüppi P, Gambon R, Schneider H. Biochemical timing of peri-intraventricular hemorrhage assessed by perinatal CPK-BB isoenzyme measurements. *jpme*. 1989;17(6):447-452. doi:10.1515/jpme.1989.17.6.447

28. Duppré P, Sauer H, Giannopoulou EZ, et al. Cellular and humoral coagulation profiles and occurrence of IVH in VLBW and ELWB infants. *Early Hum Dev*. 2015;91(12):695-700. doi:10.1016/j.earlhumdev.2015.09.008

© 2024 Nagy Zs et al. *JAMA Pediatrics.*

29. Naoshi. The 73rd Annual Congress of the Japan Society of Obstetrics and Gynecology. *Journal of Obstetrics and Gynaecology Research*. 2021;47(8):2800-2934. doi:10.1111/jog.14876

30. Perlman JM, McMenamin JB, Volpe JJ. Fluctuating Cerebral Blood-Flow Velocity in Respiratory-Distress Syndrome. *New England Journal of Medicine*. 1983;309(4):204-209. doi:10.1056/NEJM198307283090402

31. Schreiner C, Hammerl M, Neubauer V, Kiechl-Kohlendorfer U, Griesmaier E. Amplitude-integrated electroencephalography signals in preterm infants with cerebral hemorrhage. *Early Hum Dev*. 2021;154:105309. doi:10.1016/j.earlhumdev.2021.105309

32. Salafia C, Minior V, Rosenkrantz T, et al. Maternal, Placental, and Neonatal Associations with Early Germinal Matrix/Intraventricular Hemorrhage in Infants Born Before 32 Weeks' Gestation. *Am J Perinatol*. 1995;12(06):429-436. doi:10.1055/s-2007-994514

33. Zanardo V, Vedovato S, Suppiej A, et al. Histological Inflammatory Responses in the Placenta and Early Neonatal Brain Injury. *Pediatric and Developmental Pathology*. 2008;11(5):350-354. doi:10.2350/07-08-0324.1

34. Kalani M, Shariat M, Khalesi N, Farahani Z, Ahmadi S. A Comparison of Early Ibuprofen and Indomethacin Administration to Prevent Intraventricular Hemorrhage Among Preterm Infants. *Acta Med Iran*. 2016;54(12):788-792.

35. Najib K, Hashemi Z, Moghtaderi M, Pishdad P, Pishva N, Najib F. Lack of relationship between cord blood erythropoietin and intraventricular hemorrhage in premature neonates: a controversial result. *Child's Nervous System*. 2019;35(2):277-282. doi:10.1007/s00381-018-3872-0

36. Florio P, Perrone S, Luisi S, et al. Increased Plasma Concentrations of Activin A Predict Intraventricular Hemorrhage in Preterm Newborns. *Clin Chem*. 2006;52(8):1516-1521. doi:10.1373/clinchem.2005.065979

37. Galderisi A, Zammataro L, Losiouk E, et al. Continuous Glucose Monitoring Linked to an Artificial Intelligence Risk Index: Early Footprints of Intraventricular Hemorrhage in Preterm Neonates. *Diabetes Technol Ther*. 2019;21(3):146-153. doi:10.1089/dia.2018.0383

© 2024 Nagy Zs et al. *JAMA Pediatrics.*

38. Osborn DA, Evans N, Kluckow M. Hemodynamic and Antecedent Risk Factors of Early and Late Periventricular/Intraventricular Hemorrhage in Premature Infants. *Pediatrics*. 2003;112(1):33-39. doi:10.1542/peds.112.1.33

39. Paradisis M, Evans N, Kluckow M, Osborn D. Randomized Trial of Milrinone Versus Placebo for Prevention of Low Systemic Blood Flow in Very Preterm Infants. *J Pediatr*. 2009;154(2):189-195. doi:10.1016/j.jpeds.2008.07.059

40. Szymonowicz W, Yu VY. Timing and evolution of periventricular haemorrhage in infants weighing 1250 g or less at birth. *Arch Dis Child*. 1984;59(1):7-12. doi:10.1136/adc.59.1.7

41. Tanaka K, Matsumoto S, Minamitani Y, et al. Changes in Internal Cerebral Vein Pulsation and Intraventricular Hemorrhage in Extremely Preterm Infants. *Am J Perinatol*. Published online April 5, 2022. doi:10.1055/a-1817-6638

42. Bel F Van, Valk L, Uiterwaal CSPM, Egberts J, Krediet TG. Plasma guanosine 3',5'-cyclic monophosphate and severity of peri/intraventricular haemorrhage in the preterm newborn. *Acta Paediatr*. 2002;91(4):434-439. doi:10.1080/080352502317371689

43. WEINDLING AM, WILKINSON AR, COOK J, CALVERT SA, FOK T, ROCHEFORT MJ. Perinatal events which precede periventricular haemorrhage and leukomalacia in the newborn. *BJOG*. 1985;92(12):1218-1223. doi:10.1111/j.1471-0528.1985.tb04865.x

44. Iyer KK, Roberts JA, Hellström-Westas L, et al. Early Detection of Preterm Intraventricular Hemorrhage From Clinical Electroencephalography. *Crit Care Med*. 2015;43(10):2219-2227. doi:10.1097/CCM.0000000000001190

45. Popat H, Galea C, Evans N, et al. Effect of Delayed Cord Clamping on Cerebral Oxygenation in Very Preterm Infants. *Neonatology*. 2019;115(1):13-20. doi:10.1159/000492712

46. Thanhaeuser M, Binder C, Derhaschnig U, et al. Can Sequential Coagulation Monitoring Predict Major Haemorrhage in Extremely Low Birth Weight Infants? *Thromb Haemost*. 2018;118(07):1185-1193. doi:10.1055/s-0038-1655744

47. Zanelli SA, Abubakar M, Andris R, Patwardhan K, Fairchild KD, Vesoulis ZA. Early Vital Sign Differences in Very Low Birth Weight Infants with Severe Intraventricular Hemorrhage. *Am J Perinatol*. 2023;40(11):1193-1201. doi:10.1055/s-0041-1733955

© 2024 Nagy Zs et al. *JAMA Pediatrics.*

48. Thorburn RJ, Lipscomb AP, Stewart AL, Reynolds EOR, Hope PL. Timing and antecedents of periventricular haemorrhage and of cerebral atrophy in very preterm infants. *Early Hum Dev*. 1982;7(3):221-238. doi:10.1016/0378-3782(82)90085-8

49. Bates S, Odd D, Luyt K, et al. Superior vena cava flow and intraventricular haemorrhage in extremely preterm infants. *The Journal of Maternal-Fetal & Neonatal Medicine*. 2016;29(10):1581-1587. doi:10.3109/14767058.2015.1054805

50. Yang Y, Jou T, Wu CH, Wang K, Lan C, Shen E. The Obstetric Management in Very-Low-Birth Weight Infants. *Asia Oceania J Obstet Gynaecol*. 1990;16(4):329-335. doi:10.1111/j.1447-0756.1990.tb00357.x

51. Bada HS, Green RS, Pourcyrous M, et al. Indomethacin reduces the risks of severe intraventricular hemorrhage. *J Pediatr*. 1989;115(4):631-637. doi:10.1016/S0022-3476(89)80300-2

52. Lampe R, Rieger-Fackeldey E, Sidorenko I, et al. Assessing key clinical parameters before and after intraventricular hemorrhage in very preterm infants. *Eur J Pediatr*. 2020;179(6):929-937. doi:10.1007/s00431-020-03585-9

53. Rumack CM, Manco-Johnson ML, Manco-Johnson MJ, Koops BL, Hathaway WE, Appareti K. Timing and course of neonatal intracranial hemorrhage using real-time ultrasound. *Radiology*. 1985;154(1):101-105. doi:10.1148/radiology.154.1.3880600

54. Vesoulis ZA, Flower AA, Zanelli S, et al. Blood pressure extremes and severe IVH in preterm infants. *Pediatr Res*. 2020;87(1):69-73. doi:10.1038/s41390-019-0585-3

55. Vesoulis ZA, Whitehead H V., Liao SM, Mathur AM. The hidden consequence of intraventricular hemorrhage: persistent cerebral desaturation after IVH in preterm infants. *Pediatr Res*. 2021;89(4):869-877. doi:10.1038/s41390-020-01189-5

56. Evans N, Kluckow M. Early ductal shunting and intraventricular haemorrhage in ventilated preterm infants. *Arch Dis Child Fetal Neonatal Ed*. 1996;75(3):F183-F186. doi:10.1136/fn.75.3.F183

© 2024 Nagy Zs et al. *JAMA Pediatrics.*

57. da Costa CS, Czosnyka M, Smielewski P, Austin T. Optimal Mean Arterial Blood Pressure in Extremely Preterm Infants within the First 24 Hours of Life. *J Pediatr*. 2018;203:242-248. doi:10.1016/j.jpeds.2018.07.096

58. Paradisis M, Evans N, Kluckow M, Osborn D, McLachlan AJ. Pilot study of milrinone for low systemic blood flow in very preterm infants. *J Pediatr*. 2006;148(3):306-313. doi:10.1016/j.jpeds.2005.11.030

59. Hoffman SB, Cheng YJ, Magder LS, Shet N, Viscardi RM. Cerebral autoregulation in premature infants during the first 96 hours of life and relationship to adverse outcomes. *Arch Dis Child Fetal Neonatal Ed*. 2019;104(5):F473-F479. doi:10.1136/archdischild-2018-315725

60. Juul SE, Comstock BA, Wadhawan R, et al. A Randomized Trial of Erythropoietin for Neuroprotection in Preterm Infants. *New England Journal of Medicine*. 2020;382(3):233-243. doi:10.1056/NEJMoa1907423

61. Martini S, Czosnyka M, Smielewski P, et al. Clinical determinants of cerebrovascular reactivity in very preterm infants during the transitional period. *Pediatr Res*. 2022;92(1):135-141. doi:10.1038/s41390-022-02090-z

62. Espiritu Rojas ER, Zumba K, Guillén-Pinto D. Hemorragia intraventricular en prematuros menores de 28 semanas en un hospital general de Lima, 2013 - 2017. *ACTA MEDICA PERUANA*. 2021;38(1):7-16. doi:10.35663/amp.2021.381.1921

63. Ment LR, Duncan CC, Ehrenkranz RA, et al. Randomized indomethacin trial for prevention of intraventricular hemorrhage in very low birth weight infants. *J Pediatr*. 1985;107(6):937-943. doi:10.1016/S0022-3476(85)80197-9

64. Skubisz A, de Vries LS, Jansen SJ, van der Staaij H, Lopriore E, Steggerda SJ. Early red blood cell transfusion and the occurrence of intraventricular hemorrhage in very preterm infants. *Early Hum Dev*. 2024;189:105926. doi:10.1016/j.earlhumdev.2023.105926

65. Jiang L, Yu Q, Wang F, et al. The role of blood pressure variability indicators combined with cerebral blood flow parameters in predicting intraventricular hemorrhage in very low birth weight preterm infants. *Front Pediatr*. 2023;11. doi:10.3389/fped.2023.1241809

© 2024 Nagy Zs et al. *JAMA Pediatrics.*

66. Xing S, Sun HQ, Li MC. [Clinical characteristics and risk factors of periventricular-intraventricular hemorrhage in extremely low birth weight infants]. *Zhonghua Yi Xue Za Zhi*. 2022;102(47):3774-3778. doi:10.3760/cma.j.cn112137-20220616-01323

67. June A, Heck T, Shah TA, Vazifedan T, Bass WT. Decreased Cerebral Oxygenation in Premature Infants with Progressive Posthemorrhagic Ventricular Dilatation May Help with Timing of Intervention. *Am J Perinatol*. 2023;40(13):1446-1453. doi:10.1055/s-0041-1736533

© 2024 Nagy Zs et al. *JAMA Pediatrics.*
