# Supplementary material for: Occurrence and Time of Onset of Intraventricular Hemorrhage in Preterm Neonates: A Systematic Review and Meta-Analysis of Individual Patient Data
Source: JAMA Pediatr. 2024 Dec 30;179(2):145–54. doi: 10.1001/jamapediatrics.2024.5998 (PMC11791718; doi:10.1001/jamapediatrics.2024.5998)
Supplement: Supplement 2. — Data Sharing Statement [file jamapediatr-e245998-s002.pdf]

Data Sharing Statement

Nagy. Occurrence and Time of Onset of Intraventricular Hemorrhage in Preterm Neonates. *JAMA Pediatr*. Published December 30, 2024. doi:10.1001/jamapediatrics.2024.5998

Data
Data available: Yes
Data types: Other (please specify)
Additional Information: data extracted from eligible studies.
How to access data: manuscript, supplementary material and if more data are required I will send it via e-mail zsuzsanagydr@gmail.com
When available: With publication

Supporting Documents
Document types: None

Additional Information
Who can access the data: anyone requesting the data
Types of analyses: for any purpose
Mechanisms of data availability: with investigator support
